# MC EMiNEM Maps the Interaction Landscape of the Mediator

**DOI:** 10.1371/journal.pcbi.1002568

**Published:** 2012-06-21

**Authors:** Theresa Niederberger, Stefanie Etzold, Michael Lidschreiber, Kerstin C. Maier, Dietmar E. Martin, Holger Fröhlich, Patrick Cramer, Achim Tresch

**Affiliations:** 1 Gene Center Munich and Center for integrated Protein Science CiPSM, Department of Biochemistry, Ludwig-Maximilians-University Munich, Munich, Germany; 2 Bonn-Aachen International Center for IT (B-IT) Algorithmic Bioinformatics, Rheinische Friedrich-Wilhelms-University Bonn, Bonn, Germany; 3 Max Planck Institute for Plant Breeding Research, Cologne, Germany; 4 Institute for Genetics, University of Cologne, Cologne, Germany; Cornell University, United States of America

## Abstract

The Mediator is a highly conserved, large multiprotein complex that is involved essentially in the regulation of eukaryotic mRNA transcription. It acts as a general transcription factor by integrating regulatory signals from gene-specific activators or repressors to the RNA Polymerase II. The internal network of interactions between Mediator subunits that conveys these signals is largely unknown. Here, we introduce MC EMiNEM, a novel method for the retrieval of functional dependencies between proteins that have pleiotropic effects on mRNA transcription. MC EMiNEM is based on Nested Effects Models (NEMs), a class of probabilistic graphical models that extends the idea of hierarchical clustering. It combines mode-hopping Monte Carlo (MC) sampling with an Expectation-Maximization (EM) algorithm for NEMs to increase sensitivity compared to existing methods. A meta-analysis of four Mediator perturbation studies in *Saccharomyces cerevisiae*, three of which are unpublished, provides new insight into the Mediator signaling network. In addition to the known modular organization of the Mediator subunits, MC EMiNEM reveals a hierarchical ordering of its internal information flow, which is putatively transmitted through structural changes within the complex. We identify the N-terminus of Med7 as a peripheral entity, entailing only local structural changes upon perturbation, while the C-terminus of Med7 and Med19 appear to play a central role. MC EMiNEM associates Mediator subunits to most directly affected genes, which, in conjunction with gene set enrichment analysis, allows us to construct an interaction map of Mediator subunits and transcription factors.

## Author Summary

Phenotypic diversity and environmental adaptation in genetically identical cells is achieved by an exact tuning of their transcriptional program. It is a challenging task to unravel parts of the complex network of involved gene regulatory components and their interactions. Here, we shed light on the role of the Mediator complex in transcription regulation in yeast. The Mediator is highly conserved in all eukaryotes and acts as an interface between gene-specific transcription factors and the general mRNA transcription machinery. Even though most of the involved proteins and numerous structural features are already known, details on its functional contribution on basal as well as on activated transcription remain obscure. We use gene expression data, measured upon perturbations of various Mediator subunits, to relate the Mediator structure to the way it processes regulatory information. Moreover, we relate specific subunits to interacting transcription factors.

## Introduction

The Mediator, first discovered by Kim et al. (1994) and Koleske et al. (1994) [1], [2], is a large multiprotein complex which is highly conserved in eukaryotes [3]. Yeast Mediator consists of 25 subunits, organized in 4 different modules: head, middle, tail, and kinase module. It is a general transcription factor (TF) that acts as an interface between gene-specific transcription factors and the core transcription machinery (e.g., Polymerase II). Mediator is required for basal transcription as well as for activated transcription or repression [4]–[6]. In the last years, many successful efforts have been made to gain insight into both structural and functional aspects [7]–[10]. However, though being a well-studied complex, the Mediator still raises a number of unanswered questions: How do the individual subunits contribute to the Mediator's functions? How is the regulatory information transferred within the Mediator complex, and how does it convey these signals to the core transcription machinery?

Recently, “structure-function” analyses have been suggested and conducted by van de Peppel et al. (2005) and Koschubs et al. (2009) [7], [11]. In a clustering approach, they use expression profile similarity as a proxy for physical interaction, respectively for common module membership. Their method was strikingly successful in identifying physical interactions between Mediator subunits. However, it did not exploit the fact that their data originated from active interventions into the cellular system. Such interventions followed by phenotypic measurements of a cell, as opposed to purely observational data, provide additional insight into the functions and interactions of the respective gene products. Along this line, perturbation experiments have been carried out with low-dimensional readouts (such as cell viability or growth [12], [13]) as well as with high-dimensional phenotypes (such as genome-wide expression or DNA binding measurements [14], [15]). While the reconstruction of regulatory networks from observational high-dimensional gene expression data has been investigated thoroughly, e.g., by Basso et al. (2005), Segal et al. (2003) and Segal et al. (2005) [16]–[18], the statistical analysis and interpretation of perturbation data is an active field of research [19], [20]. Nested Effects Models (NEMs) are a class of probabilistic graphical models which are tailored for the analysis of gene expression perturbation screens [21]–[28] (see [29] for a summary). They have been applied successfully to the 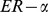 pathway of human MCF-7 breast cancer cells [29] and to a signaling pathway in *Drosophila melanogaster* [21]. Here, we introduce MC EMiNEM, an efficient and robust learning algorithm for NEMs. MC EMiNEM combines a Markov Chain Monte Carlo (MC) sampling procedure with an Expectation-Maximization (EM) algorithm in NEMs. The MC EMiNEM method is freely available as a part of the **R**/*Bioconductor* package *nem*. When applied to gene expression data from various Mediator mutant strains, it reveals parts of the functional architecture of the yeast Mediator complex. Moreover, it predicts new interactions between its subunits and gene-specific transcription factors.

## Methods

### Nested Effects Models

**Figure 1:**
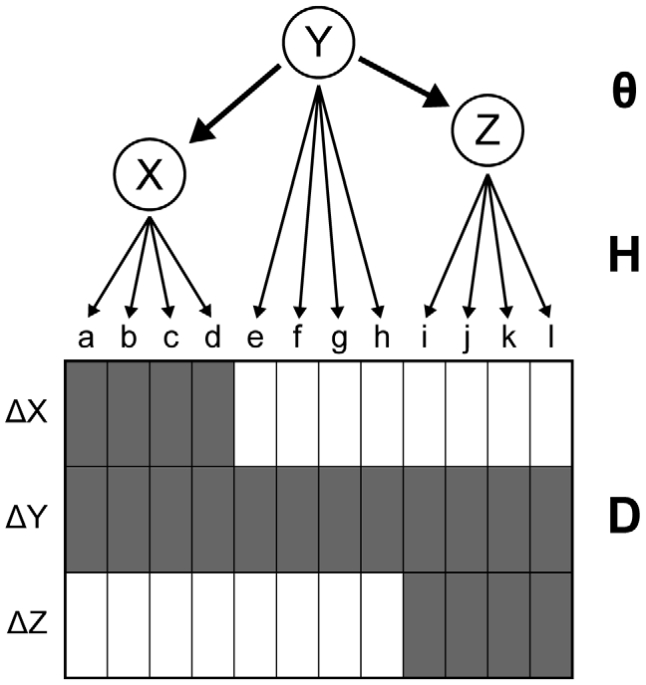
Example NEM. 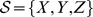
, 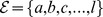. Shaded matrix fields 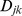 correspond to an expression change of effect gene 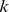 upon perturbation of signal 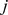, white fields indicate no change in expression. The edges 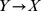 and 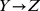 cause an effect in genes directly attached to signal 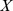 and 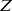 respectively, when 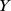 is perturbed.

Nested Effects Models (NEMs) are probabilistic graphical models designed for the analysis of gene expression data from perturbation experiments. They are designed to reconstruct the dependency structure of the perturbation signals, and they perform particularly well if this structure is hierarchical [24]. The graph underlying a NEM contains two types of nodes: the perturbed entities (the signals 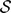) and the genes for which expression has been measured (the effects 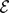). The edges of that graph describe the flow of regulatory information between the nodes. NEMs split this flow into two parts: the signals graph 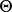 containing the edges between the perturbed entities, and the effects graph 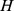 describing the assignment of the effect nodes to the signal nodes. We identify the graphs 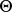 and 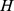 with their respective adjacency matrices 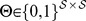, 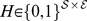. The experimental data is summarized in an 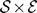 matrix 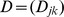, where 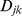 corresponds to the expression data obtained from measurements of effect 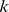 upon perturbation of signal 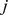. NEMs aim at reconstructing the signals graph, assuming a particularly simple regulatory structure: The perturbation of a signal 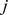 implies the perturbation of other signals that are children of 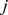. This in turn perturbs the effect nodes that are the children of the perturbed signals in the effects graph (see Fig. 1). In other words, the NEM predicts an effect of gene 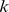 upon perturbation in signal 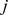 exactly if there is a two-step path from 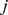 to 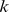, i.e., if 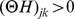. These binary predictions 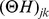 of our model are then linked to the actual measurements by specifying a probability model for the individual effects gene measurements,
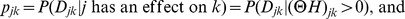


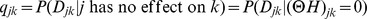
There is extensive literature on the estimation of these two distributions, see [30], [31]. Instead of modeling the two distributions separately, it is convenient to estimate their log ratio. For each effect gene 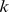, we perform a moderated t-test comparing its expression after perturbation of signal 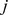 vs. its wild type expression. A false discovery rate estimation procedure is then used to convert the p-values of the moderated t-test into a log odds matrix 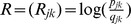. This matrix can for instance be obtained using the **R**/*Bioconductor* package limma (see Section S4.2 in Text S1 for details) [32].

Consequently, a NEM is parametrized by the tuple 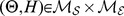, where 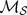 is the space of binary 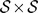 matrices with unit diagonal, and 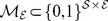 is the space of effects graphs. We assume that the effects graph is *sparse*, such that each effect is linked to at most one signal (i.e., each column of 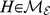 equals either a unit base vector of dimension 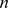, or the null vector). According to Tresch et al. (2008) [25], the log posterior of the signals graph is given by
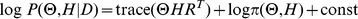
(1)For a derivation of Equation (1), see also Section S1 in Text S1. We assume edge-wise independent priors, 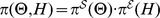, and 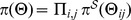, 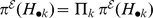. The problem of structure learning in probabilistic graphical models is generally computationally hard (see [33]). A range of methods has been proposed for the maximization of Equation (1). It has been observed that it is very difficult to estimate the effects graph 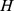 reliably. This is not surprising, since the adjacency matrix 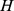 has the same dimensions as the data matrix 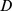. It is therefore desirable to reduce the number of effects a priori. Attaching a gene 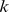 that never has a positive entry 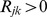 to a signal never increases the posterior. These genes are filtered out prior to the estimation. This step can reduce the number of effects considerably (from about 6000 effects to roughly 3000 in the case of the Mediator experiments). Moreover, we extend the set of signal nodes by a so-called null node, which formally corresponds to extending 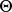 by a null column. Genes that attach to the null node hence are always predicted inactive. This implements an automated feature selection mechanism within the model (see also Section S4.2 in Text S1).

The main objective is the reconstruction of the signals graph 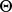. Several approaches try to maximize the (marginal) structure posterior 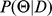 by integrating out the hidden parameters 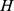 (for a methods review, see [29]). This marginalization however is a time consuming step that increases the complexity of the respective algorithms by at least a factor of 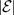, making the analysis of larger effects sets (such as in microarray studies) slow or even impossible. We avoid this drawback and develop an efficient Expectation-Maximization (EM) algorithm for the optimization of the NEM structure posterior (EMiNEM), which, even for large expression data sets, is able to detect a local maximum within seconds. Since the landscape of the structure posterior is rugged (Fig. S2.1 in Text S1), we combine EMiNEM with mode-hopping Markov Chain Monte Carlo (MC EMiNEM) for an efficient optimization of the structure posterior. The MC EMiNEM method is freely available as a part of the **R**/*Bioconductor* package *nem* [34]–[36]. It is easy to use, and it does not require external parameters to be set manually. The only parameter that might be tuned is the weight of the sparsity prior, however moderate changes did not change the outcome qualitatively (see also Sections S2.2 and S5 in Text S1). A short introduction to MC EMiNEM is provided in the Supplements (Section S5 in Text S1, see also the nem package vignette).

### An Expectation-Maximization algorithm for NEMs

Throughout this section, the data 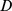 resp. the matrix 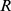 is considered given and fixed. We want to find the maximum a posteriori estimate 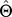 for the signals graph,
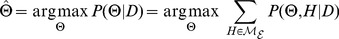
(2)This is the classical situation in which Expectation-Maximization is applicable [37]. For excellent introductions to the EM-algorithm, we recommend the tutorials of Minka (1998), Neal et al. (1998) and Dellaert (2002) [38]–[40]. Briefly, given some guess 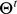 for 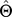, the EM algorithm describes how to find an improved guess 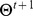 such that the sequence 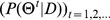 is monotonically increasing, and converges (under mild additional assumptions that are met in our case) to a local maximum of 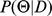.

The expectation (E-)step of the EM algorithm involves calculating the expected log-posterior with respect to the distribution of 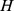, given the current guess 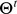:
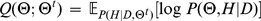
(3)The maximization (M-)step of the EM algorithm then consists of finding the maximizer 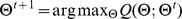. This is usually a much easier task than solving Equation (2) directly. We derive an analytical solution, which leads to an efficient closed-form update step for 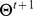:
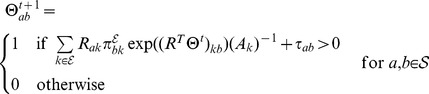
(4)with 
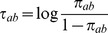
 and 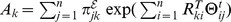. A precise definition of the variables contained in Equation (4), together with a detailed derivation of this formula is deferred to the Supplements, Text S1, as it involves elementary but tedious calculations.

### Sampling of the signal posterior's local maxima

The EM algorithm is guaranteed to find a local maximum which, for unimodal distributions, equals the global optimum. In practice, the posterior landscape 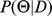 can be very rugged (see also Fig. S2.1 in Text S1). The outcome of the EM algorithm may therefore strongly depend on its initialization, and it may be far from the global optimum (see also Fig. S2.2 in Text S1). This raises the need to explore the set of local maxima provided by EMiNEM. To that end, we introduce MC EMiNEM. In the classical Metropolis-Hastings MCMC approach, consecutive parameter samples 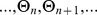 are drawn from the distribution 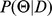. Given 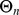, a random process generates a new proposal 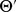. The Hastings ratio, a quantity that involves 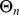 and 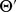, then determines the probability of acceptance (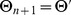) or rejection (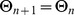) of the new proposal. The MC EMiNEM algorithm instead applies an EM step to each new proposal 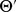, which maps it to the “nearest” local maximum 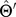. The acceptance/rejection step is then modified by plugging 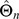 and 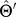 into the Hastings ratio, instead of 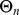 and 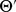. We can show that the series of local maxima 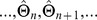 associated to the underlying Markov chain 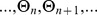 is approximately drawn from 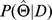, where 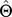 ranges exclusively over the space of local maxima. MC EMiNEM's sampling scheme is illustrated in Fig. S2.3 in Text S1. The details of the implementation as well as a theoretical justification of this method are given in Section S2.2 and S2.3 in Text S1, respectively. Similar so-called mode hopping approaches have been established by Li et al. (1987), Neal et al. (1996), Wales et al. (1997) and Sminchisescu et al. (2003) [41]–[44], with applications in areas such as protein folding [45], nanocluster structure analysis [46] and reconstruction of signaling pathways [47]. Here, we provide a theoretical justification of their use.

### An Empirical Bayes method for the estimation of the signals graph

It is not obvious how the effects graph prior should be defined. Being most conservative, 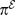 can be chosen uniform, i.e., 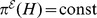 for all effects graphs 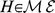. The posterior 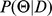 is then proportional to the marginal likelihood 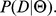 On the other side, upon availability of precise prior knowledge, 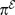 can be chosen deterministic, i.e., 
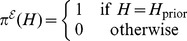
, for some fixed adjacency matrix 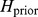. In this case, the posterior is proportional to the full likelihood 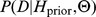. As a trade-off between these two extremes, we initialize 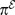 in a data-driven fashion (based on 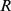), namely
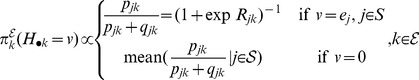
(5)


In an Empirical Bayes approach, we iteratively estimate 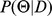 and 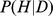, and use these distributions as priors for the estimation of the other quantity, respectively. Our Empirical Bayes procedure is:

1. Initialize 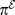 in a data driven fashion (Equation (5)); choose 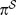 uniform.
2. Generate a representative sample 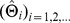 from 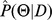 by mode-hopping MCMC, given the prior distributions 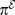 and 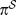.
3. Replace 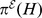 by 
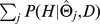
, which is taken as an approximation for 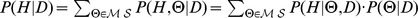. For more details, see Section S2.4 in Text S1.
4. Repeat steps 2 and 3 until convergence (see Sections S3.2 and S4.4 in Text S1).

## Results/Discussion

Our goal was to establish MC EMiNEM as a general purpose tool for the analysis of high-dimensional intervention data, and to use MC EMiNEM for the reconstruction of the internal Mediator complex signaling network. MC EMiNEM includes three key features for an efficient and comprehensive search of the space of candidate regulatory networks (Markov Chain Monte Carlo sampling, in combination with Expectation Maximization, and an Empirical Bayes method for the adaptive attachment of effects). We show in simulations that all these features contribute substantially to the method's performance. Then we construct a high-confidence regulatory network of Mediator subunits. The predicted effects graph reveals interactions between the Mediator and gene-specific transcription factors.

### MC EMiNEM's predictions are accurate in simulations

**Figure 2:**
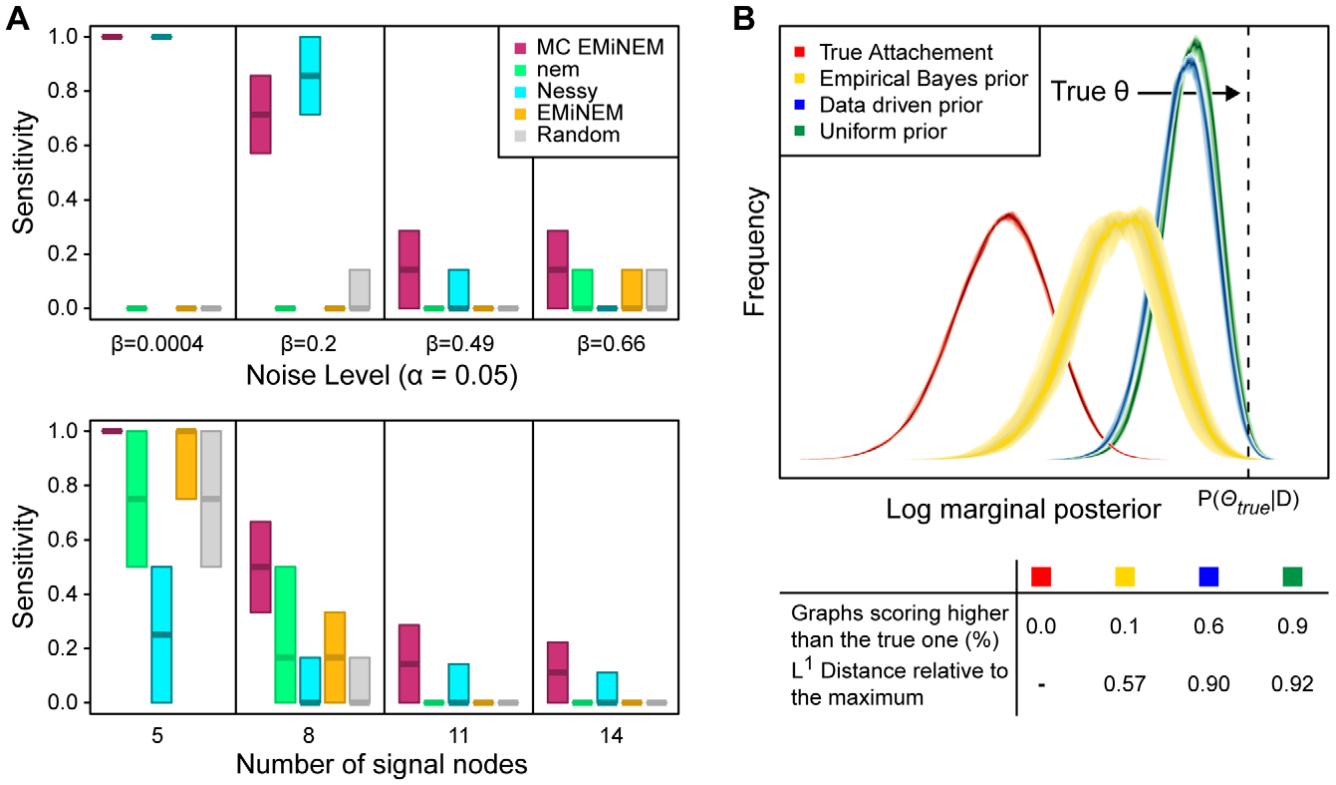
Prediction quality and influence of the Empirical Bayes procedure. (A) Prediction quality. Comparison of the sensitivity of MC EMiNEM and four alternative methods for four different noise levels (top) and four different signals graph sizes (bottom). The sensitivity is depicted on the y-axis, each frame corresponds to one parameter setting. Top: For a signals graph of 11 nodes, noisy data was generated such that for an optimal test with a type-I error (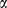-level) of 5%, a type II error (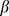-level) of 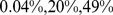, and 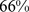 would be achieved, respectively. Bottom: For a noise level corresponding to an error level of (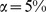, 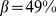), signals graph sizes of 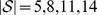 are investigated. We expect our application to range within the four central scenarios. The comparisons of sensitivities is a fair comparison of the prediction qualities since the specificities for all methods and parameter settings are located 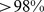 (see also Fig. S3.7 in Text S1). (B) Influence of the Empirical Bayes procedure. Here, for the standard setting 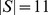 and (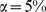, 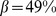). The x-axis shows the calculated marginal posterior values 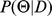 centered at 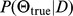 (indicated by the dashed vertical line), on the y-axis the frequency is displayed. In the table, the percentages of signals graphs scoring higher than 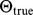 are provided, as well as the 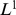-distances (relative to the maximum).

Extensive simulations were performed to ensure the convergence of the MCMC chain, and to verify the independence of the outcome from the initial parameter choice (see Section S2.2 in Text S1). The prediction quality was assessed in seven parameter settings for different noise levels and different numbers of signal nodes, with 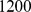 observed effect genes and a total number of 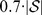 edges in the signals graph. For each of these scenarios, 50 NEMs were randomly sampled (for details see Section S3.1 in Text S1). In each case, data was generated and afterwards analyzed with various methods: a simple EMiNEM approach without Markov Chain Monte Carlo sampling, the original NEM score [21], the Nessy method [25] and a random sampling approach (for details on the competing methods see Section S3.3 in Text S1). For all methods, the sensitivity strongly depends on the noise level and the number of signal nodes (Fig. 2A). MC EMiNEM performs best throughout all tested parameter settings, except for low noise where Nessy achieves a similar sensitivity. The specificity of all methods is very high, with a value above 98% in all scenarios (see also Fig. S3.7 in Text S1). A comparison of the method-specific run times is provided in Table S1 in Text S1. It should be mentioned that EMiNEM itself is extremely efficient, even for large numbers of effect nodes (one run for the Mediator data took 0.1 s on a standard desktop computer). This efficiency is a prerequisite that allows us to perform ten thousands of MCMC steps in the MC EMiNEM algorithm in an acceptable time. For a comparison of run times and scalability of the different methods, see Table S1 in Text S1.

### Adaptive attachment of effects improves prediction quality

Our approach attempts to maximize the marginal posterior 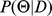. This quantity implicitly depends on the effects graph prior 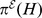. Therefore, we seek a prior for which the true signals graph 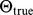 scores on the top end of the distribution 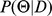. It has been shown that NEM models are asymptotically consistent and identifiable [25], i.e., given the true effects graph as a deterministic prior 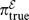, the true signals graph will score best. Thus, a well-chosen effects gene prior might greatly improve the prediction outcome. We tested the following priors: a deterministic prior according to the true effects graph, our Empirical Bayes prior, the data-driven prior used for the initialization of the MCMC sampling (see S2.4), and a uniform effects graph prior. The quality of an effects graph prior is assessed in two ways: First, we calculate the average 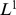-distance between the prior 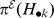 to the true prior 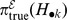, where 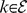, and normalize it by dividing through the maximum gene-wise 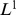-distance, which is 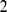. Secondly, we calculate the position of 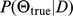 within the marginal posterior distribution 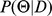. Each posterior distribution was approximated by the empirical distribution of 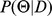 for a random sample of 5000 signals graphs. This was done for the 50 NEM samples that were generated in the most realistic simulation scenario (11 nodes, 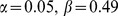, see Fig. 2 A). The results show that the Empirical Bayes prior approaches the true prior better than the other methods, according to the 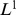-distances. Furthermore, the resulting posterior is better able to distinguish between signals graphs and to identify the true one (the true graph is located at the 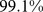, 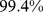, and 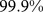 quantile for the uniform, data driven and Empirical Bayes prior, respectively, and at the maximum for the true effects graph; see Fig. 2 B).

### MC EMiNEM predicts a robust Mediator subunit network

**Figure 3:**
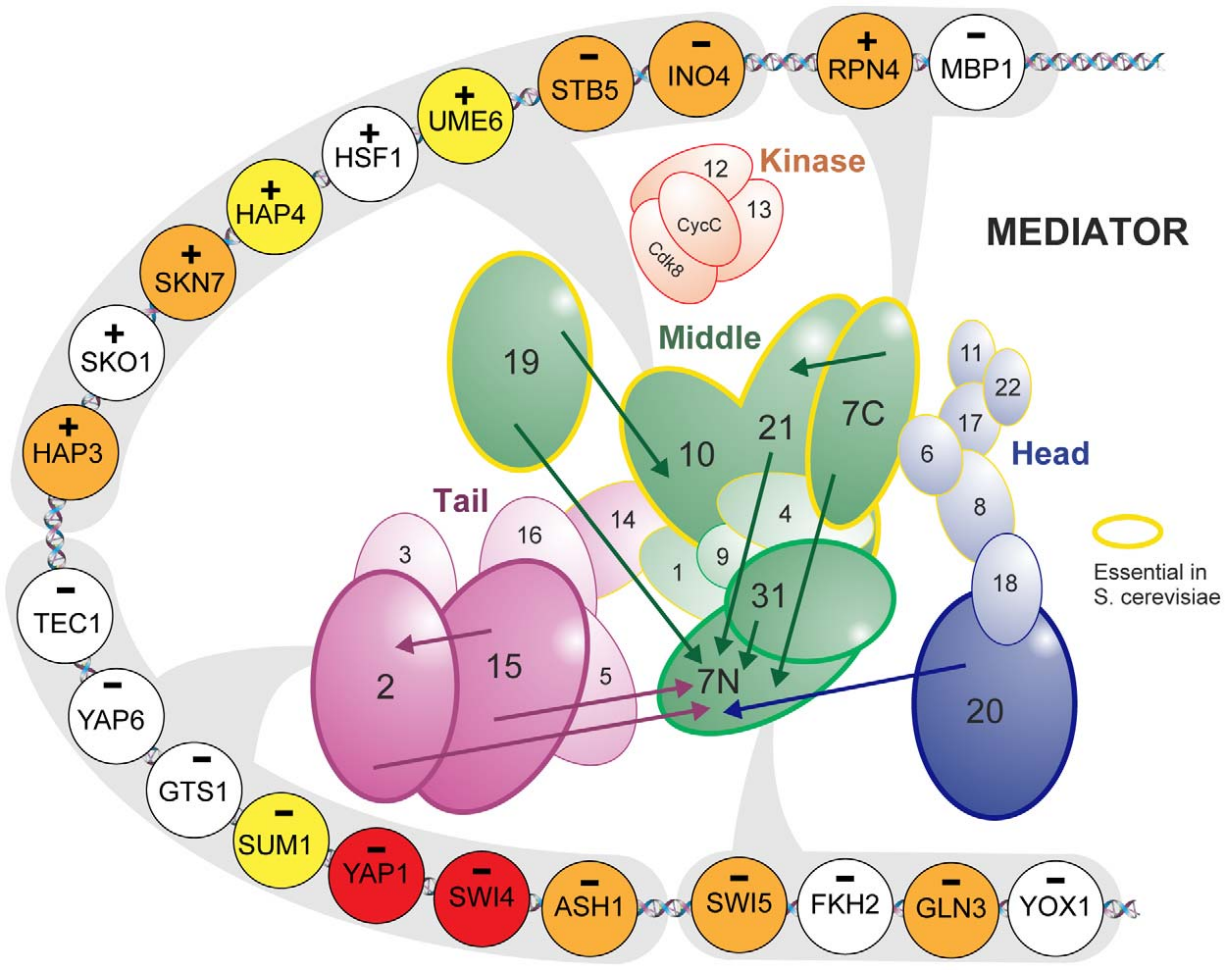
Mediator network inferred by MC EMiNEM, with associated transcription factors (the basic Mediator cartoon was modified from [63]). The numbers of the Mediator subunits correspond to the unified Mediator nomenclature [64] and subunits that are part of this study are enlarged and have saturated colors. The two subunits Med10 and Med21 were merged as explained in the main text. The N-terminus and the C-terminus of Med7, which are represented by two individual perturbations in this study, are shown separately. Physically, they are connected by a flexible linker [8]. The arrows between the Mediator subunits show the signals graph of our MC EMiNEM analysis, arrow colors correspond to the module they originate from. The TFs surrounding the Mediator are the outcome of a gene set enrichment analysis of the MC EMiNEM effects graph. TFs are grouped into gray areas which link them to the Mediator subunit for whose target genes they are enriched. For each TF, minus resp. plus signs indicate whether their targets are down- resp. upregulated upon perturbation of the corresponding Mediator subunit. The results of the gene set enrichment analysis were compared to known interactions between TFs and Mediator subunits in BioGRID [60], [65]). Red: the interaction with the corresponding Mediator subunit is known; orange: an interaction with a Mediator subunit in the same module is known; dark yellow: confirmed interaction with the Mediator; white: no known interaction.

The 25 protein subunits of the Mediator are subdivided into 4 distinct modules (head, middle, tail, kinase, see Fig. 3). The tail module is believed to establish the contact to the gene-specific transcription factors, based on various TF binding domains, while the head and middle module apparently contact Polymerase II [48]. The kinase module is described as having mostly inhibitory effects on gene expression [49]. The perturbation of a central Mediator subunit can have severe consequences on the structure of the whole Mediator complex. It may cause the loss of whole modules or specific submodules [50]–[52]. The perturbation of a peripheral component might have only local effects on the Mediator structure and, consequently, have fewer effects on transcription. From the structural organization of the Mediator, we therefore expect a hierarchy of transcriptional effects upon subunit perturbations, which makes NEMs a suitable tool for their analysis. As a result of a NEM analysis, we expect the central Mediator subunits that have widespread effects upstream in the signals graph, whereas the more peripheral components should lie downstream. Due to its role as a general transcription factor involved in the formation of the transcription initiation complex, a perturbation of the Mediator can entail global changes in gene expression [53]. Such effects are completely removed by our normalization procedure and can therefore not be detected. Note that systematic variation in RNA extraction, RNA amplification, labeling and scanner calibration make it generally impossible to reliably detect global shifts in transcriptional activity by conventional methods; the absolute quantification of transcription levels requires new experimental techniques, e.g., as proposed in Sun et al. [54]. Our focus in the present study, however, is on effects that are due to the interaction of the Mediator with gene-specific transcription factors. These effects are restricted to the target genes of the interacting transcription factors. They superimpose to the possible global effects of a Mediator perturbation, and hence become visible only after removal of the global effects.

We generated expression profiles of *S.cerevisiae* Mediator subunit deletion mutants dMed2, dMed15, dMed20, dMed31, which were complemented by data from published intervention studies on the Mediator. Those comprise mutations of Med7 (N- and C-terminal deletion), and point mutants of Med10, Med19, Med20, Med21 (see S4.1). The raw data is available at ArrayExpress (accession number E-MTAB-1037). Although there exist even more high-quality gene expression data of Mediator mutants (e.g., [52], [55]), we restricted our analysis to experiments that were obtained on the Affymetrix yeast 2.0 array under similar environmental conditions. Luckily, some data were redundant in different experiments, which enabled us to correct for batch-specific effects, and to remove outlier genes (for data pre-processing, see Section S4.2 in Text S1). After normalization and batch effect removal, a straightforward application of the MC EMiNEM algorithm led to identical results in 9 out of 10 independent MCMC runs; the tenth run differed only by one edge (Fig. S4.1, Fig. S4.2 in Text S1). The runs revealed a bi-directional edge assigned to the Med10 and Med21 nodes, which means that these two subunits are indistinguishable in terms of their intervention effects. Their attached effect genes are interchangeable without affecting the model's likelihood. Therefore, according to Tresch et al. (2008) [25], we combine the two subunits and treat them as one node (see Section S4.2 in Text S1). When Med10 and Med21 were combined, 10 independent MC EMiNEM runs gave identical signals graph predictions (Fig. 3). The corresponding attachment of effects to signal nodes is provided in Dataset S1.

### MC EMiNEM confirms the Mediator architecture

**Figure 4:**
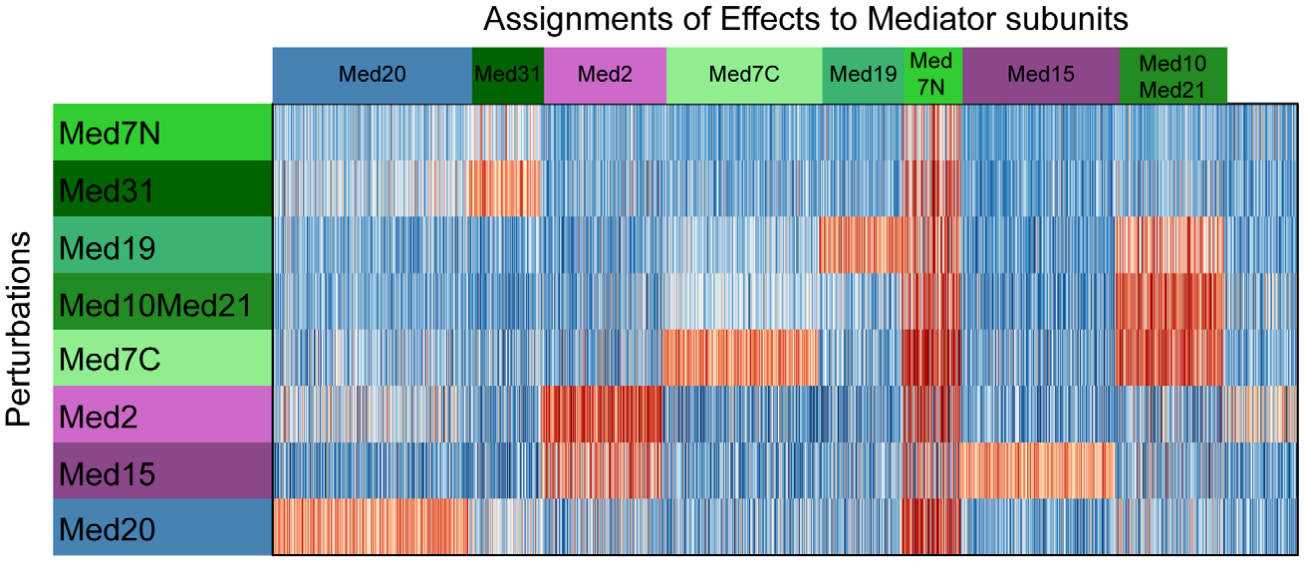
Effects graph inferred from the Mediator data. Shown are the log-odds ratios which serve as MC EMiNEM's input. Genes that are likely to change in a given condition are depicted in red,and they are blue otherwise. Color saturation indicates the absolute value of the log-odds ratio (cf. Fig. S4.3 in Text S1). Rows correspond to Mediator perturbation experiments, columns correspond to genes, sorted according to their attachment to Mediator subunits. Mediator subunits are colored as in Fig. 3 and Fig. 5.

The predicted Mediator network (the signals graph in Fig. 3) agrees well with current knowledge about the Mediator structure [8], [10]: When removing the downstream Med7N node, the signals graph is separated into three connected components that reflect the modular organization of the Mediator (middle module: Med7C, Med19, Med10Med21, Med31; head module: Med20; tail module: Med2, Med15). While the overall module organization of the Mediator can also be recovered from a simple clustering analysis (see Section S4.4 in Text S1), MC EMiNEM reveals a much finer structure by assigning a directionality to each edge. Med7N is downstream of all other nodes, indicating that among all perturbations that were applied, it has the fewest effects on transcription. It shows that there is a set of effects (attached to Med7N in the NEM) whose transcription depends on an entirely intact Mediator complex. The middle module component consists of a Med7C, Med10Med21 and Med19 upstream part, and a Med31, Med7N downstream part. Again, this conforms to its physical architecture: Med7C/Med10Med21 and Med7N/Med31 form stable complexes [8]. We conclude that the former are central architectural components, whereas the latter are peripheral. Indeed, Med7N/Med31 are only weakly attached to the middle module, and easily dissociate from it, whereas Med7C/Med10Med21 are essential for its architecture [8]. The position of Med19 yet is still unclear [56], [57]. In our model, however, Med19 is clearly placed in the center of the middle module. The tail module interacts with gene-specific transcription factors and is structurally less analyzed [6]. The NEM includes an edge from Med15 to Med2 and thus suggests a more central role for Med15 than for Med2, because the effects upon perturbation of Med2 are a subset of the respective Med15 effects (see Fig. 4 and Fig. S4.3 in Text S1).

### MC EMiNEM provides a map of specific transcription factor - Mediator interactions

Apart from an estimate of the internal flow of regulatory information in the signals graph, MC EMiNEM returns a posterior probability of the attachment of effect genes to specific Mediator subunits (Fig. 4). The attachment of effects to signal nodes in the NEM framework does not necessarily represent a physical/direct interaction of the Mediator with the DNA. In the case of the Mediator it is sensible to assume that the coupling is mediated by transcription factors (TFs). We extend the analysis of our Mediator network and infer the transcription factors by which this coupling has been achieved (cf. [28]). We group the effect genes according to their attachment to signal nodes and according to the direction of expression change upon perturbation. A gene set enrichment analysis for these 16 groups then reveals interactions of gene-specific TFs with specific Mediator subunits/submodules. We used the MGSA algorithm for the enrichment analysis [58], based on the gene-TF assignment by Mac Isaac et al. (2006) [59] (see also Section S4.3 in Text S1). Although the attachment of individual effects to Mediator subunits is notoriously variable (see Fig. S3.5 and S4.6 in Text S1), the gene set enrichment approach lends its robustness from combining evidence from many attached genes. The result is a map of TF-Mediator interactions, summarized in Fig. 3 and listed in Table S2 in Text S1.

The 21 TF-Mediator subunit interactions mapped by MC EMiNEM were validated using the BioGRID database [60]. Two interaction pairs were known from the literature (YAP1-Med2, SWI4-Med2). Another eight TFs were known to interact with a Mediator subunit from the same module as the predicted interacting subunit ([GLN3/SWI5]-Med7N, RPN4-Med7C, [SKN7/STB5/INO4/HAP3]-Med10Med21, ASH1-Med2). An interaction with the Mediator has been described for three more TFs ([UME6/HAP4]-Med10Med21, SUM1-Med2), and eight predicted interactions were new (MBP1-Med7C, [HSF1/SKO1]-Med10Med21, [TEC1/YAP6/GTS1]-Med2, [FKH2/YOX1]-Med7N).

All target genes of TFs associated with the tail module show downregulation after perturbation, consistent with the tail's function to contact gene specific transcription factors [5]. The same holds for the target genes of TFs associated with Med7N. This is expected, as the genes attached to Med7N are those that show an effect in all perturbations (Fig. 4) and therefore presumably require a completely intact Mediator. The target genes of TFs associated to the rest of the middle module show expression changes in both directions, in accordance with the middle module described as an ambiguous regulator [11].

**Figure 5:**
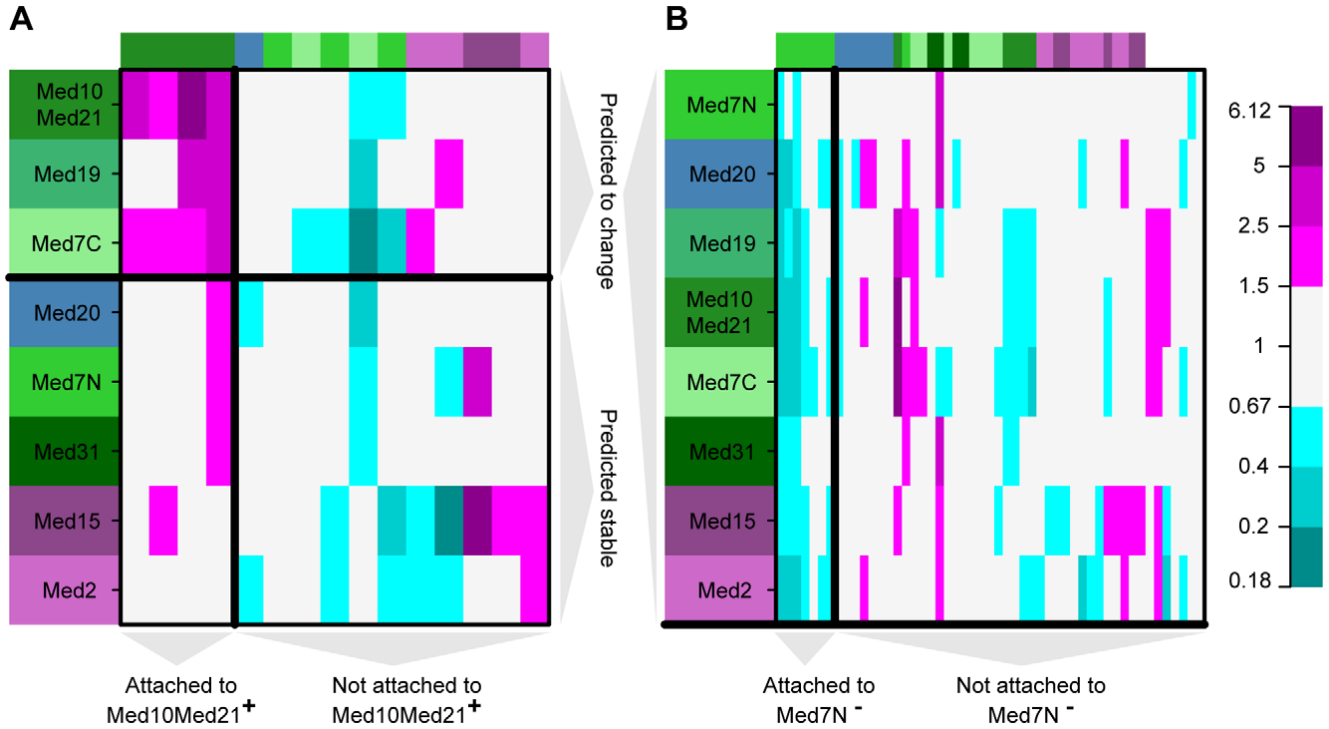
Gene set enrichment analysis. A) Expression changes of the target genes of SKO1 across all experiments. Experiments correspond to rows; the respective Mediator subunit perturbations are indicated by the colored boxes to the left of the heat map (coloring is in accordance with the Mediator module structure in Fig. 3). Target genes correspond to columns. If a target gene is attached to a Mediator subunit in the MC EMiNEM effects graph, this is indicated by a colored box on top of the respective column, using the same color code as for the experiments. Expression changes relative to wild type are color coded by the panel on the right. In the gene set enrichment analysis, SKO1 target genes were found enriched for upregulated genes attached to the Med10Med21 node in the MC EMiNEM effects graph. These genes lie to the left of the bold vertical line in the heat map. Briefly, our Mediator NEM model predicts that they should also change their expression in the Med19 and Med7C perturbations, which lie above the bold horizontal line. Ideally, the expression changes in the upper left corner defined by the two bold lines should be strong and consistent, while those in the remaining part should be weaker and less consistent. B) Same plot as A), for the target genes of SWI5. Since SWI5 targets are enriched for downregulated genes attached to Med7N, and Med7N is downstream of all other nodes in the signals graph, we expect consistent expression changes of the Med7N attached genes across all perturbations.

Fig. 5 A) offers a TF-centric excerpt on the MC EMiNEM map from Fig. 4. It drills in to the target genes of SKO1, which are enriched in the set of upregulated genes attached to Med10Med21. SKO1 is both a transcriptional activator and repressor and forms a complex with the general repressor TUP1 (Saccharomyces Genome Database [61]). TUP1 in turn targets Med21p [62]. A Mediator complex lacking this subunit might thus not be able to forward repressive signals, resulting in upregulated target genes of SKO1.

The transcriptional activator SWI5 has a large number of physical interactions with subunits from various Mediator modules (Med15, Med17, Med18, Med22, [61]). This suggests that any change in the Mediator structure affects its interaction with SWI5. Consequently, target genes of SWI5 should change their expression upon any Mediator subunit perturbation. Fig. 5 B) confirms this behavior of the SWI5 targets: MC EMiNEM associates SWI5 to Med7N, because SWI5 targets are enriched in the set of downregulated genes attached to Med7N, and these are consistently downregulated in all perturbations.

Similar analyses were carried out for all TFs in the MC EMiNEM map (Figure S1; lists of genes that contribute to the respective TF enrichments are provided in Dataset S2). The most striking observation is that the sign of a gene's expression change is consistent in virtually all perturbations for which MC EMiNEM predicts an effect. Since our model is completely blind with respect to the sign of regulation, the consistency in the direction of the expression changes provides compelling evidence that the signals graph reflects regulatory dependencies between Mediator subunits which are likely to be caused by structural changes.

### Conclusion

The reconstruction of interaction networks from high dimensional perturbation effects is still a challenge. We have developed MC EMiNEM, a method for the learning of a Nested Effects Model. We introduced two major improvements, namely an Expectation-Maximization algorithm for the very fast detection of local maxima of the posterior probability function. Mode hopping Markov Chain Monte Carlo sampling was then used for the efficient exploration of the space of local maxima. We applied MC EMiNEM to a combination of proper and public gene expression data obtained from Mediator subunit perturbations. It turned out that MC EMiNEM does not only shed light on structural dependencies of Mediator subunits, it also identifies interactions of gene-specific transcription factors with Mediator subunits. Our findings are consistent with the state-of-the-art knowledge about the Mediator architecture and function. By grouping of components with similar profiles, hierarchical clustering has proved tremendously useful for the analysis of expression data obtained from observational experiments. MC EMiNEM reaches beyond the identification of undirected relationships; it resolves directed regulatory structures, and it identifies gene groups with a consistent and specific response pattern. For interventional data, MC EMiNEM is thus the appropriate counterpart to clustering.

## Supporting Information

Dataset S1 **Attachment of effects to signal nodes.** The attachment of effects to signal nodes displayed in Figure 4. A tab-separated text file, where the first column corresponds to the Mediator subunit and the second column corresponds to the attached gene.
(TXT)

Dataset S2 **Gene set enrichment analysis.** The lists of genes that contribute to the respective TF-Mediator subunit interactions derived from the gene set enrichment analysis (see also Figure 3 and Figure 5). A tab-separated text file, where the first column corresponds to the Mediator subunit, the second column corresponds to the direction of expression change of the respective gene set, the third column corresponds to the interacting TF and the fourth column corresponds to the targets of the TF that are attached to the respective Mediator subunit.
(TXT)

Figure S1 **TF-Mediator subunit interactions.** For each TF-Mediator subunit interaction predicted by the gene set enrichment analysis (see Figure 3), a figure similar to Figure 5 is provided. For more information, please refer to the legend of Figure 5.
(PDF)

Text S1 **Additional information on methods and results.** This file provides additional information on methods and results which go beyond the scope of this paper, including detailed derivations of formulas.
(PDF)

## References

[1] Kim YJ, Björklund S, Li Y, Sayre MH, Kornberg RD (1994). A multiprotein mediator of transcriptional activation and its interaction with the C-terminal repeat domain of RNA polymerase II.. Cell.

[2] Koleske AJ, Young RA (1994). An RNA polymerase II holoenzyme responsive to activators.. Nature.

[3] Bourbon HM (2008). Comparative genomics supports a deep evolutionary origin for the large, four-module transcriptional mediator complex.. Nucleic Acids Res.

[4] Kornberg RD (2005). Mediator and the mechanism of transcriptional activation.. Trends Biochem Sci.

[5] Conaway RC, Conaway JW (2011). Origins and activity of the Mediator complex.. Semin Cell Dev Biol.

[6] Larivière L, Seizl M, Cramer P (2012). A structural perspective on Mediator function.. Curr Opin Cell Biol.

[7] Koschubs T, Seizl M, Larivière L, Kurth F, Baumli S (2009). Identification, structure, and functional requirement of the Mediator submodule Med7N/31.. EMBO J.

[8] Koschubs T, Lorenzen K, Baumli S, Sandström S, Heck AJR (2010). Preparation and topology of the Mediator middle module.. Nucleic Acids Res.

[9] Borggrefe T, Yue X (2011). Interactions between subunits of the Mediator complex with genespecific transcription factors.. Semin Cell Dev Biol.

[10] Imasaki T, Calero G, Cai G, Tsai KL, Yamada K (2011). Architecture of the Mediator head module.. Nature.

[11] van de Peppel J, Kettelarij N, van Bakel H, Kockelkorn TTJP, van Leenen D (2005). Mediator expression profiling epistasis reveals a signal transduction pathway with antagonistic submodules and highly specific downstream targets.. Mol Cell.

[12] Winzeler EA, Shoemaker DD, Astromoff A, Liang H, Anderson K (1999). Functional characterization of the S. cerevisiae genome by gene deletion and parallel analysis.. Science.

[13] Giaever G, Chu AM, Ni L, Connelly C, Riles L (2002). Functional profiling of the Saccharomyces cerevisiae genome.. Nature.

[14] Hughes TR, Marton MJ, Jones AR, Roberts CJ, Stoughton R (2000). Functional discovery via a compendium of expression profiles.. Cell.

[15] Hu Z, Killion PJ, Iyer VR (2007). Genetic reconstruction of a functional transcriptional regulatory network.. Nat Genet.

[16] Basso K, Margolin AA, Stolovitzky G, Klein U, Dalla-Favera R (2005). Reverse engineering of regulatory networks in human B cells.. Nat Genet.

[17] Segal E, Shapira M, Regev A, Pe'er D, Botstein D (2003). Module networks: identifying regulatory modules and their condition-specific regulators from gene expression data.. Nat Genet.

[18] Segal E, Friedman N, Kaminski N, Regev A, Koller D (2005). From signatures to models: understanding cancer using microarrays.. Nat Genet.

[19] Yeang CH, Mak HC, McCuine S, Workman C, Jaakkola T (2005). Validation and refinement of gene-regulatory pathways on a network of physical interactions.. Genome Biol.

[20] Gao F, Foat BC, Bussemaker HJ (2004). Defining transcriptional networks through integrative modeling of mRNA expression and transcription factor binding data.. BMC Bioinformatics.

[21] Markowetz F, Bloch J, Spang R (2005). Non-transcriptional pathway features reconstructed from secondary effects of RNA interference.. Bioinformatics.

[22] Fröhlich H, Fellmann M, Sültmann H, Poustka A, Beissbarth T (2007). Large scale statistical inference of signaling pathways from RNAi and microarray data.. BMC Bioinformatics.

[23] Fröhlich H, Fellmann M, Sültmann H, Poustka A, Beissbarth T (2008). Estimating large-scale signaling networks through nested effect models with intervention effects from microarray data.. Bioinformatics.

[24] Markowetz F, Kostka D, Troyanskaya OG, Spang R (2007). Nested effects models for highdimensional phenotyping screens.. Bioinformatics.

[25] Tresch A, Markowetz F (2008). Structure learning in Nested Effects Models.. Stat Appl Genet Mol Biol.

[26] Anchang B, Sadeh MJ, Jacob J, Tresch A, Vlad MO (2009). Modeling the temporal interplay of molecular signaling and gene expression by using dynamic nested effects models.. Proc Natl Acad Sci U S A.

[27] Zeller C, Fröhlich H, Tresch A (2008). A bayesian network view on nested effects models.. EURASIP J Bioinform Syst Biol.

[28] Vaske CJ, House C, Luu T, Frank B, Yeang CH (2009). A factor graph nested effects model to identify networks from genetic perturbations.. PLoS Comput Biol.

[29] Fröhlich H, Tresch A, Beissbarth T (2009). Nested effects models for learning signaling networks from perturbation data.. Biom J.

[30] Smyth GK (2004). Linear models and empirical bayes methods for assessing differential expression in microarray experiments.. Stat Appl Genet Mol Biol.

[31] Storey JD, Tibshirani R (2003). Statistical significance for genomewide studies.. Proc Natl Acad Sci U S A.

[32] Smyth GK, Gentleman R, Carey V, Dudoit S, R Irizarry WH (2005). Limma: linear models for microarray data.. Bioinformatics and Computational Biology Solutions using R and Bioconductor.

[33] Lauritzen S (1996). Graphical models.

[34] Ihaka R, Gentleman R (1996). R: A Language for Data Analysis and Graphics.. J Comput Graph Stat.

[35] Gentleman RC, Carey VJ, Bates DM, Bolstad B, Dettling M (2004). Bioconductor: open software development for computational biology and bioinformatics.. Genome Biol.

[36] Fröhlich H, Markowetz F, Tresch A, Niederberger T, Bender C (2012). nem: Nested Effects Models to reconstruct phenotypic hierarchies, version 2.32.0.. Bioconductor.

[37] Dempster AP, Laird NM, Rubin DB (1977). Maximum Likelihood from Incomplete Data via the EM Algorithm.. J R Stat Soc Series B Stat Methodol.

[38] Minka TP (1998). Expectation-Maximization as lower bound maximization..

[39] Neal RM, Hinton GE, Jordan M (1998). A view of the EM algorithm that justifies incremental sparse and other variants.. Learning in Graphical Models.

[40] Dellaert F (2002). The Expectation Maximization Algorithm..

[41] Li Z, Scheraga HA (1987). Monte Carlo-minimization approach to the multiple-minima problem in protein folding.. Proc Natl Acad Sci U S A.

[42] Neal RM (1996). Sampling from multimodal distributions using tempered transitions.. Stat Comput.

[43] Wales DJ, Doye JPK (1997). Global Optimization by Basin-Hopping and the Lowest Energy Structures of Lennard-Jones Clusters Containing up to 110 Atoms.. J Phys Chem.

[44] Sminchisescu C, Welling M, Hinton G (2003). A Mode-Hopping MCMC sampler..

[45] Carr JM, Wales DJ (2005). Global optimization and folding pathways of selected alpha-helical proteins.. J Chem Phys.

[46] Kim HG, Choi SK, Lee HM (2008). New algorithm in the basin hopping Monte Carlo to find the global minimum structure of unary and binary metallic nanoclusters.. J Chem Phys.

[47] Kaderali L, Dazert E, Zeuge U, Frese M, Bartenschlager R (2009). Reconstructing signaling pathways from RNAi data using probabilistic Boolean threshold networks.. Bioinformatics.

[48] Soutourina J, Wydau S, Ambroise Y, Boschiero C, Werner M (2011). Direct interaction of RNA polymerase II and mediator required for transcription in vivo.. Science.

[49] Elmlund H, Baraznenok V, Lindahl M, Samuelsen CO, Koeck PJB (2006). The cyclindependent kinase 8 module sterically blocks Mediator interactions with RNA polymerase II.. Proc Natl Acad Sci U S A.

[50] Zhang F, Sumibcay L, Hinnebusch AG, Swanson MJ (2004). A triad of subunits from the Gal11/tail domain of Srb mediator is an in vivo target of transcriptional activator Gcn4p.. Mol Cell Biol.

[51] Takagi Y, Kornberg RD (2006). Mediator as a general transcription factor.. J Biol Chem.

[52] Larivière L, Seizl M, van Wageningen S, Röther S, van de Pasch L (2008). Structure-system correlation identifies a gene regulatory Mediator submodule.. Genes Dev.

[53] Holstege FC, Jennings EG, Wyrick JJ, Lee TI, Hengartner CJ (1998). Dissecting the regulatory circuitry of a eukaryotic genome.. Cell.

[54] Sun M, Schwalb B, Schulz D, Pirkl N, Etzold S (2012). Comparative dynamic transcriptome analysis (cDTA) reveals mutual feedback between mRNA synthesis and degradation.. Genome Res.

[55] Ansari SA, Ganapathi M, Benschop JJ, Holstege FCP, Wade JT (2012). Distinct role of Mediator tail module in regulation of SAGA-dependent, TATA-containing genes in yeast.. EMBO J.

[56] Takagi Y, Calero G, Komori H, Brown JA, Ehrensberger AH (2006). Head module control of mediator interactions.. Mol Cell.

[57] Baidoobonso SM, Guidi BW, Myers LC (2007). Med19(Rox3) regulates Intermodule interactions in the Saccharomyces cerevisiae mediator complex.. J Biol Chem.

[58] Bauer S, Gagneur J, Robinson PN (2010). GOing Bayesian: model-based gene set analysis of genome-scale data.. Nucleic Acids Res.

[59] MacIsaac KD, Wang T, Gordon DB, Gifford DK, Stormo GD (2006). An improved map of conserved regulatory sites for Saccharomyces cerevisiae.. BMC Bioinformatics.

[60] Stark C, Breitkreutz BJ, Chatr-Aryamontri A, Boucher L, Oughtred R (2011). The BioGRID Interaction Database: 2011 update.. Nucleic Acids Res.

[61] Cherry JM, Hong EL, Amundsen C, Balakrishnan R, Binkley G (2012). Saccharomyces Genome Database: the genomics resource of budding yeast.. Nucleic Acids Res.

[62] Gromöller A, Lehming N (2000). Srb7p is a physical and physiological target of Tup1p.. EMBO J.

[63] Koschubs T (2010). Structure and functional architecture of the Mediator middle module from budding yeast..

[64] Bourbon HM, Aguilera A, Ansari AZ, Asturias FJ, Berk AJ (2004). A unified nomenclature for protein subunits of mediator complexes linking transcriptional regulators to RNA polymerase II.. Mol Cell.

[65] Stark C, Breitkreutz BJ, Reguly T, Boucher L, Breitkreutz A (2006). BioGRID: a general repository for interaction datasets.. Nucleic Acids Res.

