# Supplementary material for: MC EMiNEM Maps the Interaction Landscape of the Mediator
Source: PLoS Comput Biol. 2012 Jun 21;8(6):e1002568. doi: 10.1371/journal.pcbi.1002568 (PMC3380870; doi:10.1371/journal.pcbi.1002568)
Supplement: Figure S1 — TF-Mediator subunit interactions. For each TF-Mediator subunit interaction predicted by the gene set enrichment analysis (see Figure 3), a figure similar to Figure 5 is provided. For more information, please refer to the legend of Figure 5. (PDF) [file pcbi.1002568.s003.pdf]

Med10Med21, downregulated: INO4

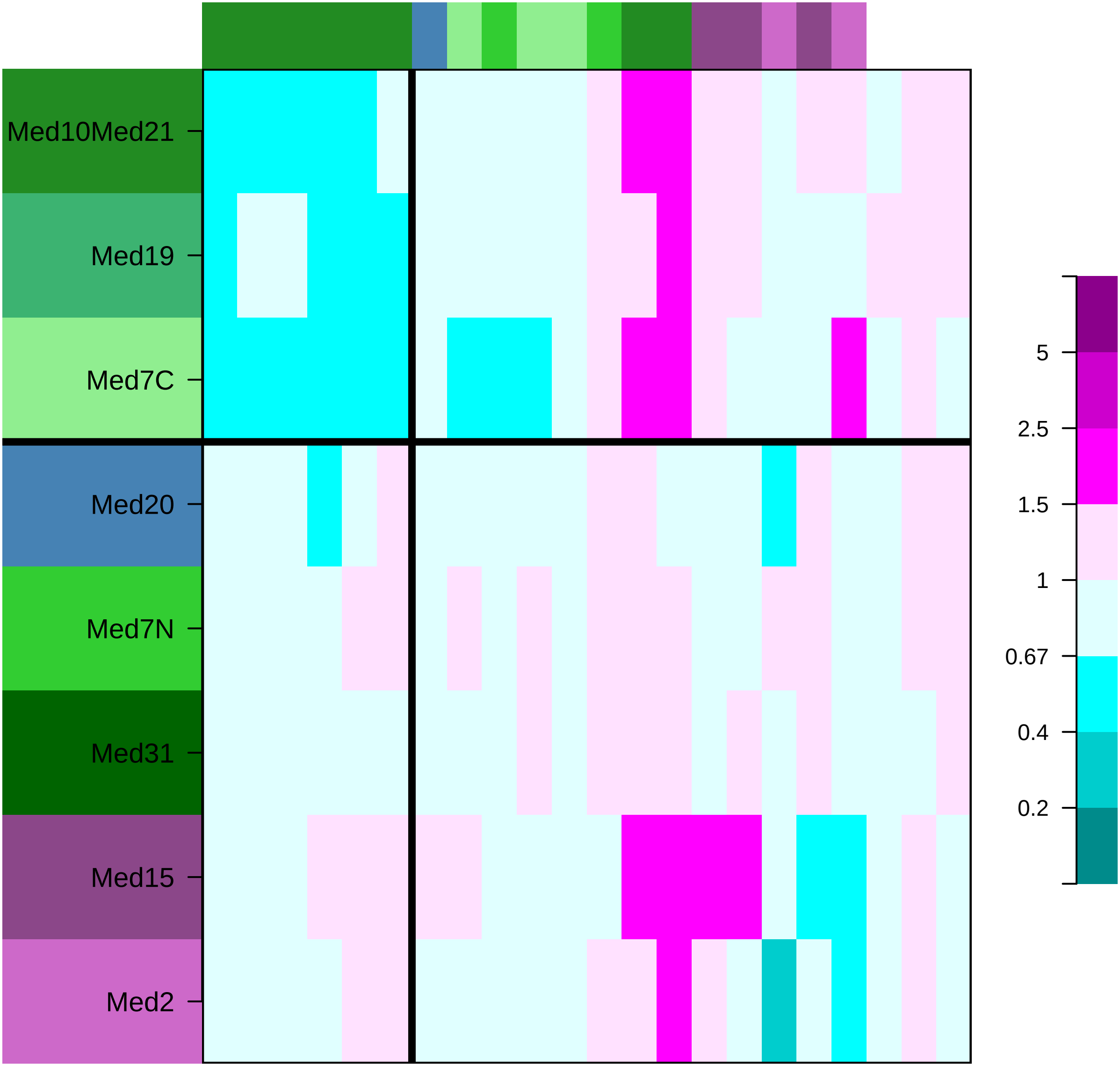

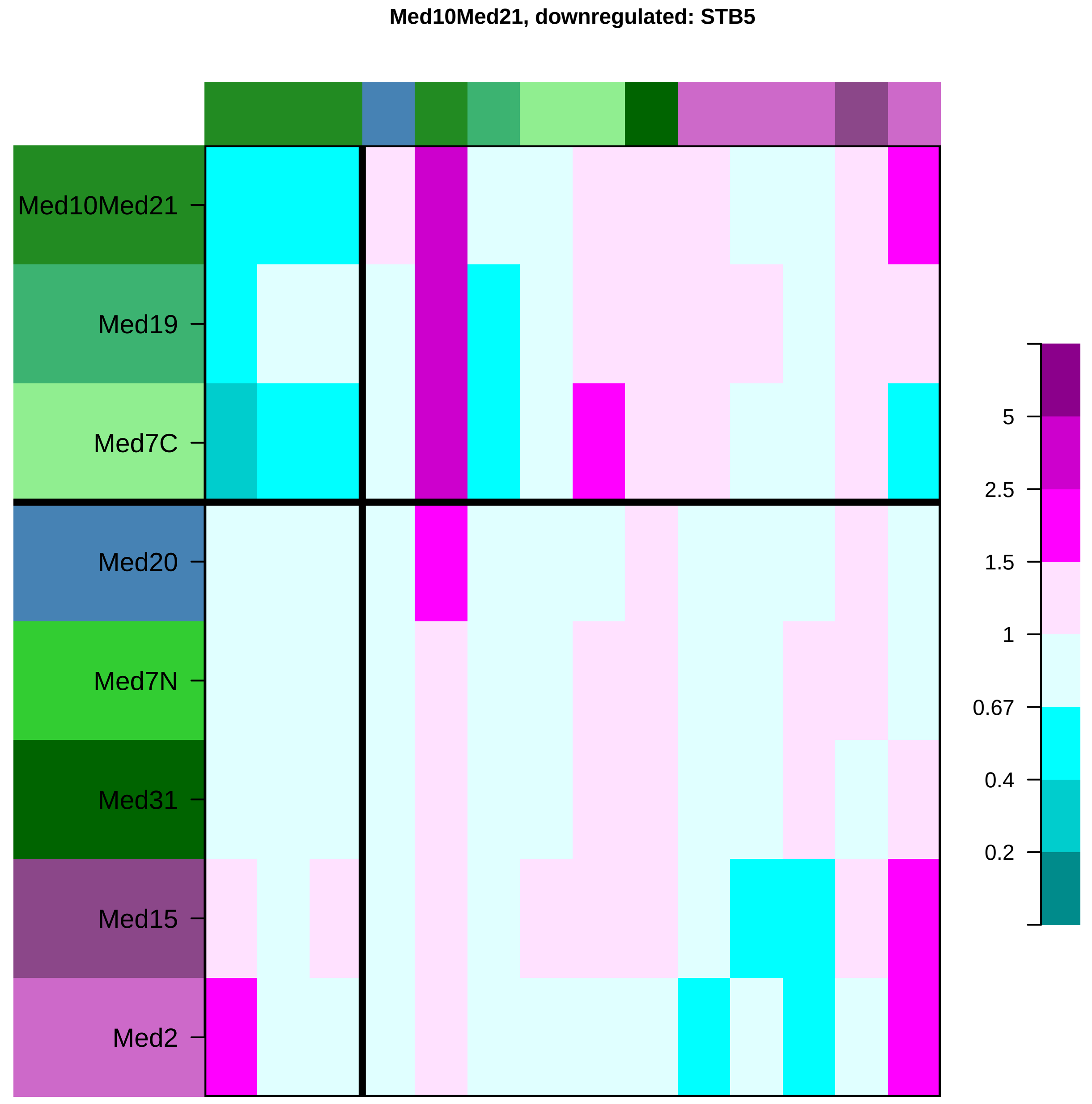

Med10Med21, downregulated: STB5
Med10Med21
Med19
Med7C
Med20
Med7N
Med31
Med15
Med2
5
2.5
1.5
1
0.67
0.4
0.2

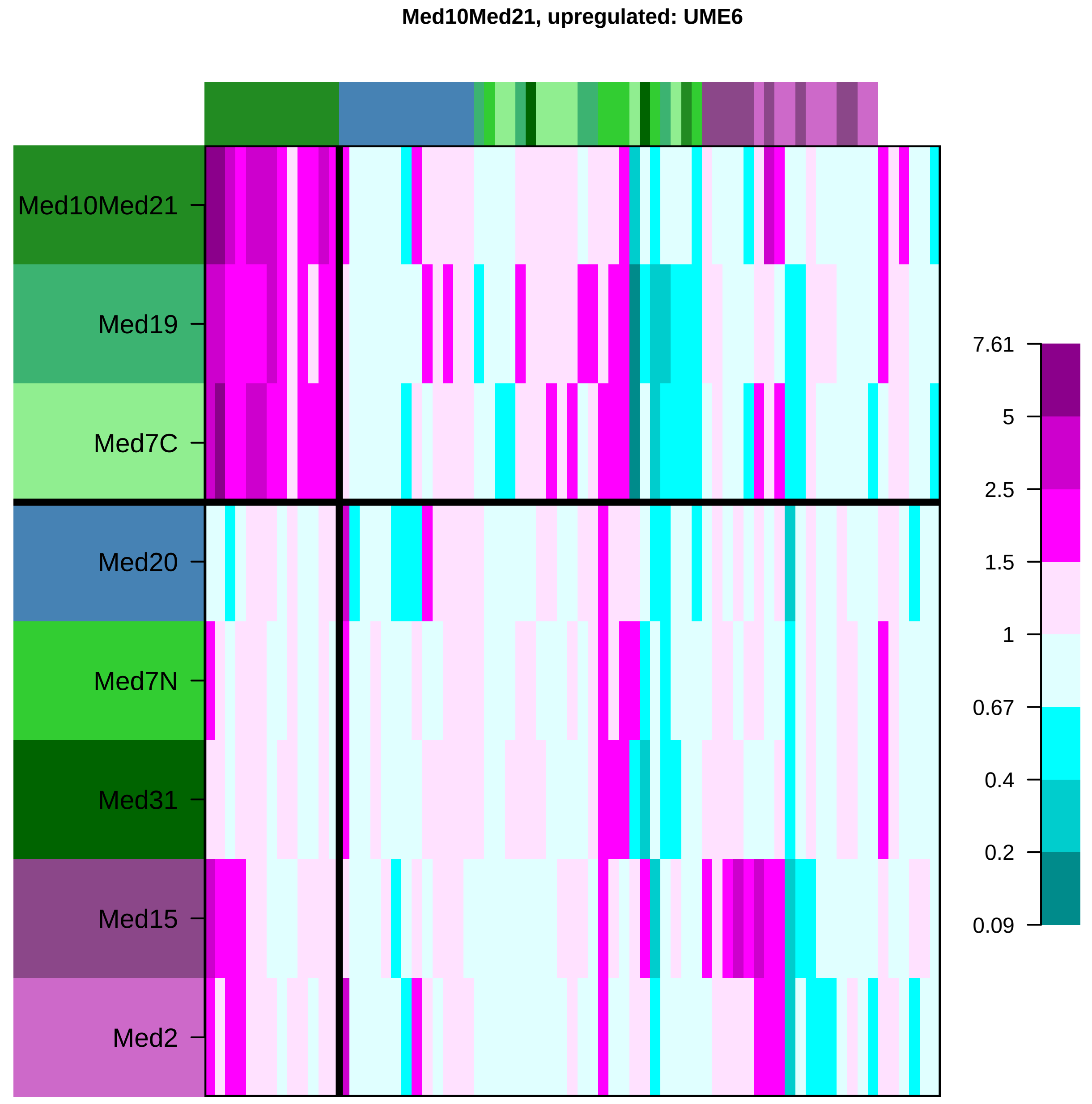
Med10Med21, upregulated: UME6
Med10Med21
Med19
Med7C
Med20
Med7N
Med31
Med15
Med2
7.61
5
2.5
1.5
1
0.67
0.4
0.2
0.09

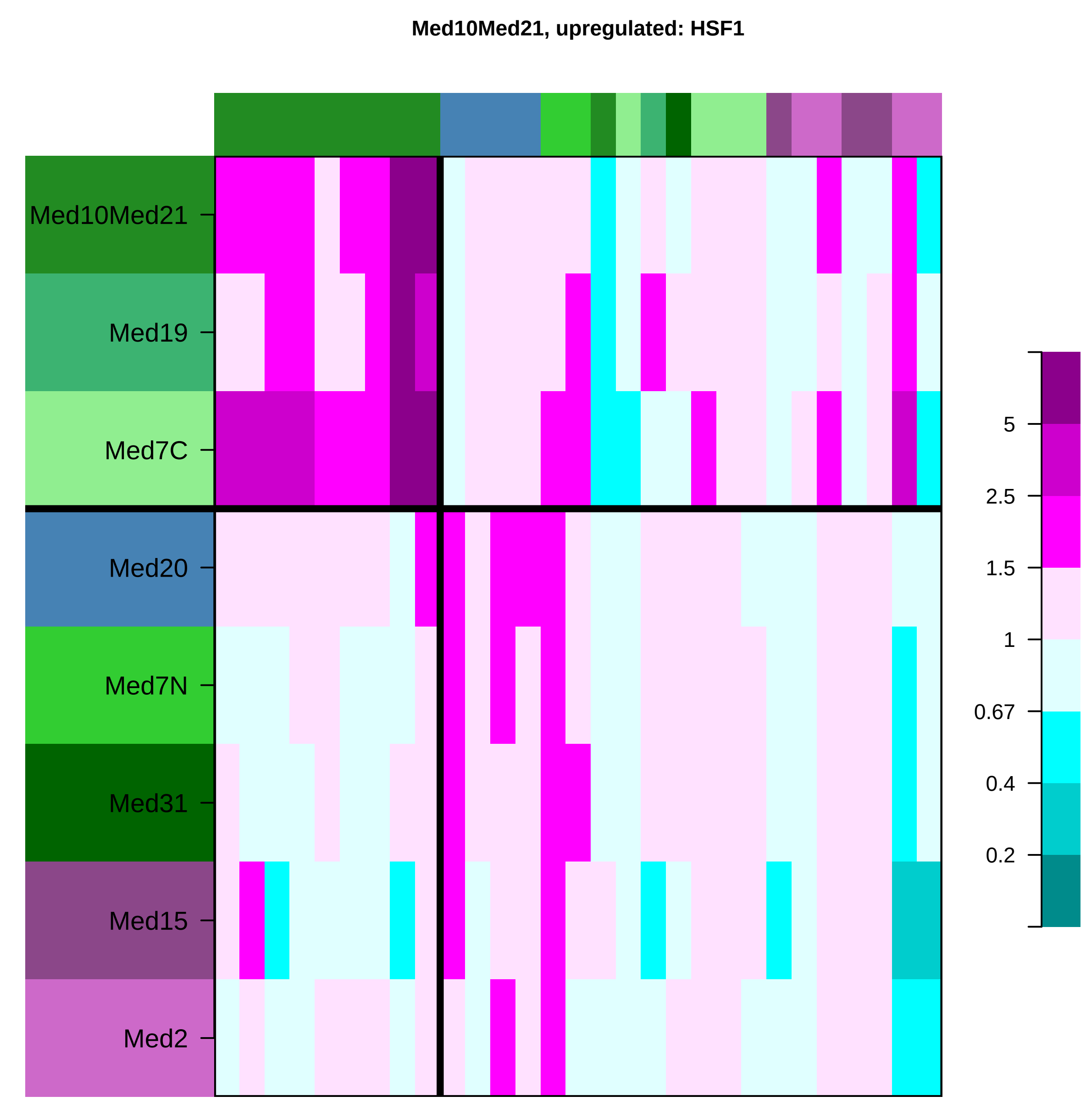

Med10Med21, upregulated: HSF1
Med10Med21
Med19
Med7C
Med20
Med7N
Med31
Med15
Med2
5
2.5
1.5
1
0.67
0.4
0.2

Med10Med21, upregulated: HAP4

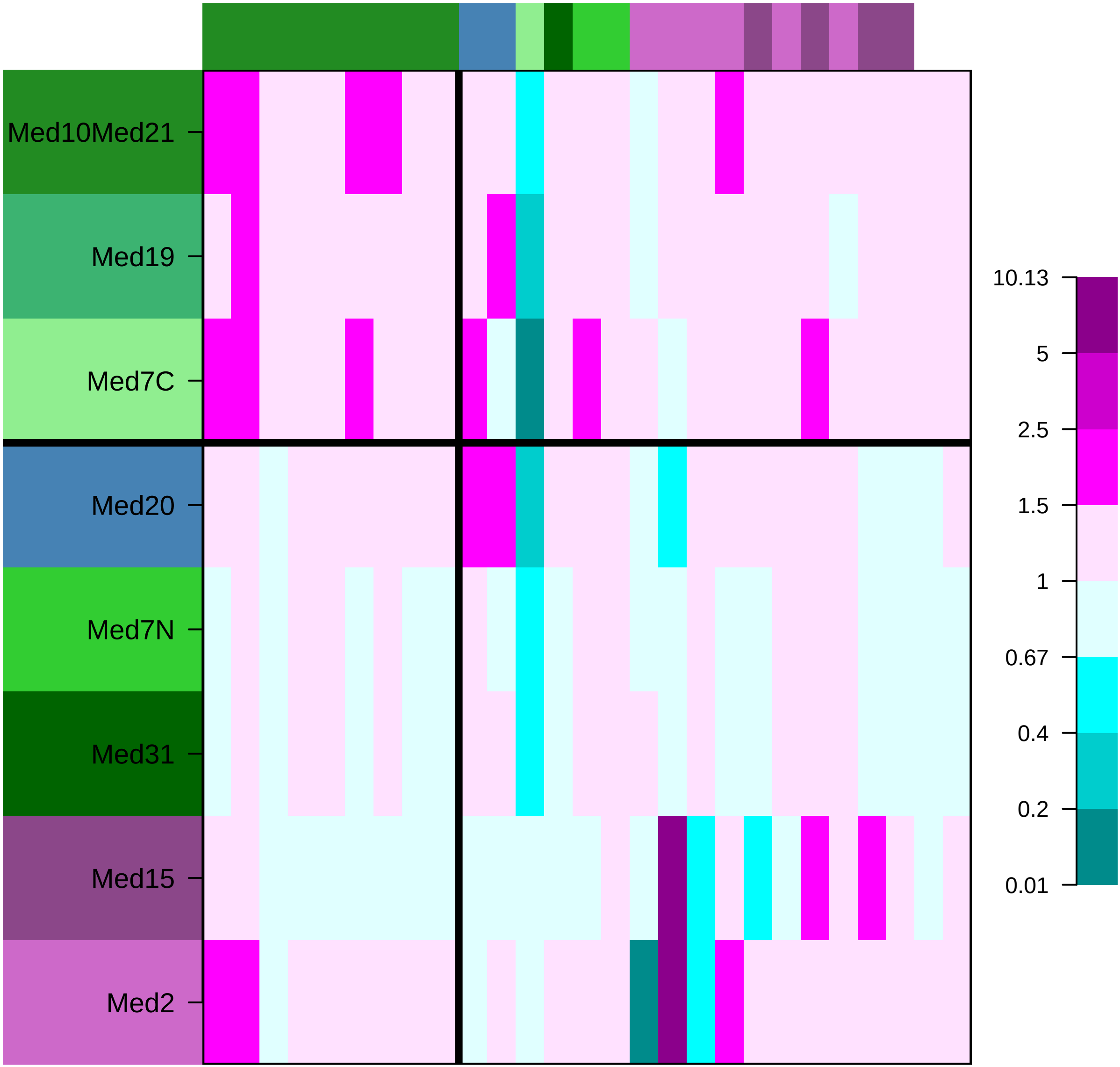

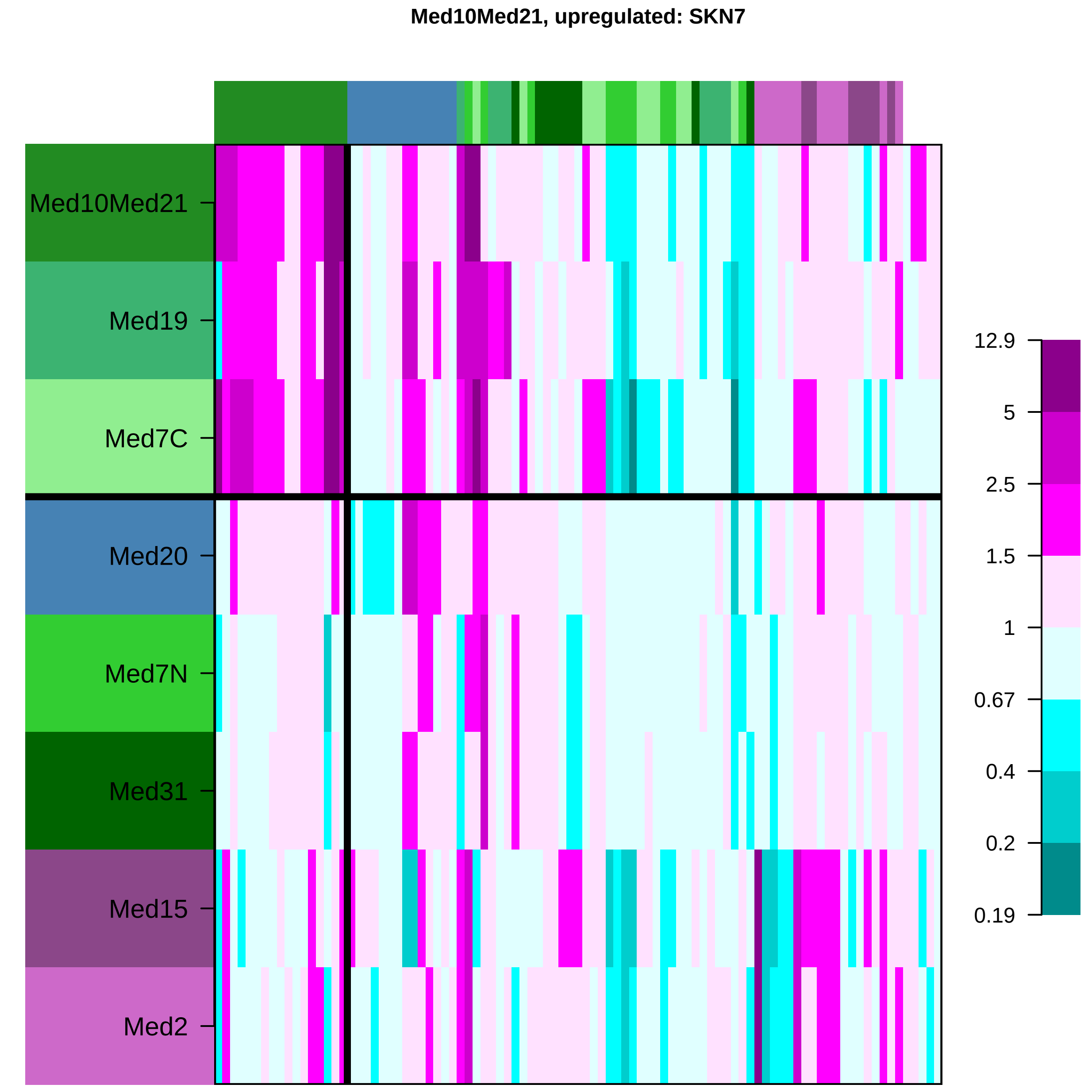
Med10Med21, upregulated: SKN7
Med10Med21
Med19
Med7C
Med20
Med7N
Med31
Med15
Med2
12.9
5
2.5
1.5
1
0.67
0.4
0.2
0.19

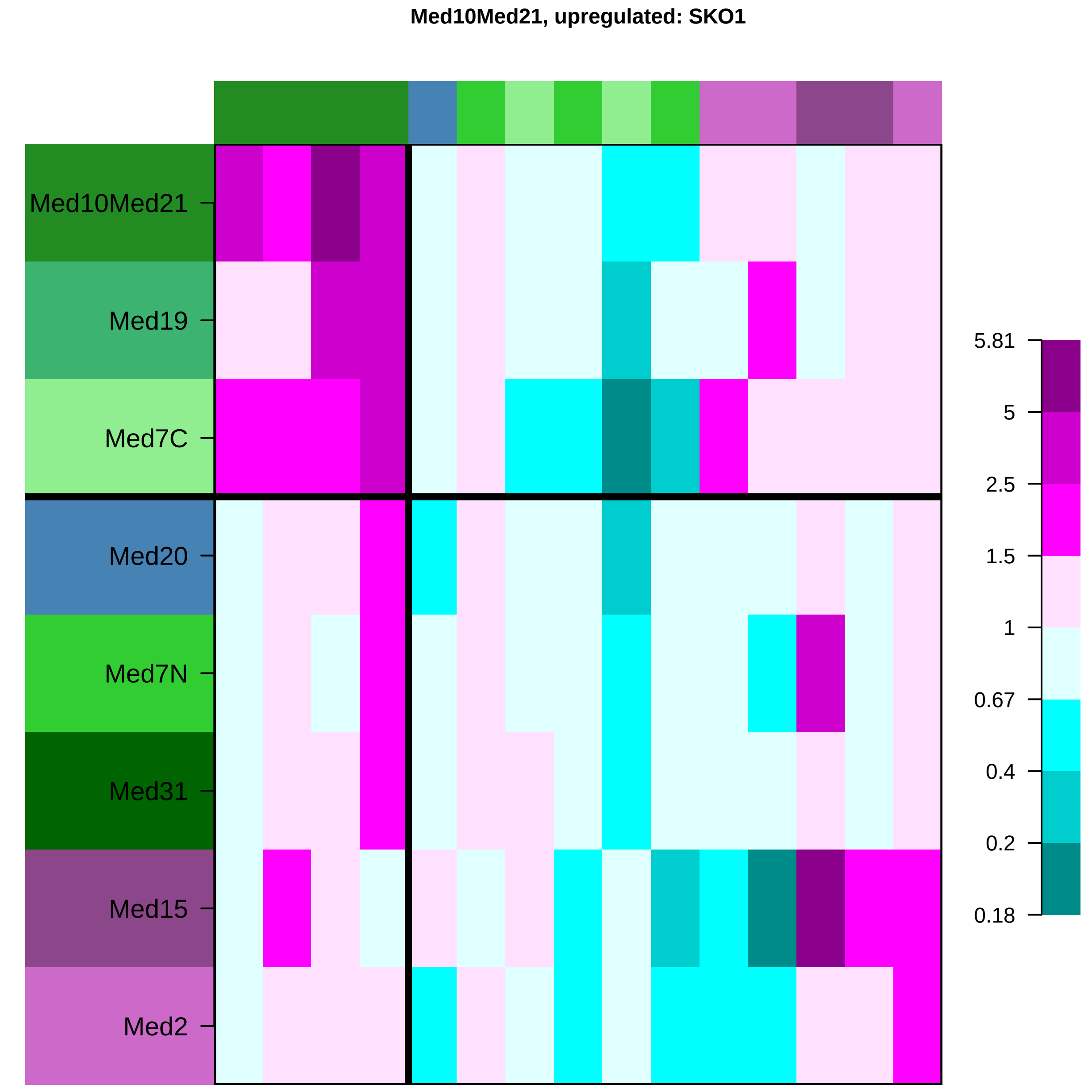

Med10Med21, upregulated: SKO1
Med10Med21
Med19
Med7C
Med20
Med7N
Med31
Med15
Med2
5.81
5
2.5
1.5
1
0.67
0.4
0.2
0.18

Med10Med21, upregulated: HAP3

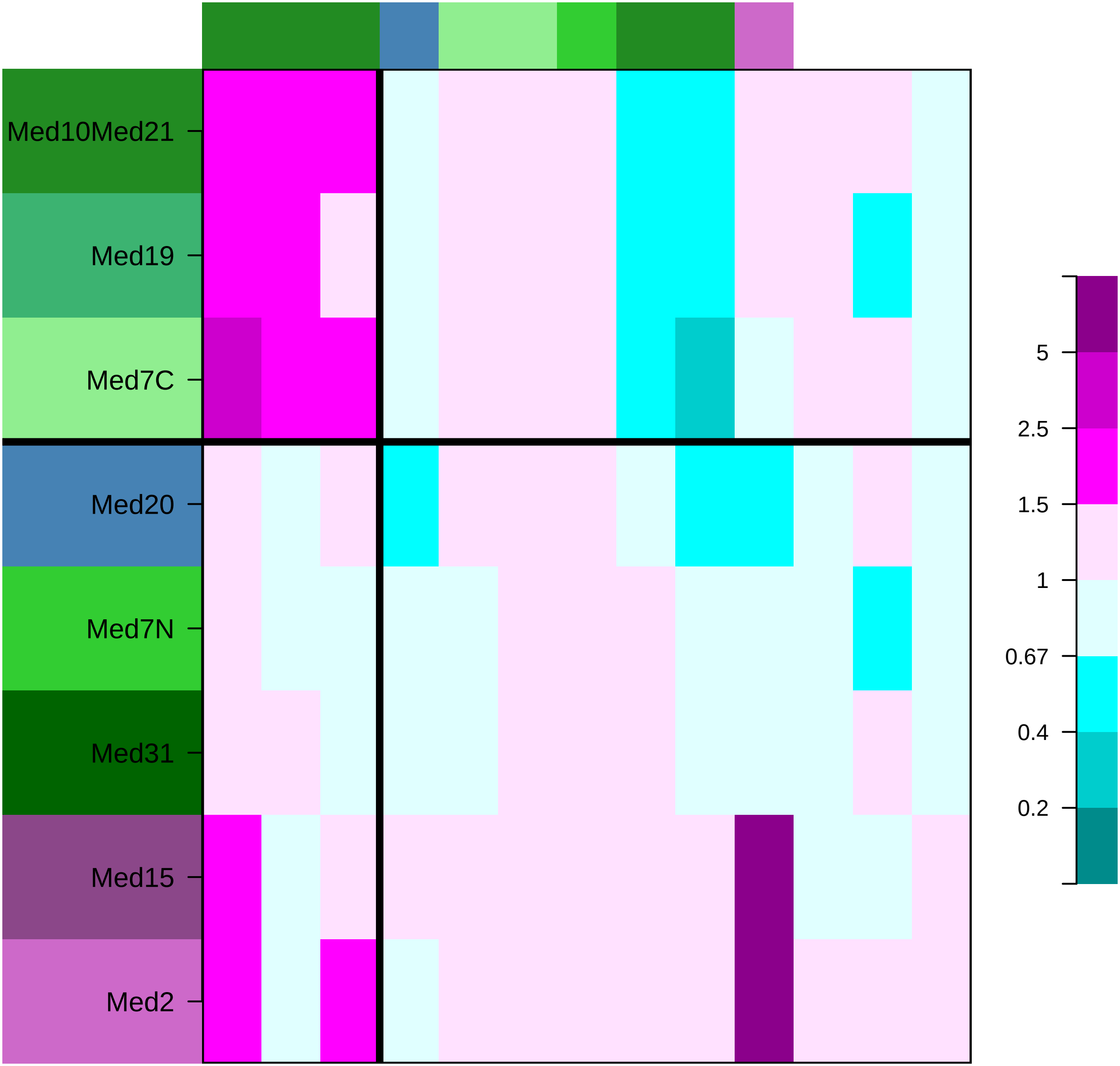

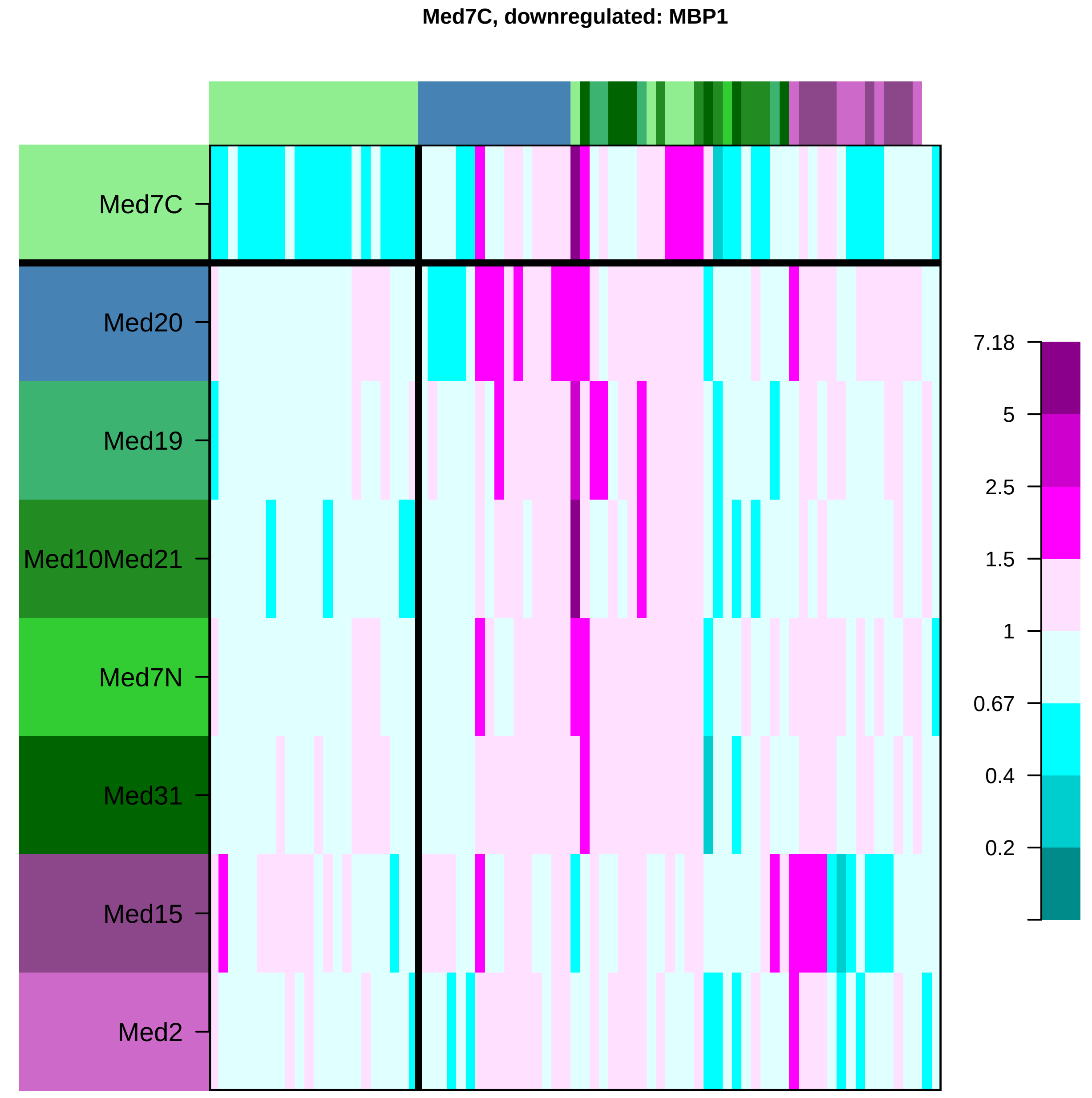
Med7C, downregulated: MBP1
Med7C
Med20
Med19
Med10Med21
Med7N
Med31
Med15
Med2
7.18
5
2.5
1.5
1
0.67
0.4
0.2

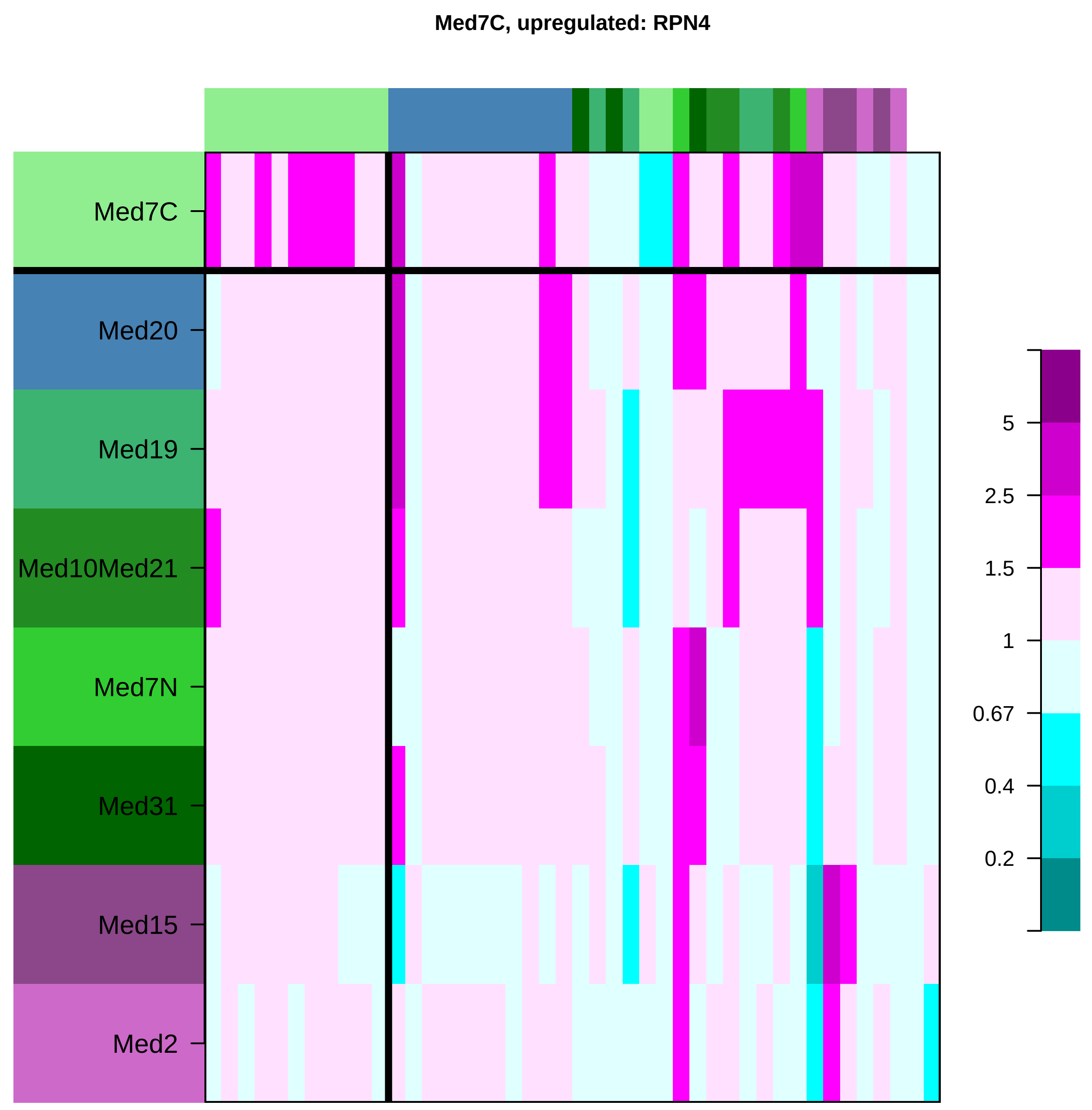

Med7C, upregulated: RPN4
Med7C
Med20
Med19
Med10Med21
Med7N
Med31
Med15
Med2
5
2.5
1.5
1
0.67
0.4
0.2

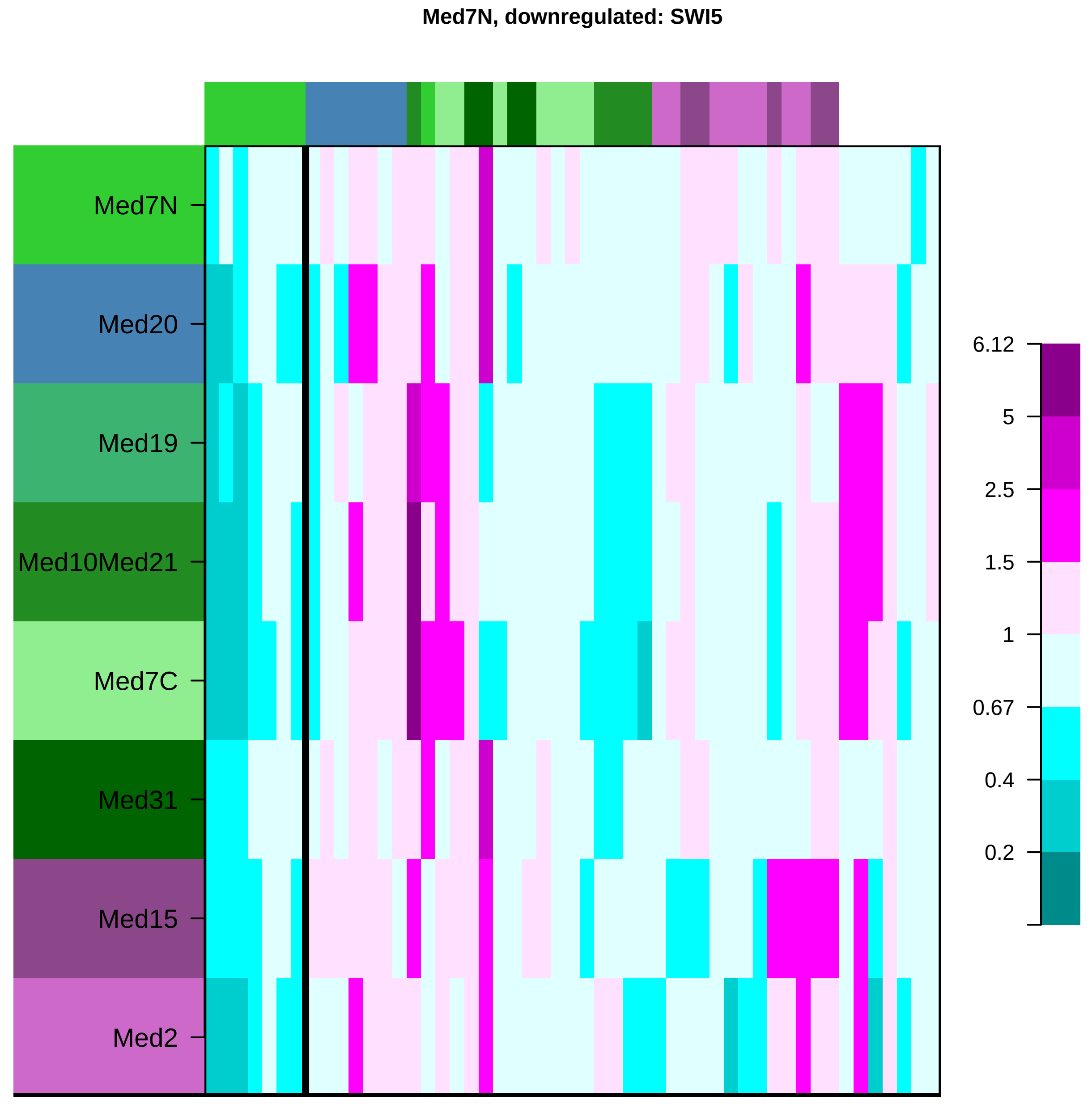

Med7N, downregulated: SWI5
Med7N
Med20
Med19
Med10Med21
Med7C
Med31
Med15
Med2
6.12
5
2.5
1.5
1
0.67
0.4
0.2

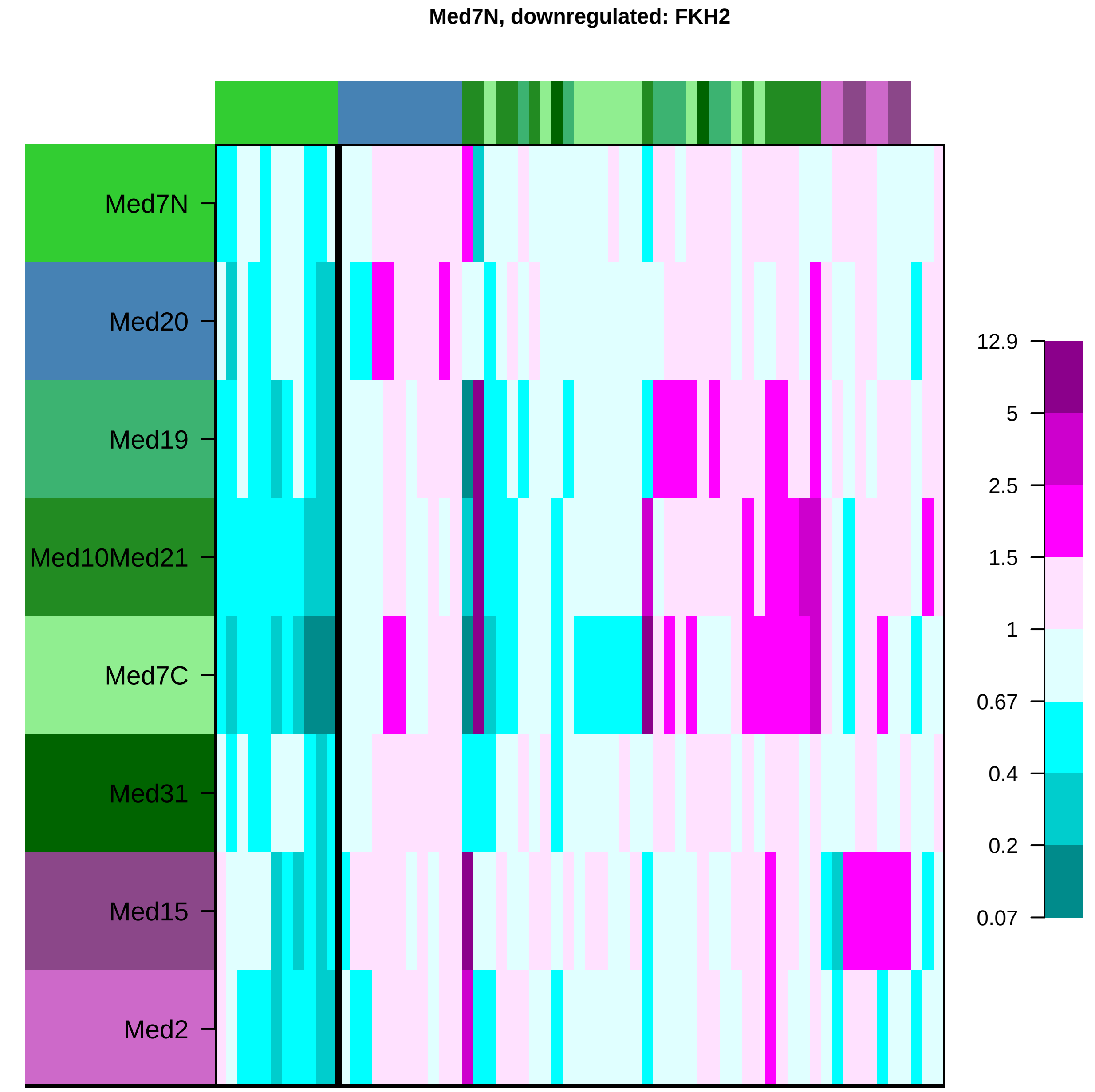

Med7N, downregulated: FKH2
Med7N
Med20
Med19
Med10Med21
Med7C
Med31
Med15
Med2
12.9
5
2.5
1.5
1
0.67
0.4
0.2
0.07

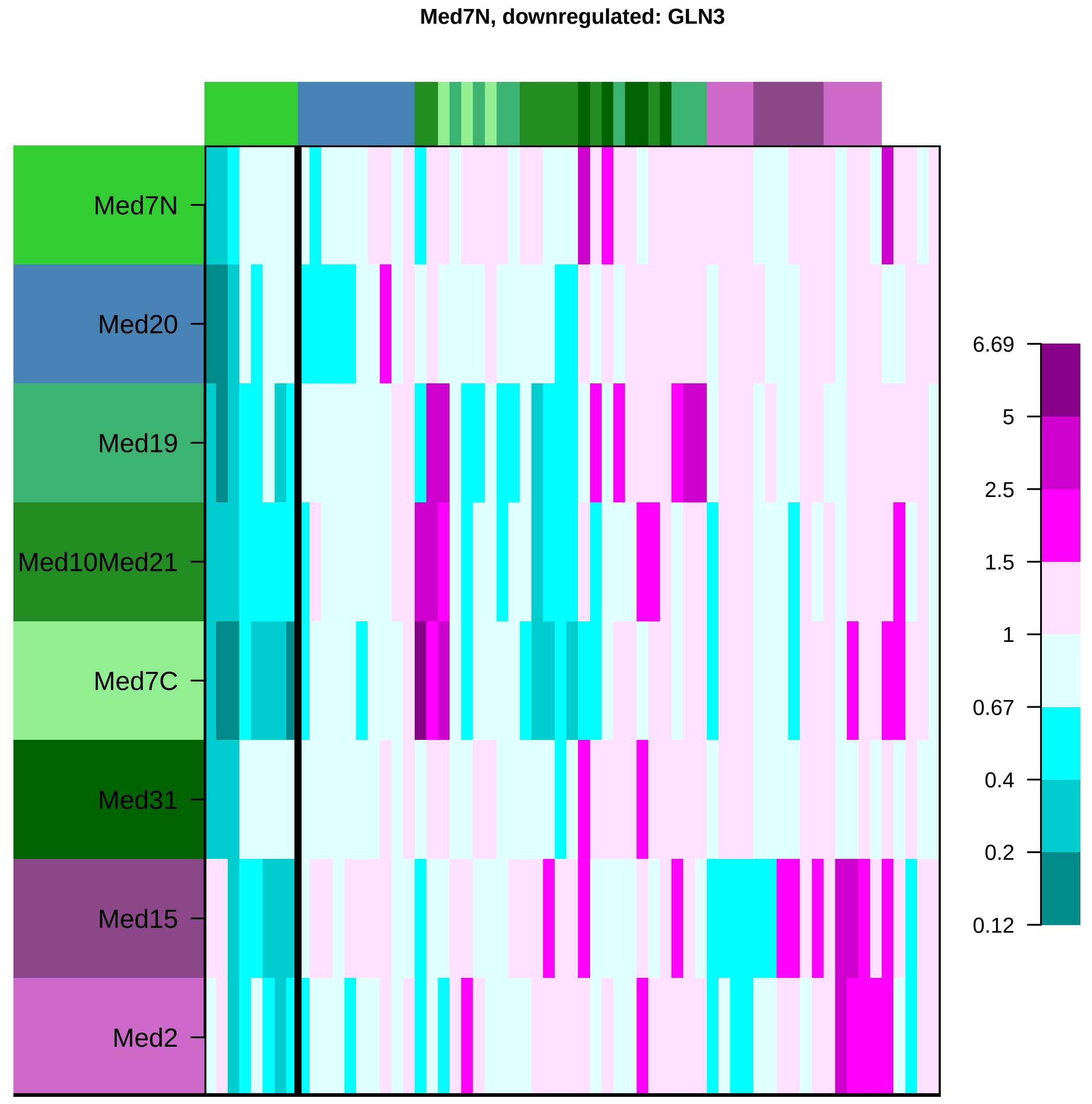

Med7N, downregulated: GLN3
Med7N
Med20
Med19
Med10Med21
Med7C
Med31
Med15
Med2
6.69
5
2.5
1.5
1
0.67
0.4
0.2
0.12

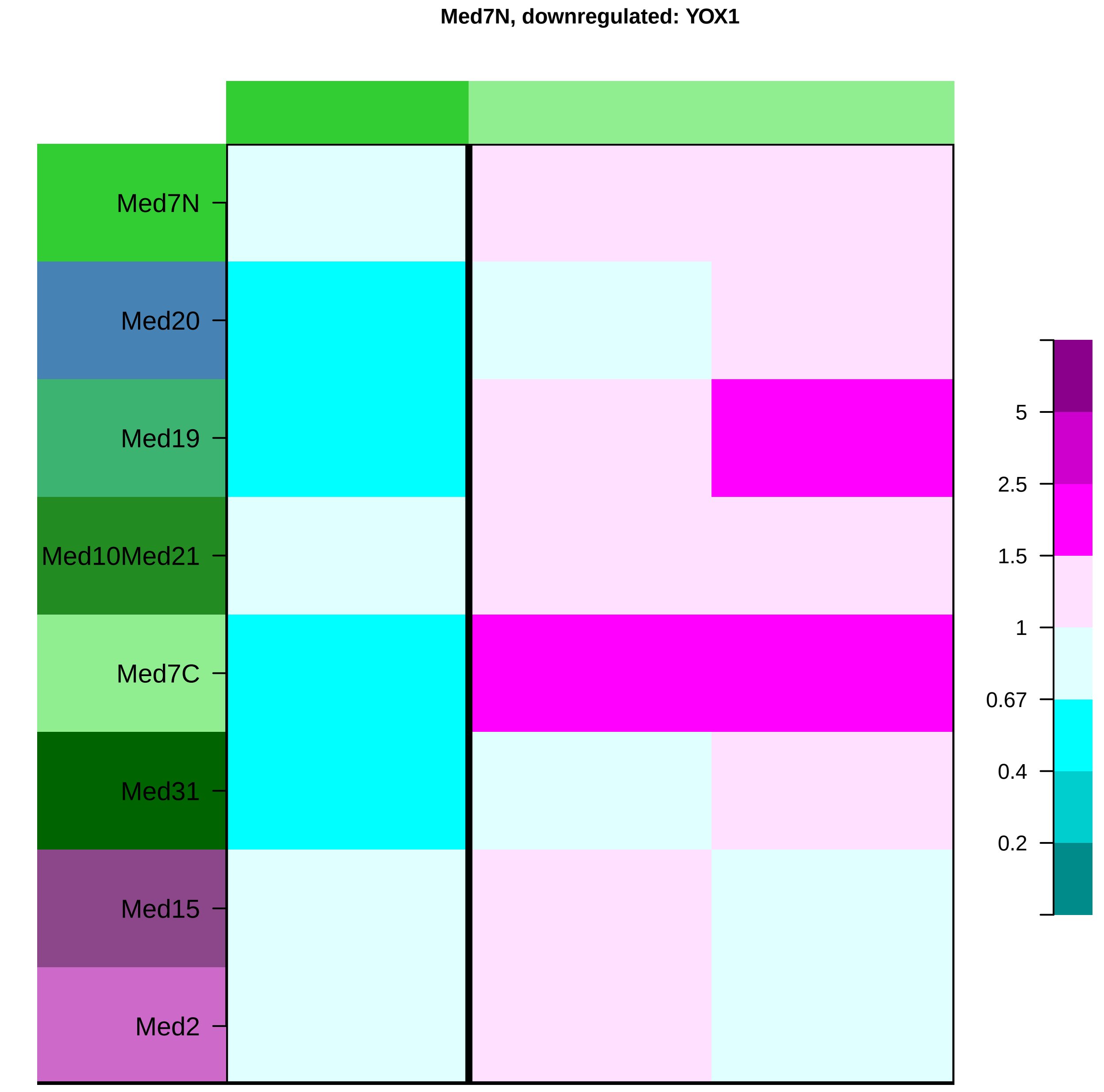
Med7N, downregulated: YOX1
Med7N
Med20
Med19
Med10Med21
Med7C
Med31
Med15
Med2
5
2.5
1.5
1
0.67
0.4
0.2

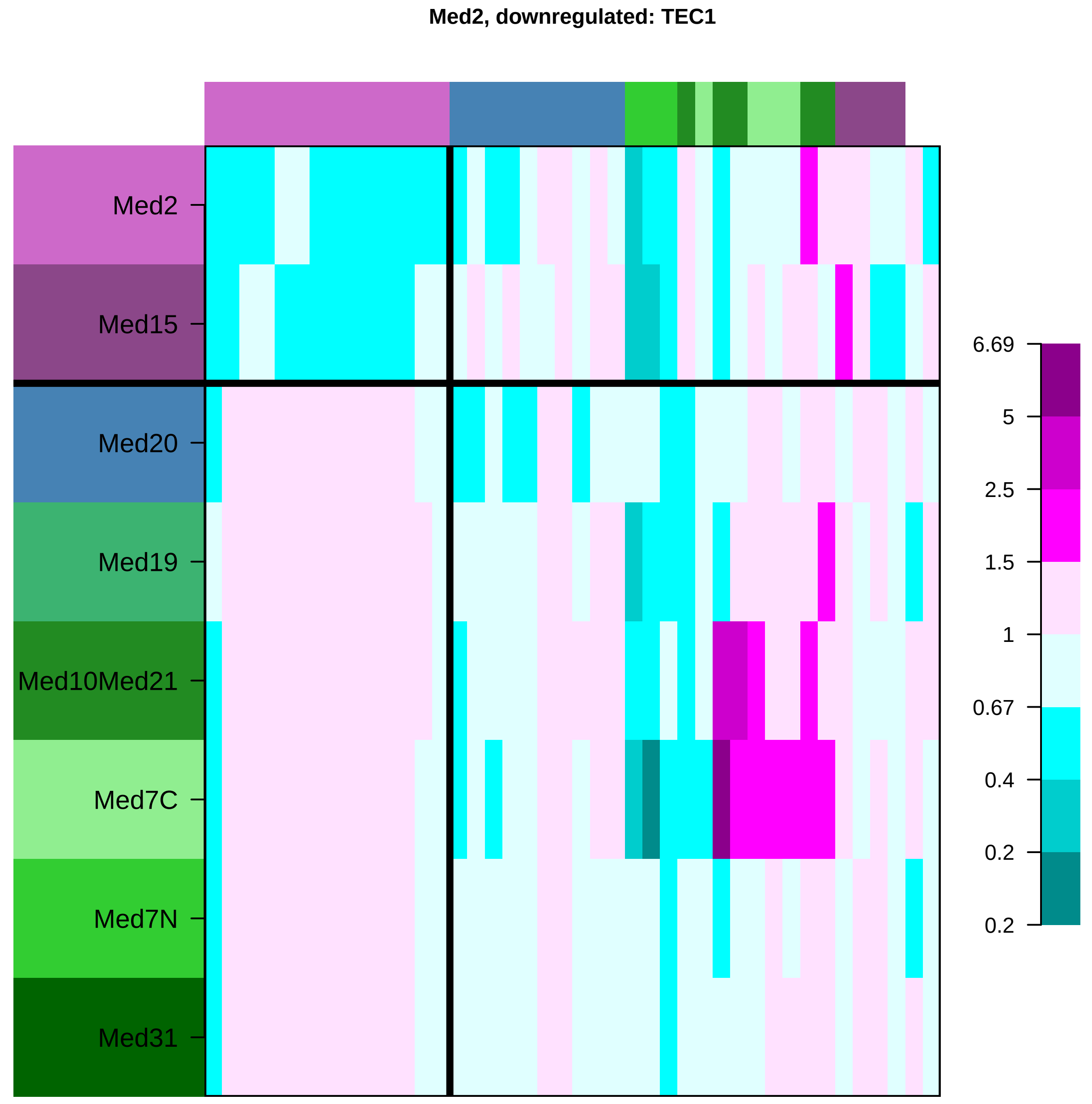

Med2, downregulated: TEC1
Med2
Med15
Med20
Med19
Med10Med21
Med7C
Med7N
Med31
6.69
5
2.5
1.5
1
0.67
0.4
0.2
0.2

Med2, downregulated: YAP6

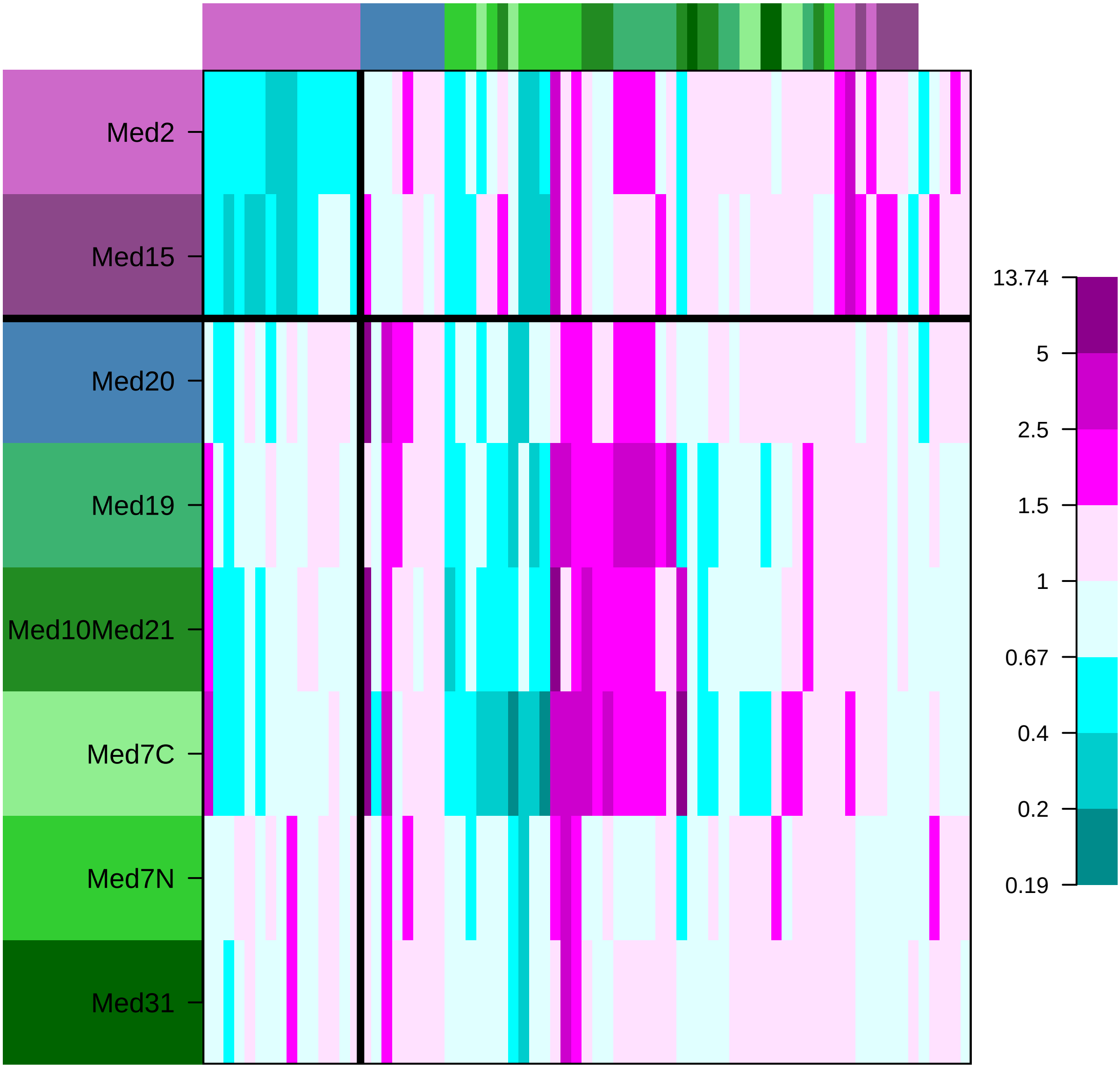

Med2, downregulated: GTS1

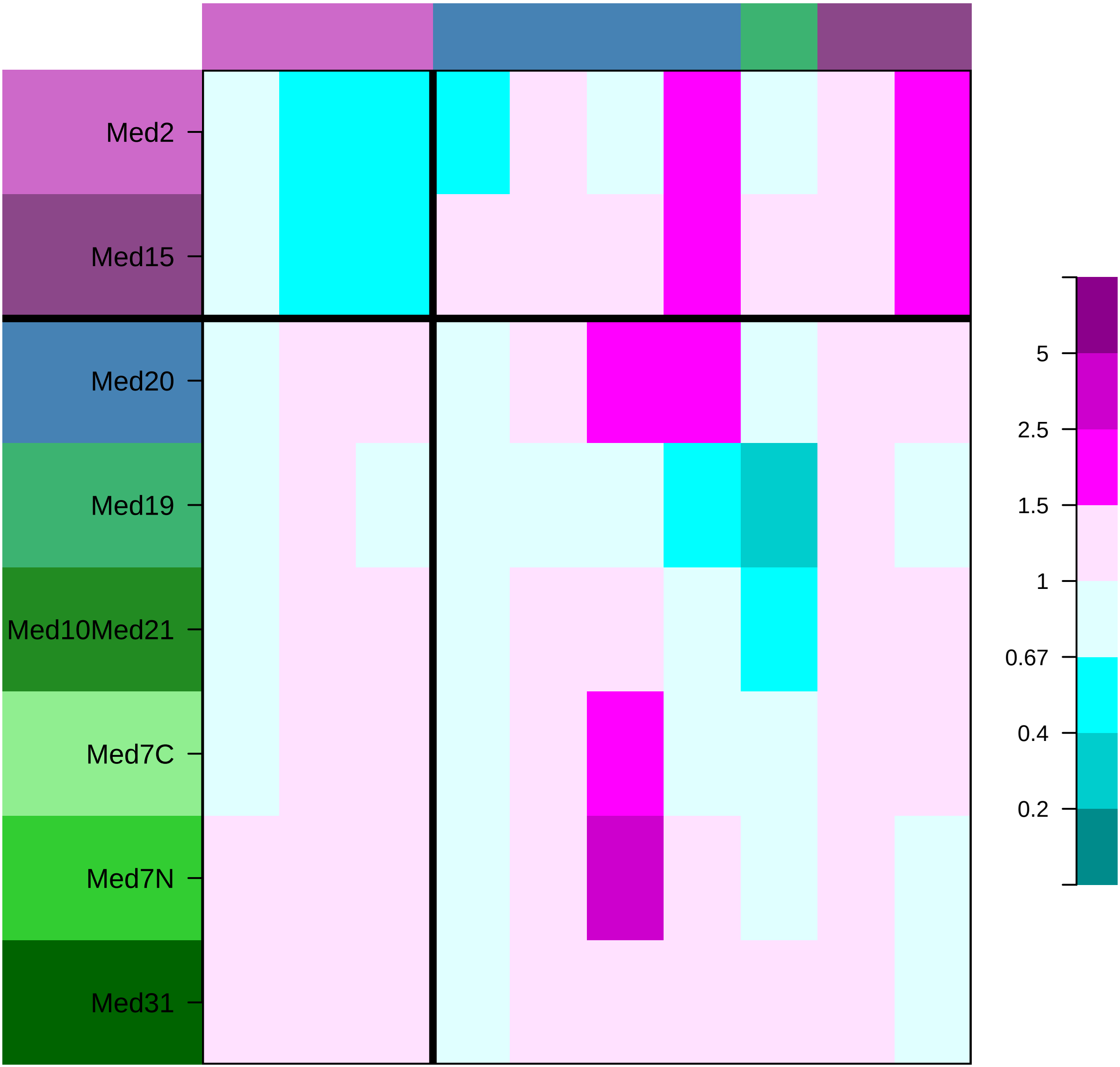

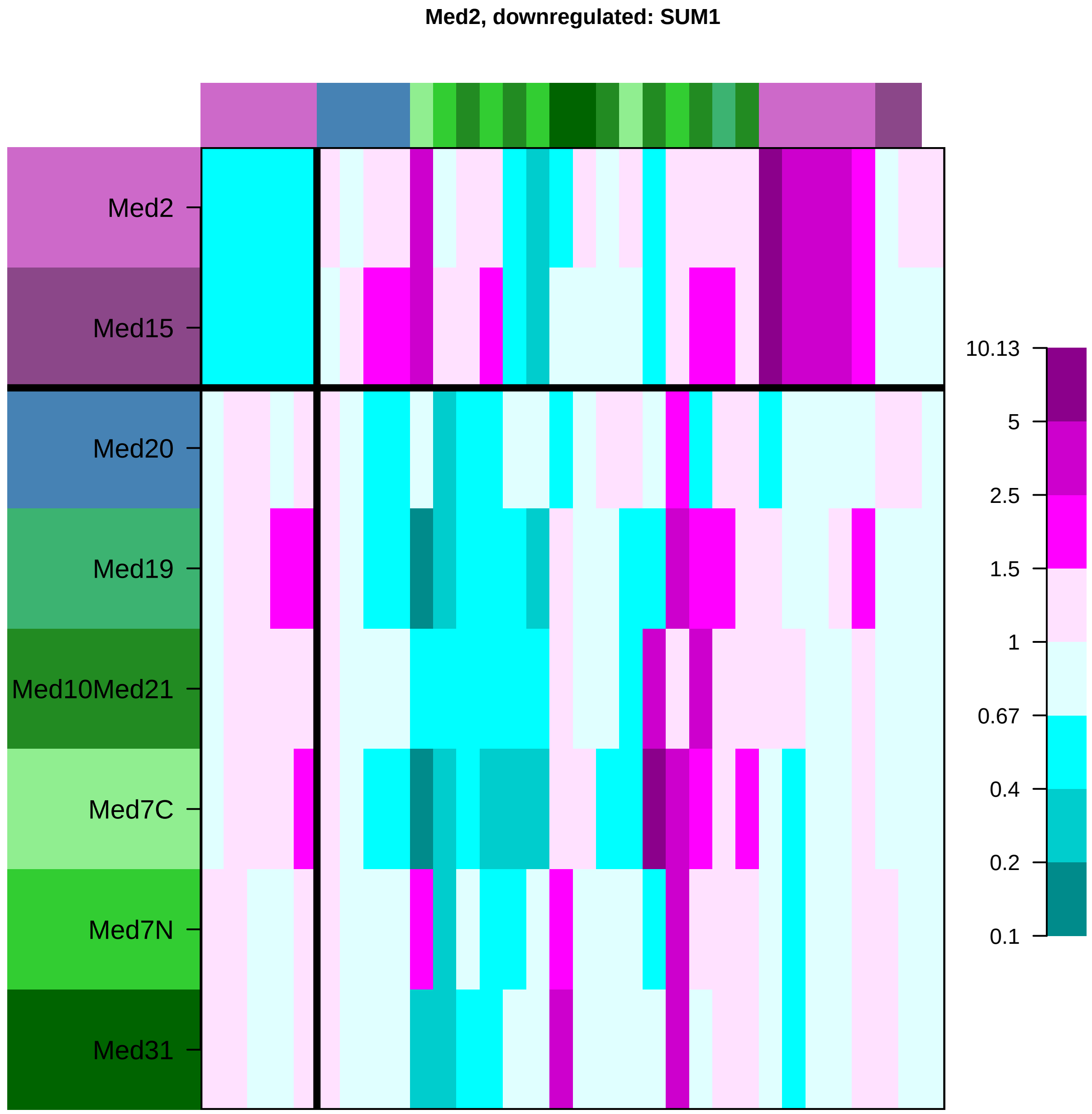

Med2, downregulated: SUM1
Med2
Med15
Med20
Med19
Med10Med21
Med7C
Med7N
Med31
10.13
5
2.5
1.5
1
0.67
0.4
0.2
0.1

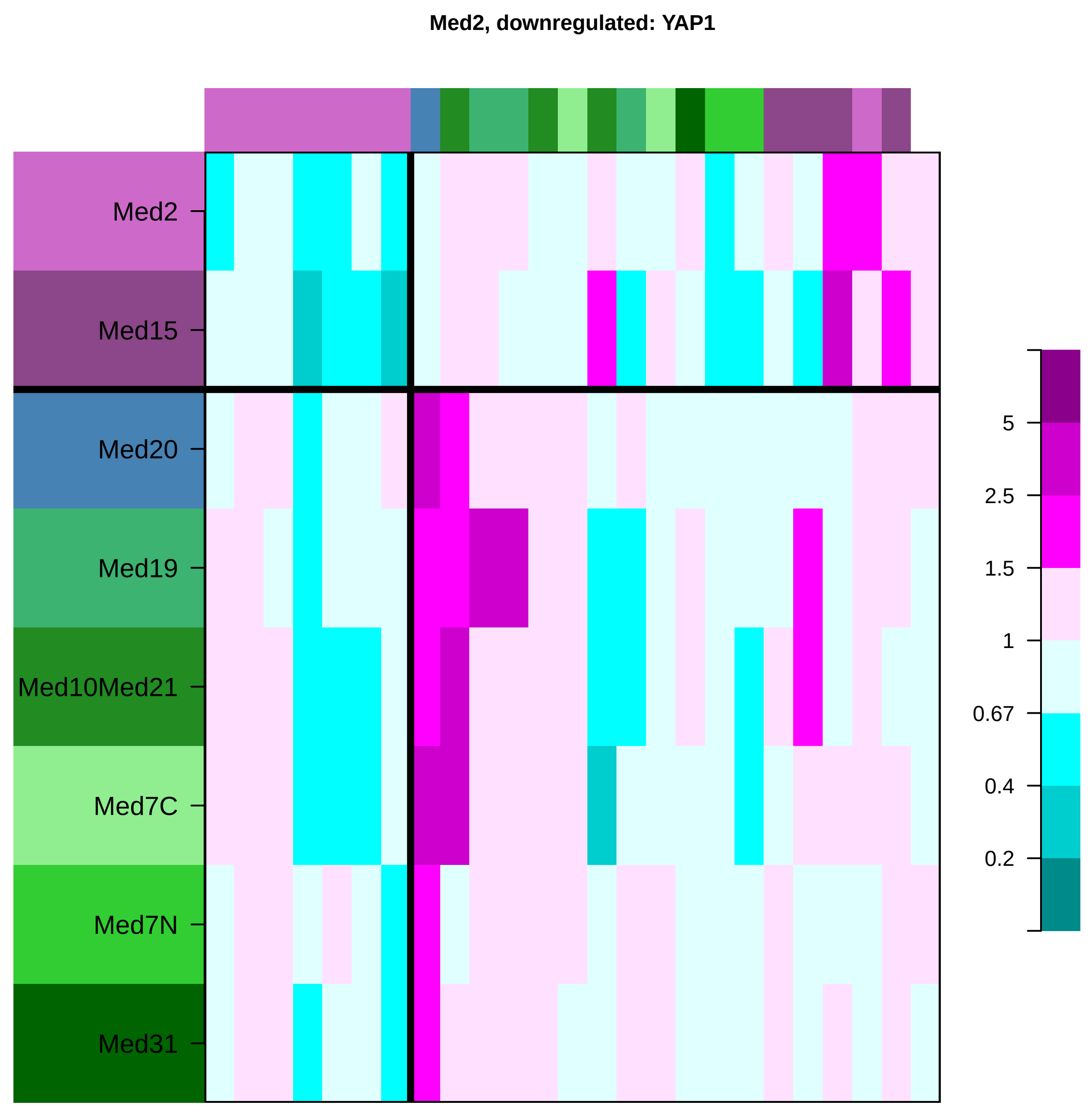
Med2, downregulated: YAP1
Med2
Med15
Med20
Med19
Med10Med21
Med7C
Med7N
Med31
5
2.5
1.5
1
0.67
0.4
0.2

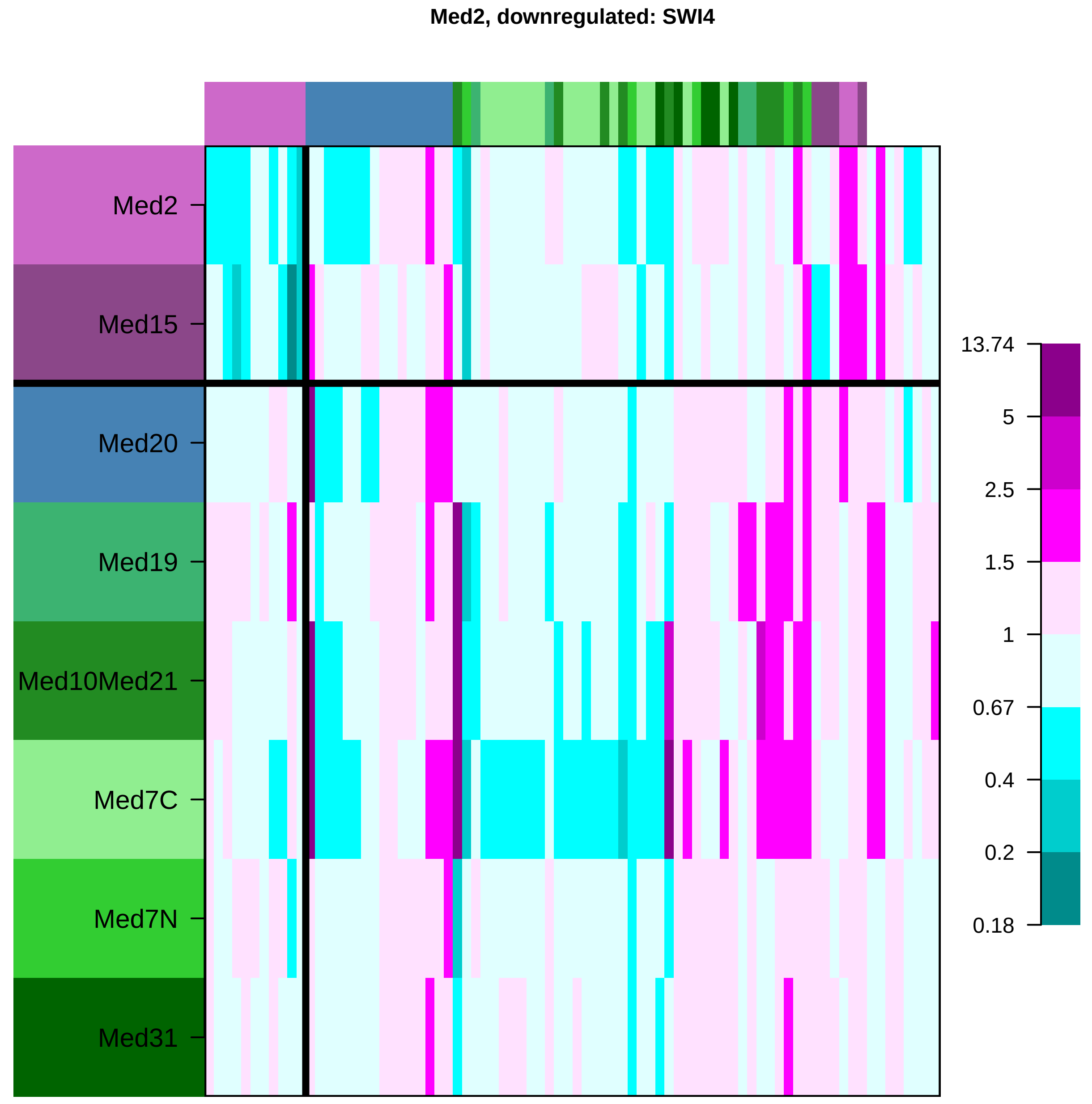
Med2, downregulated: SWI4
Med2
Med15
Med20
Med19
Med10Med21
Med7C
Med7N
Med31
13.74
5
2.5
1.5
1
0.67
0.4
0.2
0.18

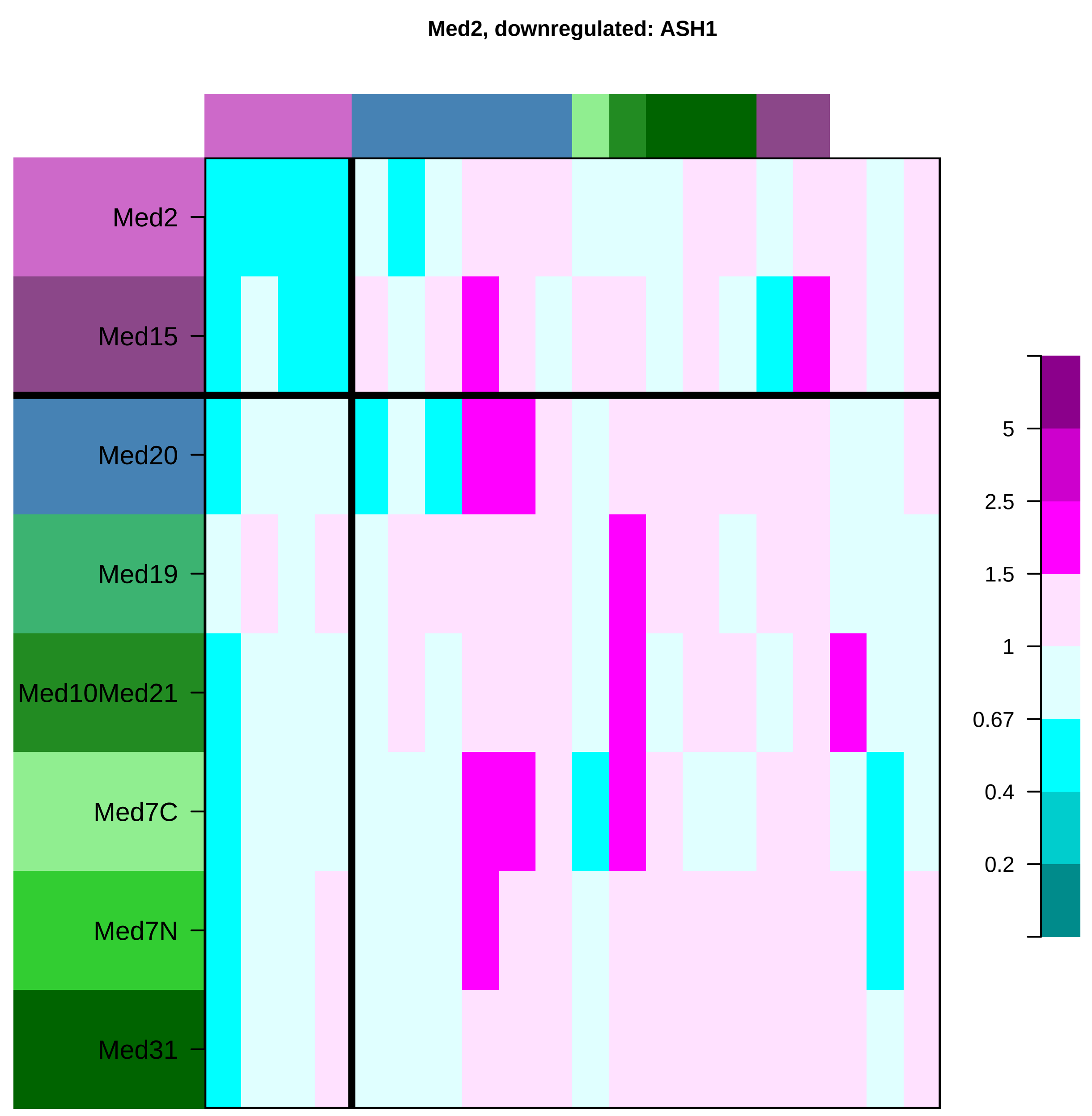
Med2, downregulated: ASH1
Med2
Med15
Med20
Med19
Med10Med21
Med7C
Med7N
Med31
5
2.5
1.5
1
0.67
0.4
0.2
