# Supplementary material for: MC EMiNEM Maps the Interaction Landscape of the Mediator
Source: PLoS Comput Biol. 2012 Jun 21;8(6):e1002568. doi: 10.1371/journal.pcbi.1002568 (PMC3380870; doi:10.1371/journal.pcbi.1002568)
Supplement: Text S1 — Additional information on methods and results. This file provides additional information on methods and results which go beyond the scope of this paper, including detailed derivations of formulas. (PDF) [file pcbi.1002568.s004.pdf]

Supplements

1. EM algorithm

This section is an extended version of the EM Section in the main text. We report the results that arise from the EM algorithm when applied to our situation. The calculations involve only elementary algebra but are sometimes tedious. According to [1], the likelihood function ("structure likelihood") of the signals graph in a NEM is

$$L(\Theta, H) = P(D \mid \Theta, H) \quad = \quad \prod_{j \in \mathcal{S}} \prod_{k \in \mathcal{E}} P(D_{jk} \mid (\Theta H)_{jk}) = \prod_{j,k} p_{jk} \tag{1.1}$$

The log likelihood can be written as

$$\begin{aligned}
\log\, L(\Theta, H) \quad &= \quad \log P(D \mid H, \Theta) = \prod_{j,k} p_{jk} \\
&= \quad \log \prod_{j,k} [P(D_{jk} \mid (\Theta H)_{jk})/q_{jk}] + \log \prod_{j,k} q_{jk} \\
&= \quad \sum_{j,k} \log \begin{cases} p_{jk}/q_{jk} & \text{if } (\Theta H)_{jk} > 0 \\ 1 & \text{if } (\Theta H)_{jk} = 0 \end{cases} + \text{const} \\
&= \quad \sum_{j,k} \begin{cases} R_{jk} & \text{if } (\Theta H)_{jk} > 0 \\ 0 & \text{if } (\Theta H)_{jk} = 0 \end{cases} + \text{const} \\
&= \quad \sum_{j,k} (\Theta H)_{jk} R_{jk} + \text{const} \\
&= \quad \sum_{j} \left[\sum_{k} (\Theta H)_{jk} (R^T)_{kj} \right] + \text{const} \\
&= \quad \sum_{j} (\Theta H R^T)_{jj} + \text{const} \qquad (1.2) \\
&= \quad \text{trace}(\Theta H R^T) + \text{const} \qquad (1.3)
\end{aligned}$$

The (full) posterior is then given by

$$\log P(\Theta, H \mid D) = \log P(D \mid H, \Theta) + \log \pi(\Theta, H) + \text{const}$$

We assume edge-wise independent priors, $\pi(\Theta, H) = \pi^{\mathcal{S}}(\Theta) \cdot \pi^{\mathcal{E}}(H)$, and $\pi^{\mathcal{S}}(\Theta) = \prod_{i,j} \pi^{\mathcal{S}}(\Theta_{ij})$, $\pi^{\mathcal{E}}(H_{\bullet k}) = \prod_k \pi^{\mathcal{E}}(H_{\bullet k})$.

1.1. The general EM algorithm.

Throughout this section, the data D resp. the matrix R is considered given and fixed. We want to find the maximum *a posteriori* estimate $\hat{\Theta}$ for the signals graph,

$$\hat{\Theta} = \arg\max_{\Theta} P(\Theta \mid D) = \arg\max_{\Theta} \sum_{H \in \mathcal{M}_{\mathcal{E}}} P(\Theta, H \mid D) \tag{1.4}$$

The Expectation-Maximization algorithm was developed exactly for this purpose, to perform a maximization task in the presence of hidden variables [2]. The EM proceeds by iteratively constructing a sequence of parameter estimates Θ^t, $t = 1, 2, ...$ such that the sequence $(P(\Theta^t \mid D))_{t=1,2,...}$ is monotonically increasing, and converges (under mild additional assumptions that are met in our case) to a local maximum of $P(\Theta \mid D)$.

The expectation (E-)step of the EM algorithm involves calculating the expectation value $Q(\Theta; \Theta^t)$,

$$Q(\Theta; \Theta^t) \;=\; \mathbb{E}_{P(H|D,\Theta^t)} \left[\log P(D, H \mid \Theta) \right] \;=\; \sum_{H \in \mathcal{M}_{\mathcal{E}}} \log P(D, H \mid \Theta) \cdot P(H \mid D, \Theta^t) \;. \tag{1.5}$$

The maximization (M-)step of the EM algorithm then consists of finding

$$\Theta^{t+1} = \arg\max_{\Theta} \left[\, Q(\Theta; \Theta^t) + \log \pi^{\mathcal{S}}(\Theta) \, \right] \ , \tag{1.6}$$

which is usually a much easier task than solving (1.4) directly. In the following, both steps are described in detail.

1.2. **The E-step.** Let us assume that the priors for Θ and H are independent, $\pi(\Theta, H) = \pi^{\mathcal{S}}(\Theta)\pi^{\mathcal{E}}(H)$. Then, the terms in $Q(\Theta; \Theta^t)$ can be rearranged

$$\begin{aligned} Q(\Theta;\Theta^t) &= \mathbb{E}_{P(H \mid D, \Theta^t)} \left[\log P(D, H \mid \Theta) \right] \\ &= \sum_H P(H \mid D, \Theta^t) \log P(D, H \mid \Theta) \\ &= \sum_H \frac{P(D \mid H, \Theta^t) P(H \mid \Theta^t)}{P(D \mid \Theta^t)} \log(P(D \mid H, \Theta) P(H \mid \Theta)) \\ &\overset{\pi(H,\Theta) = \pi^{\mathcal{E}}(H)\pi^{\mathcal{S}}(\Theta)}{=} \frac{1}{P(D \mid \Theta^t)} \sum_H P(D \mid H, \Theta^t) \pi^{\mathcal{E}}(H) [\log P(D \mid H, \Theta) + \log \pi^{\mathcal{E}}(H)] \\ &= c^{-1} \sum_H P(D \mid H, \Theta^t) \pi^{\mathcal{E}}(H) \log P(D \mid H, \Theta) \ + \ \text{const} \end{aligned} \tag{1.7}$$

with a normalizing factor $c = P(D \mid \Theta^t) = \sum_H P(D \mid H, \Theta^t)\pi^{\mathcal{E}}(H)$ and a constant that does not depend on Θ. The problem of maximizing $Q(\Theta; \Theta^t)$ is therefore equivalent to maximizing $\tilde{Q}(\Theta; \Theta^t)$, where

$$\tilde{Q}(\Theta; \Theta^t) = c^{-1} \sum_H P(D \mid H, \Theta^t) \pi^{\mathcal{E}}(H) \log P(D \mid H, \Theta) \tag{1.8}$$

We seek for an expression for (1.6) which is amenable to analytic maximization strategies. Let $V = \mathbb{R}^{\mathcal{E}}$ be an m-dimensional vector space, which is spanned by the unit column vectors $e_k \in V$, $k \in \mathcal{E}$, and let $e_0 = 0 \in V$. We assume further that the prior for H factorizes into priors for each effect,

$$\pi^{\mathcal{E}}(H) = \prod_{k \in \mathcal{E}} \pi_k^{\mathcal{E}}(He_k) \ . \tag{1.9}$$

Let d_j be the j-th unit column vector of dimension n, and d_0 the n-dimensional null vector. The NEM model assumes that each effect assigns to at most one signal, so $\pi_k^{\mathcal{E}}(v) = 0$ for each vector $v \notin \{d_j, j = 0, ..., n\}$, $k \in \mathcal{E}$, and

$$\pi_k^{\mathcal{E}}(d_j) = \pi_{jk} \ , \ j = 0, 1, ..., n, \ \text{and} \ \sum_{j=0}^{n} \pi_{jk}^{\mathcal{E}} = 1 \ . \tag{1.10}$$

The $m \times m$ unit matrix is denoted by E. Be aware of the identity $E = \sum_{k \in \mathcal{E}} e_k e_k^T$. We take advantage of the fact that the trace of a quadratic matrix is a linear function, and that $tr(AB) = tr(BA)$ for arbitrary (compatible) matrices A, B.

$$\begin{aligned} tr(\Theta H R^T) &= tr(R^T \Theta H) \ = \ tr(\sum_{k \in \mathcal{E}} e_k e_k^T R^T \Theta H) \\ &= \sum_{k \in \mathcal{E}} tr(e_k e_k^T R^T \Theta H) \ = \ \sum_{k \in \mathcal{E}} e_k^T R^T \Theta (He_k) \end{aligned} \tag{1.11}$$

Thus by (1.3), letting $g_k(v, \Theta) = e_k^T R^T \Theta v$,

$$\log P(D \mid H, \Theta) \ = \ \sum_{k \in \mathcal{E}} g_k(He_k, \Theta) \ + \ \text{const.} \tag{1.12}$$

Analogously,

$$P(D \mid H, \Theta^t) \;\propto\; \exp(tr(\Theta^t H R^T)) \;=\; \prod_{k \in \mathcal{E}} f_k(He_k, \Theta^t)\;, \tag{1.13}$$

with $f_k(v, \Theta^t) = \exp(g_k(v, \Theta^t))$. For convenience we suppress the dependence of g_k on Θ (and write $g_k(v)$ instead of $g_k(v, \Theta)$) and of f_k on Θ^t (and write $f_k(v)$ instead of $f_k(v, \Theta^t)$). Let $W = \{0, 1\}^n$. The evaluation of $\tilde{Q}(\Theta; \Theta^t)$ can be simplified considerably. For $r = 1, ..., m$, let

$$F_r(\Theta) \;=\; \sum_{v_r \in W} \sum_{v_{r+1} \in W} \cdots \sum_{v_m \in W} \left(\prod_{l \geq r}^{m} \pi_l^{\mathcal{E}}(v_l) f_l(v_l) \right) \cdot \left(\sum_{k \geq r}^{m} g_k(v_k) \right) \tag{1.14}$$

Note that

$$\begin{aligned} \tilde{Q}(\Theta; \Theta^t) \quad &= \quad c^{-1} \sum_H P(D \mid H, \Theta^t) \pi^{\mathcal{E}}(H) \log P(D \mid H, \Theta) \\ &\overset{(1.12,1.13)}{=} \quad c^{-1} \sum_H \left(\prod_{l=0}^{m} \pi_l^{\mathcal{E}}(He_l) f_l(He_l) \right) \cdot \left(\sum_{k=0}^{m} g_k(He_k) \right) \\ &= \quad c^{-1}\, F_1(\Theta) \end{aligned} \tag{1.15}$$

We introduce two more terms,

$$A_k \;=\; \sum_{v \in W} \pi_k^{\mathcal{E}}(v) f_k(v) = \sum_{j=0}^{n} \pi_{jk}^{\mathcal{E}} f_k(d_j) \tag{1.16}$$

$$B_k(\Theta) \;=\; \sum_{v \in W} \pi_k^{\mathcal{E}}(v) f_k(v) g_k(v) = \sum_{j=0}^{n} \pi_{jk}^{\mathcal{E}} f_k(d_j) g_k(d_j)\;. \tag{1.17}$$

F_r can be calculated from F_{r+1} via the recursive formula (1.18):

$$\begin{aligned} F_r(\Theta) \;&=\; \sum_{v_{r+1},\dots,v_m \in W} \sum_{v_r \in W} \left(\pi_r^{\mathcal{E}}(v_r) f_r(v_r) \cdot \prod_{l>r}^{m} \pi_l^{\mathcal{E}}(v_l) f_l(v_l) \right) \cdot \left(\sum_{k \geq r}^{m} g_k(v_k) \right) \\ &=\; \sum_{v_{r+1},\dots,v_m \in W} \left(\prod_{l>r}^{m} \pi_l^{\mathcal{E}}(v_l) f_l(v_l) \right) \cdot \sum_{v_r \in W} \pi_r^{\mathcal{E}}(v_r) f_r(v_r) \cdot \left(\sum_{k \geq r}^{m} g_k(v_k) \right) \\ &=\; \sum_{v_{r+1},\dots,v_m \in W} \left(\prod_{l>r}^{m} \pi_l^{\mathcal{E}}(v_l) f_l(v_l) \right) \cdot \sum_{v_r \in W} \pi_r^{\mathcal{E}}(v_r) f_r(v_r) \cdot \left(g_r(v_r) + \sum_{k>r}^{m} g_k(v_k) \right) \\ &=\; \sum_{v_{r+1},\dots,v_m \in W} \left(\prod_{l>r}^{m} \pi_l^{\mathcal{E}}(v_l) f_l(v_l) \right) \cdot \left(B_r(\Theta) + A_r \sum_{k>r}^{m} g_k(v_k) \right) \\ &=\; B_r(\Theta) \left(\prod_{l>r}^{m} \sum_{v_l \in W} \pi_l^{\mathcal{E}}(v_l) f_l(v_l) \right) + \sum_{v_{r+1},\dots,v_m \in W} \left(\prod_{l \geq r+1}^{m} \pi_l^{\mathcal{E}}(v_l) f_l(v_l) \right) \left(A_r \sum_{k \geq r+1}^{m} g_k(v_k) \right) \\ &=\; B_r(\Theta) \prod_{l>r}^{m} A_l \;+\; A_r \cdot F_{r+1}(\Theta) \qquad (1.18) \end{aligned}$$

By reverse induction we prove the formula

$$F_r = \left(\prod_{l \geq r} A_l \right) \left(\sum_{k \geq r} \frac{B_k(\Theta)}{A_k} \right)\;, \tag{1.19}$$

the initial case $r = m$ is $F_m(\Theta) = \sum_{v \in W} \pi_k^{\mathcal{E}}(v) f_k(v) g_k(v) = B_m(\Theta) = A_m \cdot \frac{B_m(\Theta)}{A_m}$. The induction step is completed by

$$
\begin{aligned}
F_r &\overset{(1.18)}{=} B_r(\Theta) \prod_{l>r}^{m} A_l \;+\; A_r \cdot F_{r+1}(\Theta) \\
&= B_r(\Theta) \prod_{l>r}^{m} A_l \;+\; A_r \left(\prod_{l>r} A_l \right) \left(\sum_{k>r} \frac{B_k(\Theta)}{A_k} \right) \\
&= \frac{B_r(\Theta)}{A_r} \prod_{l \geq r}^{m} A_l \;+\; \left(\prod_{l \geq r} A_l \right) \left(\sum_{k>r} \frac{B_k(\Theta)}{A_k} \right) \\
&= \left(\prod_{l \geq r} A_l \right) \left(\sum_{k \geq r} \frac{B_k(\Theta)}{A_k} \right)
\end{aligned}
\tag{1.20}
$$

We realize that

$$
\begin{aligned}
c &= \sum_{H} P(D \mid H, \Theta^t) \pi^{\mathcal{E}}(H) \overset{(1.13),\, \pi^{\mathcal{E}}(H) = \prod_{k \in \mathcal{E}} \pi_k^{\mathcal{E}}(He_k)}{=} \sum_{v_1, \ldots, v_m \in W} \left(\prod_{k=1}^{m} \pi_k^{\mathcal{E}}(v_k) f_k(v_k) \right) \\
&= \prod_{k=1}^{m} \sum_{v_k \in W} \pi_k^{\mathcal{E}}(v_k) f_k(v_k) = \prod_{k=1}^{m} A_k
\end{aligned}
\tag{1.21}
$$

(note that $g_k(d_j, \Theta) = e_k^T R^T \Theta d_j = (R^T \Theta)_{kj}$). Note that for a deterministic prior, fixing an effects gene assignment $H \in \{0,1\}^{n \times m}$,

$$
\begin{aligned}
\log c &= \sum_{k=1}^{m} \log A_k \\
&= \sum_{k=1}^{m} \log \left(\sum_{j=0}^{n} \pi_{jk}^{\mathcal{E}} f_k(d_j) \right) = \sum_{k=1}^{m} \log \left(\sum_{j=0}^{n} \pi_{jk}^{\mathcal{E}} \exp\; g_k(d_j, \Theta^t) \right) \\
&= \sum_{k=1}^{m} \log \left(\sum_{j=0}^{n} \pi_{jk}^{\mathcal{E}} \exp\, (R^T \Theta^t)_{kj} \right) \\
&= \sum_{k=1}^{m} \log \left(H_{\bullet k} \exp\, (R^T \Theta^t)_{k \bullet} \right) \\
&\overset{H \in \{0,1\}^{n \times m}}{=} \sum_{k=1}^{m} H_{\bullet k} (R^T \Theta^t)_{k \bullet} \\
&= tr \left(H R^T \theta^t \right)
\end{aligned}
\tag{1.22}
$$

Finally, we obtain

$$
\begin{aligned}
\tilde{Q}(\Theta; \Theta^t) &\overset{(1.15)}{=} c^{-1} \cdot F(\Theta) \\
&\overset{(1.20),(1.21)}{=} \left(\prod_{k=1}^{m} A_k \right)^{-1} \cdot \left(\prod_{l=1}^{m} A_l \right) \left(\sum_{k=1}^{m} \frac{B_k(\Theta)}{A_k} \right) \\
&= \sum_{k=1}^{m} \frac{B_k(\Theta)}{A_k}
\end{aligned}
\tag{1.23}
$$

1.3. **The M-step.** According to Eq. (3) in the main text and (1.23) we have to maximize

$$\tilde{Q}(\Theta;\Theta^t) + \log \pi^S(\Theta) \quad = \quad \sum_{k\in\mathcal{E}} \frac{B_k(\Theta)}{A_k} + \log \pi^S(\Theta) \quad . \tag{1.24}$$

As a further simplification we assume edgewise independent priors:

$$\pi^S(\Theta) \quad = \quad \prod_{i,j} (\pi^S_{ij})^{\Theta_{ij}} (1-\pi^S_{ij})^{1-\Theta_{ij}} \ , \text{ with } 0 \le \pi^S_{ij} \le 1 \tag{1.25}$$

(we may disregard the cases in which $\pi^S_{ij} \in \{0,1\}$, because this means that the corresponding edge Θ_{ij} is fixed as absent or present and is therefore not subject to optimization). The log of the prior is then a linear function in each Θ_{ab}:

$$\begin{aligned} \log \pi^S(\Theta) &= \log \prod_{i,j} \pi_{ij}^{\Theta_{ij}} (1-\pi_{ij})^{1-\Theta_{ij}} \\ &= \sum_{i,j} [\, \Theta_{ij} \log \pi_{ij} + (1-\Theta_{ij}) \log(1-\pi_{ij}) \,] \\ &= \sum_{i,j} [\, \Theta_{ij} (\log \pi_{ij} - \log(1-\pi_{ij}) + \log(1-\pi_{ij}) \,] \\ &= \sum_{i,j} \Theta_{ij} \log \frac{\pi_{ij}}{1-\pi_{ij}} \ + \ \text{const} \ =: \sum_{i,j} \Theta_{ij}\tau_{ij} \ + \ \text{const} \end{aligned} \tag{1.26}$$

This implies that the objective function (1.24) $\tilde{Q}(\Theta;\Theta^t) + \log \pi^S(\Theta)$ is a polynomial in the variables $\{\Theta_{ab} \mid a = 1, ..., n; b = 1, ..., n\}$ of total degree 1. The partial derivatives of the objective function with respect to Θ_{ab} are therefore constant, i.e., independent of Θ (Note that Θd_j equals the j-th column of Θ, so $g_k(d_j, \Theta) = e_k^T R^T \Theta d_j$ is linear in the entries of Θ):

$$\begin{aligned} \frac{\partial g_k(d_j,\Theta)}{\partial \Theta_{ab}} &= \frac{\partial}{\partial \Theta_{ab}} [(e_k^T R^T)(\Theta d_j)] \ = \ \frac{\partial}{\partial \Theta_{ab}} \sum_{i=1}^n R_{ik}\Theta_{ij} \\ &= \sum_{i=1}^n R_{ik} \frac{\partial}{\partial \Theta_{ab}} \Theta_{ij} \ = \ \delta_{j=b} R_{ak} \ . \end{aligned} \tag{1.27}$$

Hence

$$\begin{aligned} \frac{\partial B_k(\Theta)}{\partial \Theta_{ab}} &= \sum_{i=0}^n \pi^{\mathcal{E}}_{ik} f_k(d_i,\Theta^t) \frac{\partial}{\partial \Theta_{ab}} g_k(d_s,\Theta) \ \overset{(1.27)}{=} \ \sum_{i=0}^n \pi^{\mathcal{E}}_{ik} f_k(d_i,\Theta^t)\delta_{i=b}R_{ak} \\ &= \pi^{\mathcal{E}}_{bk} f_k(d_b,\Theta^t) R_{ak} \end{aligned} \tag{1.28}$$

Consequently,

$$\frac{\partial \tilde{Q}(\Theta;\Theta^t)}{\partial \Theta_{ab}} \quad = \quad \frac{\partial}{\partial \Theta_{ab}} \sum_{k=1}^m \frac{B_k(\Theta)}{A_k} = \sum_{k=1}^m \frac{\pi^{\mathcal{E}}_{bk} f_k(d_b,\Theta^t) R_{ak}}{A_k} \ , \tag{1.29}$$

which together with (1.26) implies

$$\frac{\partial(\tilde{Q}(\Theta;\Theta^t) + \log \pi^S(\Theta))}{\partial \Theta_{ab}} \quad = \quad \sum_{k=1}^m \pi^{\mathcal{E}}_{bk} R_{ak} \exp(g_k(d_b,\Theta^t))(A_k)^{-1} \ + \ \tau_{ab} \ , \tag{1.30}$$

Using the step function $\text{step}(x) = \begin{cases} 1 & \text{if } x > 0 \\ 0 & \text{if } x \le 0 \end{cases}$, the updated values in Θ^{t+1} can be stated in closed form:

$$\Theta^{t+1}_{ab} \quad = \quad \text{step} \left\{ \sum_{k=1}^m R_{ak} \pi^{\mathcal{E}}_{bk} \exp((R^T\Theta^t)_{kb})(A_k)^{-1} + \tau_{ab} \right\} \ . \tag{1.31}$$

In the general case of an arbitrary prior $\pi^S(\Theta)$, it can be difficult to find a global optimum of the objective function $\tilde{Q}(\Theta; \Theta^t) + \log \pi^S(\Theta)$. However it is not necessary to find a global optimum, it is sufficient to find a Θ^{t+1} that increases the value of the objective function over the current value $\tilde{Q}(\Theta; \Theta^t) + \log \pi^S(\Theta^t)$. It has been shown in [3] that such a "stepwise" EM still converges to a local maximum of $P(\Theta \mid D)$. Therefore, we start with $\Theta = \Theta^t$ and go through all edges Θ_{ab} in a random order and check whether alteration of Θ_{ab} improves the objective function. If yes, we perform this change in Θ and continue until all edges were checked. The resulting Θ is our new Θ^{t+1}.

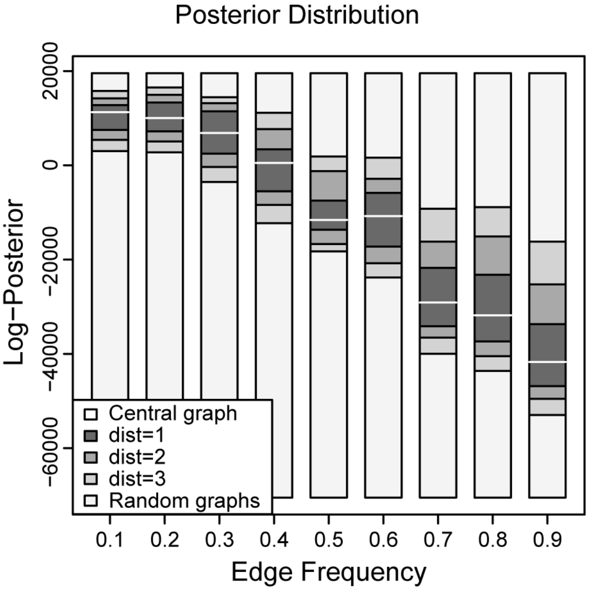

Figure 2.1. Posterior Distribution. This figure illustrates how the likelihood varies when only few edges (here: 1 to 3) are changed, based on randomly sampled fixed graphs (white lines) and relative to a fixed representative random graph sample (light gray). The underlying data is the real Mediator perturbation data, where Med10 and Med21 are combined to one signal node (i.e., $\mid S \mid = 9$), as a prior for the effects graph the data driven prior has been used (according to the initialization of the MCMC sampling). On the x-axis, different graph densities are compared. The strong variation within very similar graphs, demonstrates how rugged the landscape is. Given that the underlying data of this figure is the real data, the observed decrease of likelihoods following the increase of edge frequency yields extra information: It shows that the true Mediator graph we are looking for tends to be rather sparse, which is a confirmation for the choice of the earlier mentioned sparseness prior during MCMC sampling.

2. MCMC sampling

2.1. **MCMC sampling in general.**

The Metropolis-Hastings algorithm.

(1) Initialize Θ_0
(2) Proposal step: Given Θ_n, draw a candidate $\Theta^{'}$ from the proposal distribution $q(\Theta_n \to \Theta^{'})$
(3) Acceptance step: With probability $\min(1, \frac{L(\Theta^{'}) \cdot \pi(\Theta^{'}) \cdot q(\Theta^{'} \to \Theta_n)}{L(\Theta_n) \cdot \pi(n_n) \cdot q(n_n \to \Theta^{'})})$, let $\Theta_{n+1} = \Theta^{'}$ (accept). Otherwise, let $\Theta_{n+1} = \Theta_n$ (reject).
(4) Increment n by one and repeat steps 2. and 3. until convergence

Convergence. Starting from a (generally) randomly chosen initial parameter value, it takes some time until the chain converges to the true probability distribution. Thus, the first part of the chain, the so-called burnin phase is removed and only the so-called stationary phase is used for further analysis.

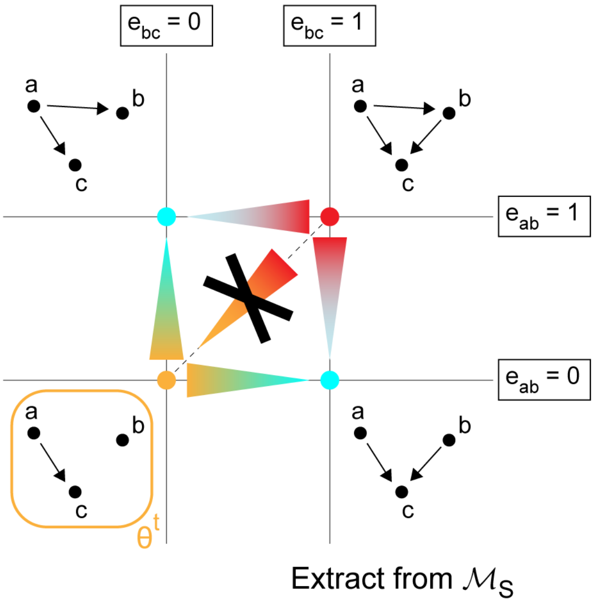

Figure 2.2. A classical situation in which EMiNEM gets stuck in a local maximum. Considering an extract of $\mathcal{M}_{\mathcal{S}}$, where Θ includes the edge $a \rightarrow c$, two different states are possible: either both edges $a \rightarrow b$ and $b \rightarrow c$ are missing (medium probability, indicated by orange) or both of them exist (high probability, indicated by red). A graph which includes only one of them has a low probability (indicated by blue). Thus, based on a $\Theta^t = \{a \rightarrow c\}$, EMiNEM is not able to cross the low-probability states to arrive at the high probability state, changing only one edge at the same time.

2.2. **MCMC for EMiNEM.** In the following, the most important elements of the algorithm are shortly described.

Chain length. Each chain consists of 60.000 steps. A major component of Markov Chain Monte Carlo sampling is the decision after how many steps the chain has converged, i.e., how many steps have to be excluded at the beginning of the sequence, such that the final part reliably represents the desired posterior distribution. Here, this decision is trivial: traceplots of the simulation runs showed, that after well less than the 60.000 steps the chain converges to one final $\hat{\Theta}$, i.e., the MCMC sampling can be seen as an additional EM algorithm (see Fig. S3.3). The MCMC runs of the Mediator data showed the same behavior (see Fig. S4.4). Thus, any information drawn from this final part of the sequence is good. However, for reasons of consistency, and since the effect gene attachment is updated every 5000 steps (as described before), only parameters according to the final attachment, i.e., the 5000 last parameters of the sequence are retained.

Prior information. Since nothing is known about the signals graph, a uniform prior is chosen (i.e., edge frequency = 0.5). For the effects graph, an (edgewise independent) prior is calculated for each effect node, as explained in the main text. However, if additional prior information on either the signals graph or the effects graph is available, its incorporation can speed up convergence time.

Initial parameter. The chain is initialized with a randomly sampled signals graph, based on a sparse edge frequency. Independence of the chain from the initial signals graph (which is an essential property of Markov Chains) has been verified by simulation.

Proposal function. At each step, a new candidate parameter $\Theta^{'}$ is suggested based on the last one (Θ_n). The crux is to choose a proposal function $q(\Theta_n \rightarrow \Theta^{'})$, such that it is a trade-off between steps that are big enough to scan the whole parameter space in reasonable time, but small enough to still being accepted. Simulation has shown that randomly selecting $1.5 \cdot |S|$ edges and replacing them according to the predefined edge frequency results in both sufficient acceptance and good mixing of the chain.

Acception / Rejection. The newly suggested parameter is accepted (and added to the chain), if

$$\begin{aligned}\log(u) < \quad & \min(0, \Big(\log L(\hat{\Theta}^{'}) + \log \pi(\hat{\Theta}^{'}) + \log q(\Theta^{'} \rightarrow \Theta_n)\Big) \\ & - \Big(\log L(\hat{\Theta}_n) + \log \pi(\hat{\Theta}_n) + \log q(\Theta_n \rightarrow \Theta^{'})\Big) \\ & + w_{\text{sparse}} \cdot \Big(\log \pi_{\text{sparse}}(\Theta^{'}) - \log \pi_{\text{sparse}}(\Theta_n)\Big)) \quad \text{, with } u \sim \mathcal{U}(0,1) \text{ ,}\end{aligned}$$

otherwise it is rejected and the old parameter is added once again. Note that Θ is the signals graph suggested by the proposal function, while $\hat{\Theta}$ is the corresponding local maximum derived by EMiNEM, as explained in the main text. We included an additional prior $\pi_{\text{sparse}}(\Theta) = \prod_{j,k} f_{\text{edge}}^{\Theta_{jk}} \cdot (1 - f_{\text{edge}})^{(1-\Theta_{jk})}$ for sparsity of the sampled graph (f_{edge} is the expected relative edge abundance of the sampled graph). The corresponding weighting parameter $w_{\text{sparse}} = 0.5$ has been determined empirically during simulation. Moderate variation of w_{sparse} did not change the results qualitatively (data not shown).

Resulting signals graph. The Markov Chain provides an approximation of the posterior distribution of the sampled parameters. We extract one “resulting” signals graph from this chain by weighting all edges by their frequency in the last 5000 steps and only retaining those that appear in at least 50%. In general, this marginalization might result in a loss of information because dependencies between edges are not considered any more. However, since the Markov Chain in our case converges very fast to a unique, dominating signals graph which then will be extracted as the resulting graph, there basically is no marginalization and so this problem does not arise here.

2.3. **A theoretical motivation for the sampling of local maxima.** EMiNEM is viewed as a function $EM : \Theta \mapsto \hat{\Theta} = EM(\Theta)$, which maps the signals graph space $\mathcal{M}_{\mathcal{S}}$ onto the space $\mathcal{N} = EM(\mathcal{M}_{\mathcal{S}})$ of local maxima of the posterior. The current paragraph is devoted to constructing a sequence in $\mathcal{N}$ that provides a representative sample of $P|_{\mathcal{N}}$, the restriction of the posterior probability P to $\mathcal{N}$. Our task is complicated by the fact that we cannot construct functions that sample from $\mathcal{N}$ directly, because the calculation of each member requires the application of EMiNEM. Instead, we use Metropolis-Hastings Markov Chain Monte Carlo (MCMC) sampling [4–6] to construct a sequence in $\mathcal{M}_{\mathcal{S}}$, and lift it to $\mathcal{N}$ (see Supplements S2.2 for details on our implementation). Let $(\Theta_i)_{i=1,2,...}$ be a sequence of signals graphs in $\mathcal{M}_{\mathcal{S}}$ obtained by MCMC sampling from the distribution P. The corresponding sequence $(EM(\Theta_i))_{i=1,2,...}$ is then an approximate empirical sample from the distribution $\hat{P}(\hat{\Theta}) = \sum\{P(\Theta \mid D);\ EM(\Theta) = \hat{\Theta}\} \approx P|_{\mathcal{N}}(\hat{\Theta})$ on $\mathcal{N}$. This approximation is valid under the assumption that the probability of $P(\hat{\Theta} \mid D)$ is substantially larger than $P(\Theta \mid D)$ for all other $\Theta \in EM^{-1}(\hat{\Theta})$, which is presumably the case. However, the convergence speed of this Markov chain is very slow, the reason being implicit in the assumption: In order to move from one local maximum to a different one, the underlying Markov Chain in $\mathcal{M}_{\mathcal{S}}$ needs to traverse regions of substantially lower probability. We remove this obstacle by sampling $(\Theta_i)_{i=1,2,...}$ from the distribution $Q(\Theta) \propto P(EM(\Theta) \mid D)$ instead of sampling from P. The corresponding sequence $(EM(\Theta_i))_{i=1,2,...}$ is then an approximate empirical sample from the distribution

$$\begin{aligned}\hat{P}(\hat{\Theta}) &\propto \sum\{P(EM(\Theta) \mid D);\ EM(\Theta) = \hat{\Theta}\} = P(\hat{\Theta} \mid D) \cdot |\{\Theta;\ EM(\Theta) = \hat{\Theta}\}| \\ &\approx P(\hat{\Theta} \mid D) \cdot c \end{aligned} \tag{2.1}$$

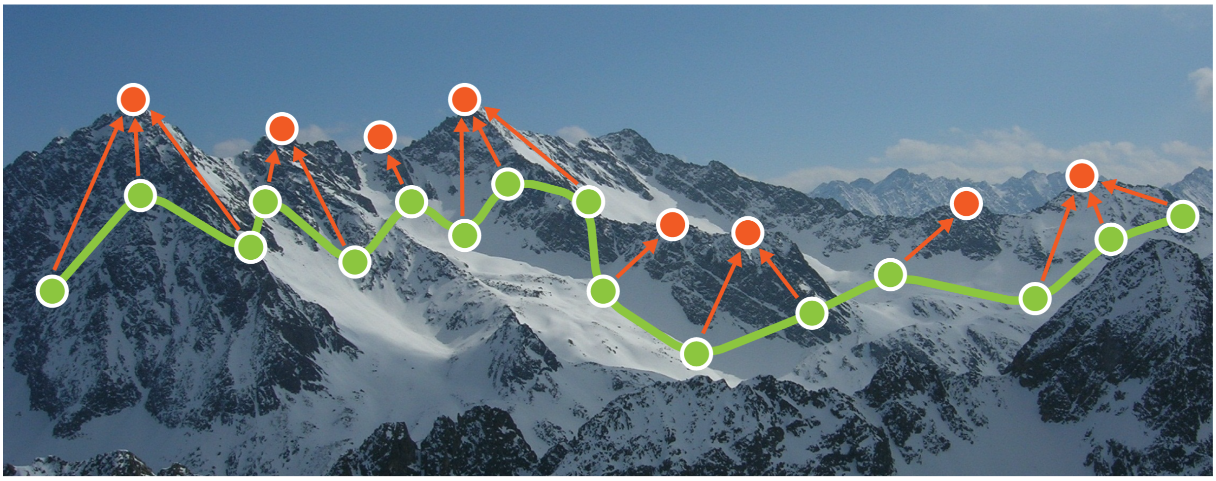

Figure 2.3. The search strategy used by MC EMiNEM can be perfectly illustrated based on this mountain view from the top of the Hintere Karlesspitze in the Stubai Alps. The green dots represent the sampled signals graphs $\Theta_1, \Theta_2, ... \in \mathcal{M}_{\mathcal{S}}$ forming the underlying Markov chain (the green line). At every step the EM algorithm is applied to identify the corresponding local maxima $\hat{\Theta}_j \in N$ (the red dots), and the decision on acceptance or rejection of a new proposition Θ' is based on the corresponding local maximum $\hat{\Theta}'$.The sequence $\hat{\Theta}_1, \hat{\Theta}_2, ...$ is then approximately a representative sample of the posterior distribution of the local maxima. This combination of Expectation Maximization and MCMC sampling offers a way to restrict the sequence derived from the sampling process to the most informative parameters.

The last approximation assumes that the pre-image of $\hat{\Theta}$ under EM has a similar size c for all $\hat{\Theta} \in \mathcal{N}$. In any case, we expect the relative probability $\frac{\hat{P}(\hat{\Theta}_1)}{\hat{P}(\hat{\Theta}_2)}$ to be dominated by the quotient $\frac{P(\hat{\Theta}_1|D)}{P(\hat{\Theta}_2|D)}$, which justifies our approximation in Equation (2.1) for the purpose of finding high-scoring graphs $\hat{\Theta}$.

2.4. **Empirical Bayes estimation of the effects graph prior.** The attachment probability H^i_{jk} of effect node k to signal node j, based on one signals graph Θ^i, is:

$$H^i_{jk} = P(H^i_{\bullet k} = e_j | \Theta^i, R, H^{old}) = \frac{\exp f^i_k(j)}{\sum_j \exp f^i_k(j)} \text{ , with}$$

$$f^i_k(j) = \begin{cases} \log \pi(H^{old}_{jk}) + R_{\bullet k}\Theta^i_{\bullet j} & \text{for } j \epsilon S \\ \log \pi(H^{old}_{jk}) & \text{for } j \text{ the null node} \end{cases}$$

The new attachment probability, based on the preceding $N = \frac{|chain|}{12}$ steps of the Markov Chain, is then $H^{new} = \frac{\sum_{i=1}^N H^i}{N}$.

In our approach, we do not sample from $\mathcal{M}_{\mathcal{S}}$ directly, but we sample from a set of local maxima $\mathcal{N}$. This set is much smaller and develops slower than $\mathcal{M}_{\mathcal{S}}$, as can be seen in the traceplots. Note that this set changes every epoch, since the prior is updated empirically.

3. Simulation

3.1. **Data generation.** Simulated data has been generated using the method createNEM, provided by Nessy [7]. It takes as input the number of signals $| \mathcal{S} |_{true}$ and effect genes $| \mathcal{E} |_{true}$, as well as the two

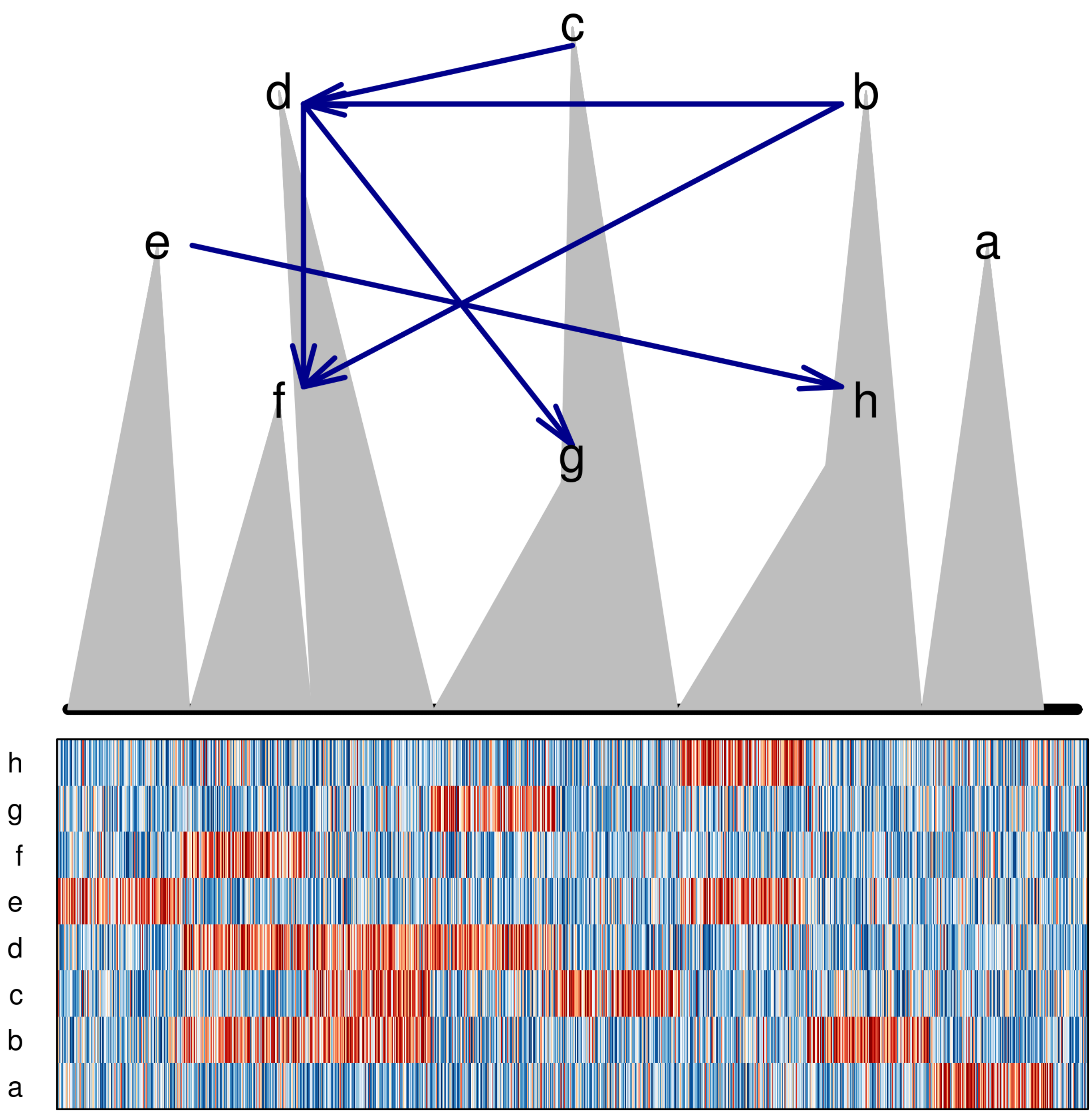

Figure 3.1. A simulated Nested Effects Model for $\mid \mathcal{S} \mid = 8$ and $\beta - level = 49\%$. Above, the signals graph is shown, below the corresponding R matrix, clustered according to the gene attachment (rows: perturbations, columns: effects on measured genes). Red color indicates a positive log-ratio value, blue color indicates a negative log-ratio value. The stronger the color of a field R_{kj}, $k \in \mathcal{E}$, $j \in \mathcal{S}$, the higher the probability that the measured data is due to the fact that there actually is an effect of signal j on gene k, or, that there is no effect, respectively.

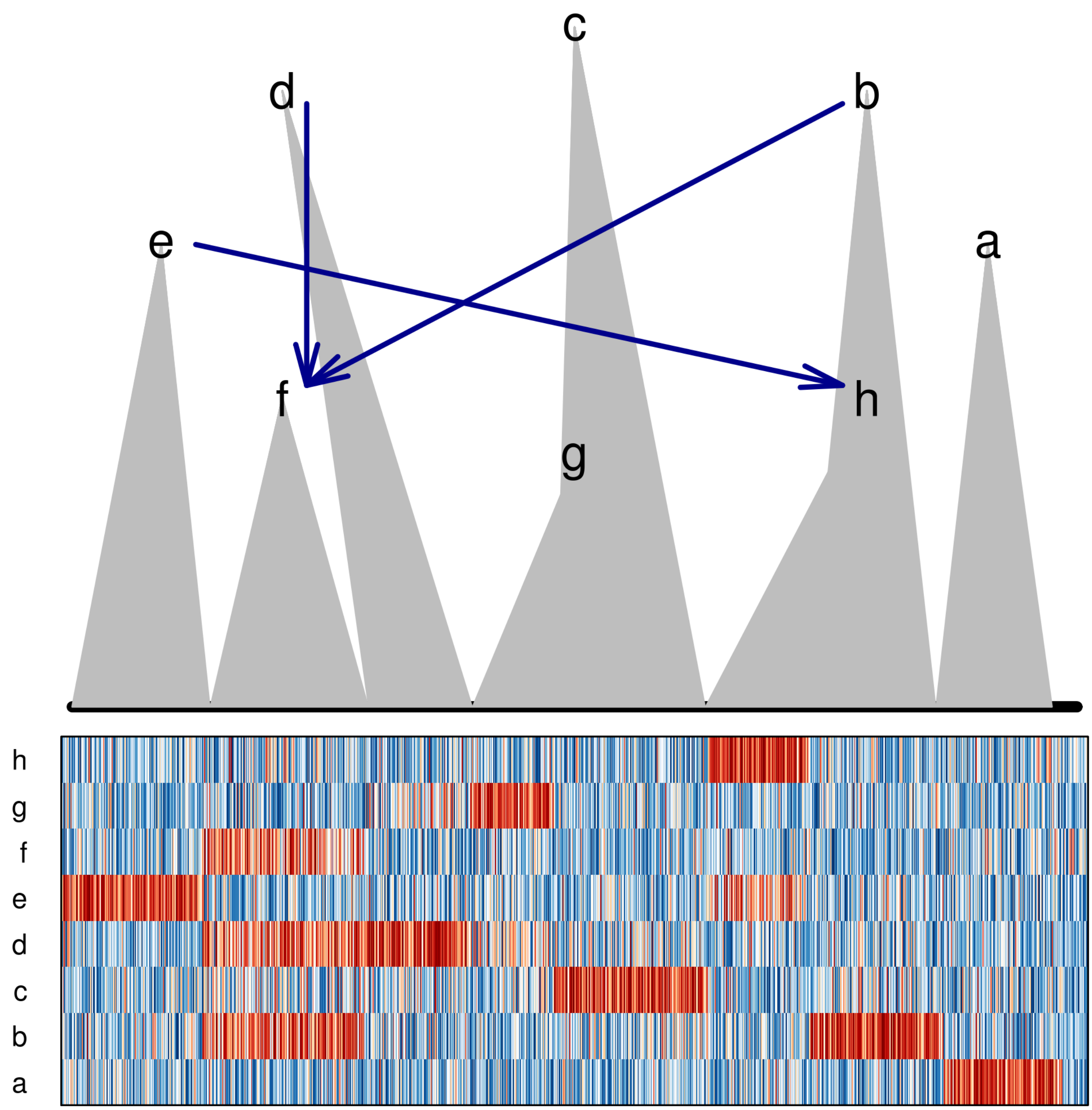

Figure 3.2. The prediction for the simulated NEM in Fig. S3.1. Above, the resulting signals graph is shown, below the underlying R matrix, clustered according to the final gene attachment (rows: perturbations, columns: effects on measured genes). Red color indicates a positive log-ratio value, blue color indicates a negative log-ratio value. The stronger the color of a field R_{kj}, $k \in \mathcal{E}$, $j \in \mathcal{S}$, the higher the probability that the measured data is due to the fact that there actually is an effect of signal j on gene k, or, that there is no effect, respectively. The R matrix here is the same as in Fig. S3.1, but the ordering of genes (columns) is different, since it depends on the gene attachment derived by the MCMC sampling.

noise parameters μ and δ. Θ_{true} and H_{true} are randomly sampled according to $\mid \mathcal{S} \mid_{true}$ and $\mid \mathcal{E} \mid_{true}$ and the true data matrix ($\mid \mathcal{S} \mid_{true} \times \mid \mathcal{E} \mid_{true}$) is calculated according to these graphs. A noisy log-odds ratio matrix is then calculated based on the true effects by sampling its values from two normal distributions with ($mean = -\frac{\mu}{2}$, $sd = \delta$) and ($mean = \frac{\mu}{2}$, $sd = \delta$), respectively. μ and δ have been chosen such that the $\alpha - level$ and the $\beta - level$ have the values described in the main text. A simulated NEM and the corresponding prediction of MC EMiNEM, for $\mid \mathcal{S} \mid= 8$ and $\beta - level = 49\%$ are shown in Fig. S3.1 and Fig. S3.2.

3.2. **Behavior of the MCMC chain.**

Convergence. The convergence of the Markov chain is important in order to get a representative sample of parameter values. It has been verified in simulation runs, as outlined in the main part of this paper. Traceplots for the example mentioned above (Fig. S3.1, Fig. S3.2) are shown in Fig. S3.3 (all edges) and Fig. S3.4 (selected edges).

Attachment of effects. The development of the attachment of effects to signal nodes during the Empirical Bayes procedure is visualized in Fig. S3.5. The attachment predicted by MC EMiNEM is compared to the true one in Fig. S3.6.

Independence of initialization. For six simulated NEMs, randomly chosen from two parameter settings (one with $\mid \mathcal{S} \mid= 8$ and $\beta - level = 49\%$, the second with $\mid \mathcal{S} \mid= 11$ and $\beta - level = 20\%$), 10 runs each initialized with a different signals graph have been performed. For all of the six datasets, the ten results where the same, i.e., independent of initialization (data not shown).

	$\lvert\mathcal{E}\rvert = 1000$				$\lvert\mathcal{E}\rvert = 5000$			
	MC EMiNEM [min]	EMiNEM [s]	nem [s]	Nessy [s]	MC EMiNEM [min]	EMiNEM [s]	nem [s]	Nessy [s]
$\lvert\mathcal{S}\rvert = 5$	25	0.03	1.61	0.09	130	0.13	13	0.55
$\lvert\mathcal{S}\rvert = 8$	27	0.03	4.40	0.14	244	0.25	14	0.35
$\lvert\mathcal{S}\rvert = 11$	29	0.03	2.44	0.1	1320	1.32	29	0.38

	MC EMiNEM	EMiNEM	nem	Nessy
Mediator data	104 min	0.1 s	26 s	0.3 s

Table 1. The three available NEM implementations are compared with regard to their run time (seconds, resp. minutes for MC EMiNEM). The upper table is based on simulated (and randomly generated) datasets and includes varying signals graph and effects graph sizes. The lower table is based on the Mediator data. The run times for MC EMiNEM include $6 \cdot 10^4$ MCMC steps, however, this number may be reduced, if the convergence speed of the Markov chain is sufficiently high.

3.3. **Prediction quality.**

To assess the prediction quality, MC EMiNEM has been compared to four other methods. In the following, the results of this comparison (as depicted in figure 2 A) are discussed and a detailed explanation of the four methods is provided. In all cases, the priors for the signals graph and for the effects graph are the same, as described in the main text, to ensure a fair basis for comparison.

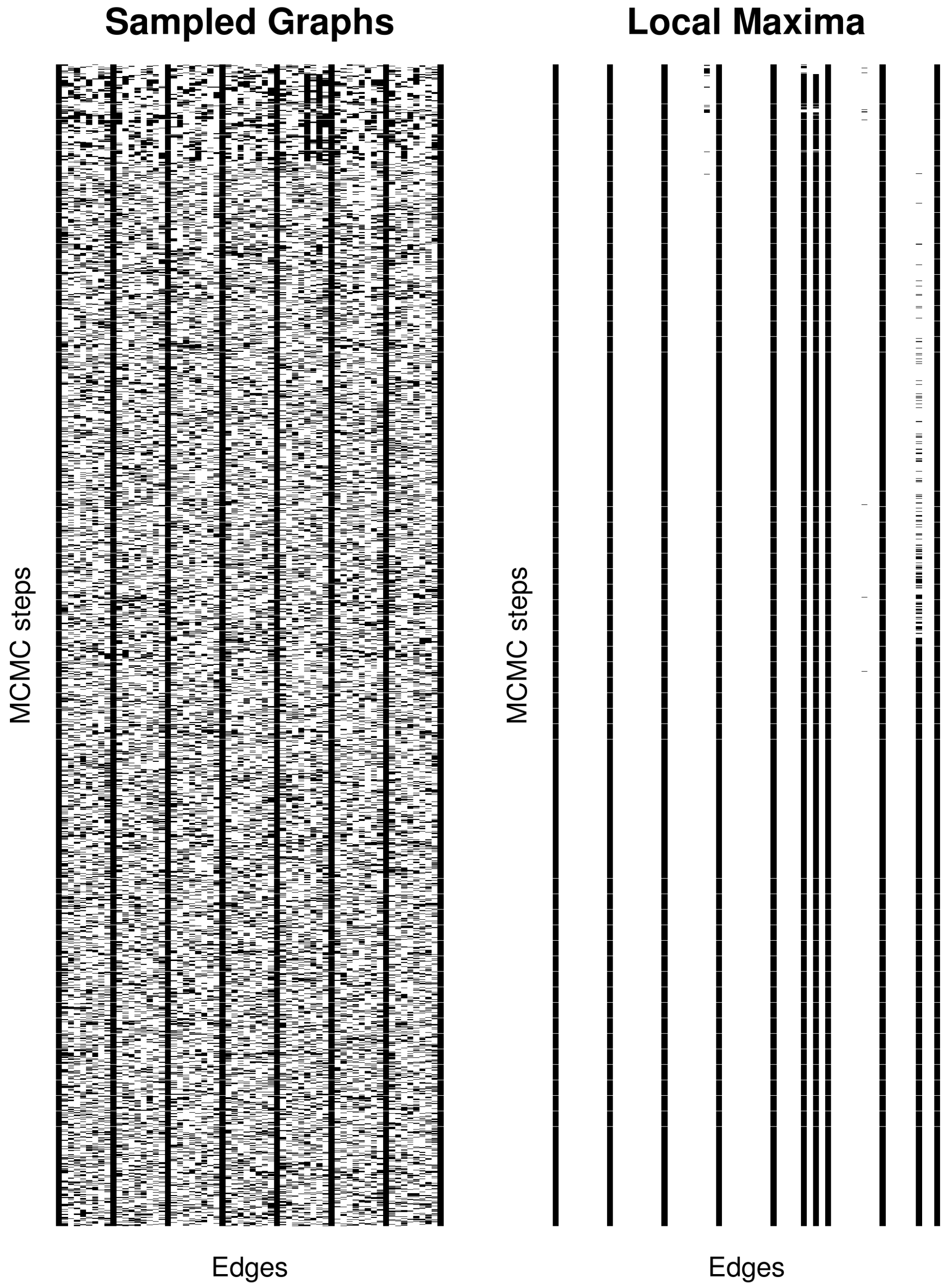

Figure 3.3. Traceplot of one MCMC run. Here, $\mid \mathcal{S} \mid = 8$ and $\beta - level = 49\%$. The left panel shows the traceplot for the sampled graphs $(\Theta_i)_{i=1,2,...}$, the right panel shows the traceplot for the corresponding local maxima $(\hat{\Theta}_i)_{i=1,2,...}$. The MCMC steps are depicted on the y-axis (from top to bottom), individual edges on the x-axis, thus, one line in the traceplot corresponds to the signals graph of the corresponding MCMC step. Black fields indicate the presence, white fields the absence of a given edge in a given MCMC step. Completely black columns represent self-loops, which are defined to be present in the mathematical formulation and included here for reasons of clarity. Since various signals graphs can yield the same local maxima, the sampled graphs vary strongly throughout the whole sampling process, while the local maxima vary slower and in a more restricted model space and converge in the second half of the Markov chain. This behavior has been discussed extensively in the main text.

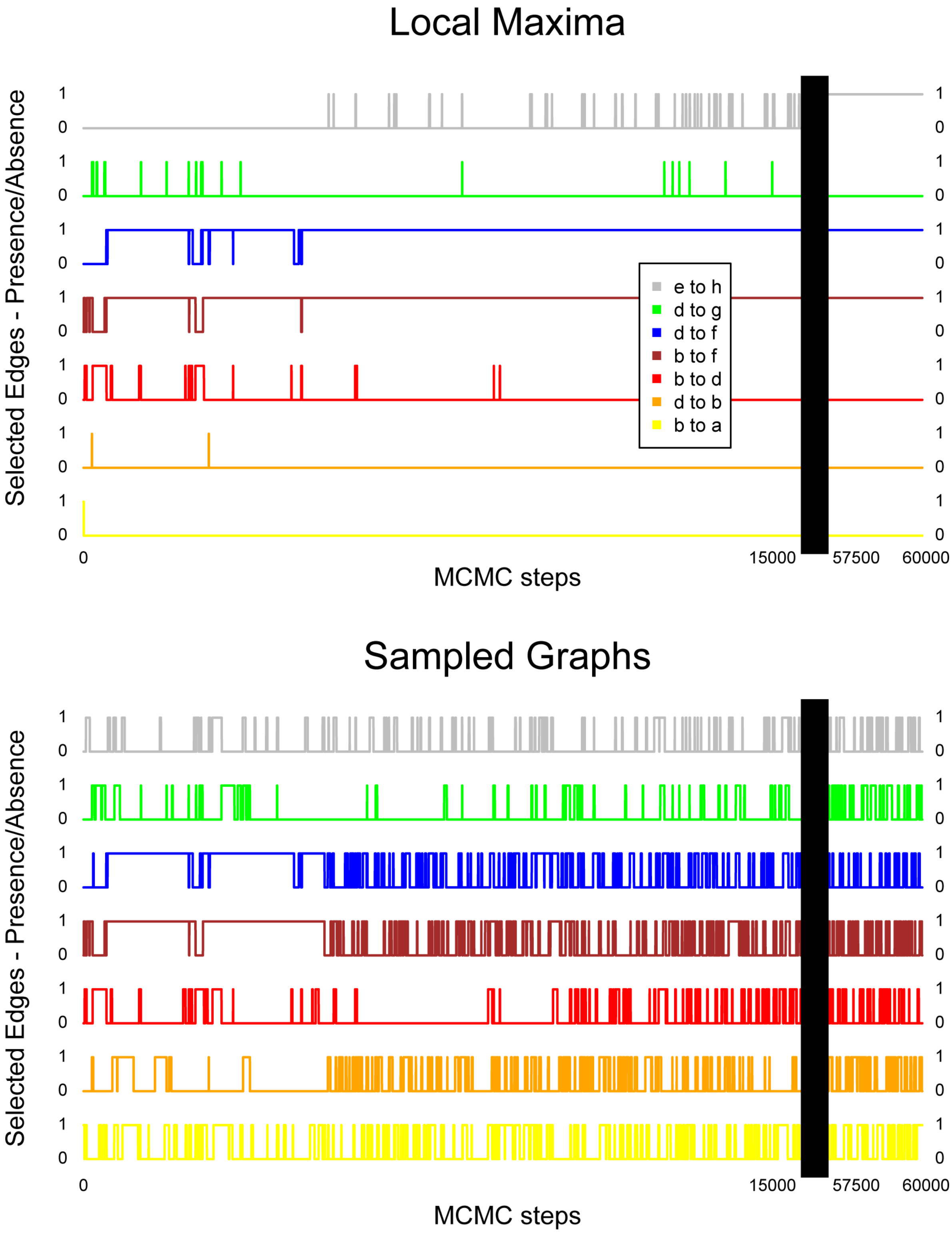

Figure 3.4. Traceplots of selected edges. Here, $\mid \mathcal{S} \mid = 8$ and $\beta - level = 49\%$. The upper panel shows the traceplots of selected edges in the sequence of local maxima $(\hat{\Theta}_i)_{i=1,2,\ldots}$, the lower panel shows the traceplot of these edges in the sequence of the underlying sampled signals graphs $(\Theta_i)_{i=1,2,\ldots}$. On the x-axis, extracts of the MCMC steps at the beginning $(1-1500)$ and the end $(57500-60000)$ of the chain are depicted. Selected edges (edges, that vary in the sequence of local maxima) are depicted in different colors. Stacked on the y-axis are values of 0 and 1 for each edge, corresponding to the absence and presence of the edge at a given MCMC-step. The traceplots here show the same behavior as has already been discussed in Fig. S3.3.

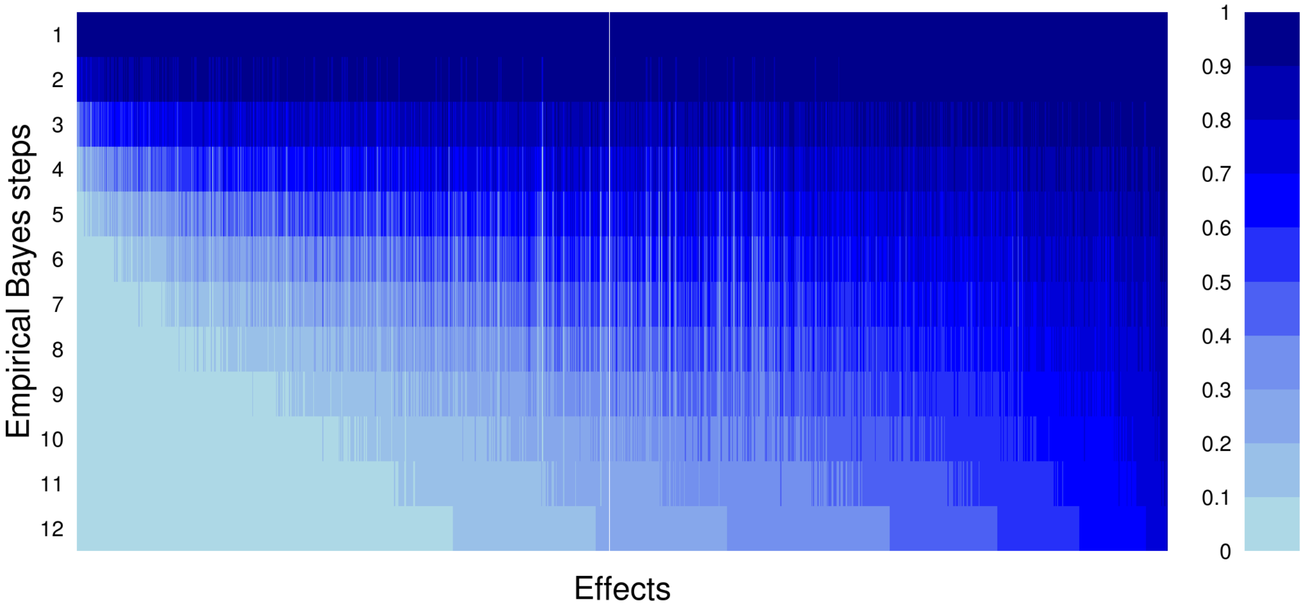

Figure 3.5. Development of attachment entropy. For each effect j in each Empirical Bayes step l, the Shannon Entropy is calculated as follows: $-\sum_{j \in \mathcal{S}} H^l_{jk} \cdot log_2 H^l_{jk}$. On the y-axis, the Empirical Bayes steps are depicted (from top to bottom), on the x-axis, the effects are listed. The colors indicate the entropy, relative to the maximal one (when, for a given effect, the attachment probability is the same for any signal node (or no signal node at all)). Obviously, even though the initial prior for the effects graph is calculated according to the data matrix, the entropy is still very high. During the Empirical Bayes procedure, some effects turn out to be quit deterministic, while others remain flexible until the end of the Markov chain.

Random. For each NEM, 5000 random signals graphs have been sampled, according to the parameters described in the main text. Every (unique) graph has than been weighted by its posterior and a consensus signals graph has been built including all edges with a (weighted) value of ≥ 0.5. This is the most trivial method for parameter estimation.
As expected, this method yields quit good results for small numbers of signal nodes, where the probability of randomly drawing reasonable graphs is higher. However, for larger number of signal nodes, independent of the noise level, this method is not able to detect the correct edges at all.

EMiNEM. This method is based on the random sampling approach, except that not the sampled signals graphs but their corresponding local maxima have been weighted and combined to a consensus signals graph.
EMiNEM is slightly better than random sampling, because by only taking into account local maxima unlikely graphs are excluded from the consensus. However, it still relies on random drawing of signals graphs and only yields good results for small numbers of nodes. By comparing it to the considerably better results of the more elaborate MC EMiNEM it is clearly visible that the more complex and time-consuming Markov Chain Monte Carlo approach, which leads to a reasonably "guided" sampling of the model space, is justified.

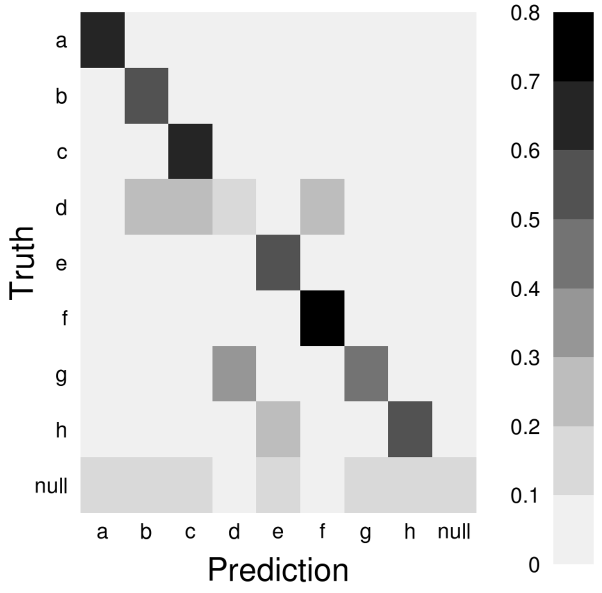

Figure 3.6. Prediction quality for the effects graph. Here, an entry in row i and column k depicts the probability that an effect, attached to signal node i in the true model, is attached to signal node k in the predicted model (i.e., rows correspond to the true attachment, columns to the predicted one). Light gray corresponds to low probabilities, dark gray to high ones (see the scale on the right-hand side). The predicted attachment corresponds very well to the true one in most cases, except for effects attached to signal node d - which corresponds very well to the missing edges including d during the prediction the signals graph (Fig. S3.2).

Nessy. Nessy is a publicly available NEM implementation, introduced by [1]. Unlike (MC) EMiNEM it's a maximum full likelihood / posteriori approach, where not only the maximum for the signals graph but also for the effects graph should be identified. Since no prior knowledge regarding the signals graph is available, but a sparse graph is assumed, Nessy is initialized with the empty graph.
MC EMiNEM is a maximum marginal posteriori approach, it only calculates the maximum for the signals graph and marginalizes over the effects graph. For good data, with low amount of noise, the effects graph is clearly identifiable and MC EMiNEM and Nessy perform comparably. However, for higher noise the calculation of the maximum effects graph is error-prone and the risk of getting stuck in the wrong model is high, so Nessy is clearly outperformed by MC EMiNEM there.

nem. nem is the original NEM implementation, publicly available through Bioconductor [8]. Recently, [9] published a review of all currently available NEM algorithms, where they recommend the Bayesian greedy hillclimbing approach for small networks as the method of choice. It calculates the original NEM score by integrating over all effects graphs. According to these findings, we applied nem on the log-odds ratios with the following parameters: inference="nem.greedy" and type="CONTmLLBayes". Again, we chose a signals graph prior and an effects graph prior as described in the main text.

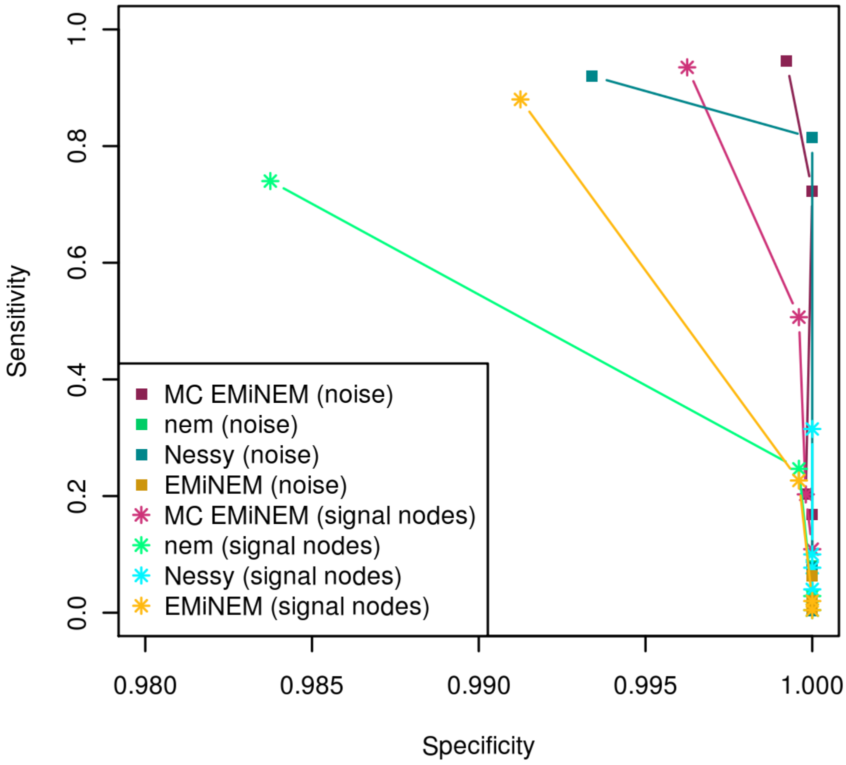

Figure 3.7. Cross-methods comparison of specificity and sensitivity. This figure displays the mean specificity (x-axis) and sensitivity (y-axis) values for all methods (indicated by different colors) for the parameter settings of Fig. 2A of the main text (i.e., varying noise levels or varying signals graph sizes, indicated by colors of different brightness and different plot symbols). Obviously, the specificity of all methods is very high, making sensitivity the distinctive feature, as it is shown in Fig. 2A)

4. Application

4.1. Sample preparation.

Med7N/Med31. This data has been taken from [10].

Med2/Med20/Med31. The S. cerevisiae strain used was BY4742 (Euroscarf): MATα, his2Δ1, leu2Δ0, lys2Δ0, ura3Δ0. The knockout mutants were from Euroscarf and validated by PCR. Samples were grown in YPD medium medium overnight, diluted to an OD600 of 0.1 the next day and grown to a final OD600 of 0.8. Cells were centrifuged at 4,000 rpm for 1 min and cell pellets were immediately flash frozen in liquid nitrogen. Total RNA was extracted using the RiboPure-Yeast Kit (Ambion/Life Technologies), following the manufacturer's protocol. Labeling of samples was performed using the GeneChip 3'IVT Express Labeling Assay (Affymetrix) with 250 ng input RNA. Labeled samples were hybridized to GeneChip Yeast Genome 2.0 microarrays following the instructions from the supplier (Affymetrix).

Med7C/Med10/Med19/Med21. The S. cerevisiae strains used were derivatives of SLY101: MATα ade-can1-100 cyh2r his3-11,15 leu2-3,112 trp1-1 ura3 [11]. Samples were grown in SD (synthetic complete) medium medium overnight, diluted to an OD600 of 0.1 the next day and grown to a final OD600 of 0.8. Cells were centrifuged at 4,000 rpm for 1 min and cell pellets were immediately flash frozen in liquid nitrogen. Total RNA was extracted using the RiboPure-Yeast Kit (Ambion/Applied Biosystems), following the manufacturer's protocol RNA was extracted as above. Labeling of samples was performed using the GeneChip 3'IVT labeling Assay (Affymetrix) with 100 ng input RNA. Labeled samples were hybridized to GeneChip Yeast Genome 2.0 microarrays following the instructions from the supplier (Affymetrix).

Med2/Med15/Med20. The S. cerevisiae strain used was BY4741 (Euroscarf): MATa, his3Δ1; leu2Δ0; met15Δ0; ura3Δ0. The knockout mutants with the same genomic background were from Euroscarf and validated by PCR. Samples were grown in YPD medium overnight, diluted to an OD600 of 0.15 the next day and grown to a final OD600 of 0.8. Cells were labeled with 4-Thiouracil for 6 minutes, then centrifuged at 3,500 rpm and 30°C for 1 min and cell pellets were resuspend in RNA Later and immediately flash frozen in liquid nitrogen. Cells were counted and mixed with labeled S.pombe cells 3:1. Total RNA was extracted using the RiboPure-Yeast Kit (Ambion/Life Technologies), following the manufacturer's protocol. Labeled RNA was separated by biotinylation and using the μMACS Streptavidin kit (Miltenyl Biotec) and followed by the RNeasy MinElute clean up kit (Qiagen). Labeling of the Total and Labeled RNAs was performed using the GeneChip 3'IVT Express Labeling Assay (Affymetrix) with 300 ng input RNA. Labeled samples were hybridized to GeneChip Yeast Genome 2.0 expression microarrays following the instructions from the supplier (Affymetrix).

4.2. Data processing. Data processing has been done using R [12].
The arrays were read in and transformed to expression values one by one, using expresso() from the **R**/*Bioconductor* package affy [13] with the following parameters for background correction and summarization: bgcorrect.method="rma",pmcorrect.method="pmonly",summary.method="avgdiff". Some arrays included S.pombe probes, they were filtered to S.cerevisiae. The median expression values were centered to zero (on the log-scale) for each array(this step has only the purpose of generating a sensible average expression distribution in the subsequent quantile normalization step). The expression values were log2 transformed and quantile normalization was performed afterwards using quantile.normalization() from the affy-package.
The **R**/*Bioconductor* package limma [14] was used for further assessment of differential gene expression. A design matrix was constructed that takes into account batch-specific effects as well as subunit-specific effects. The linear regression model was fitted using lmFit(). Finally, the log-odds ratios corresponding to subunit specific effects were extracted using ebayes(). The essential four lines of codes are listed below

(the design matrix *design* is the standard design matrix for multiple groups vs. one reference experiments, *expr* are the expression values derived from the data, *lodsmat* is the desired log-odds matrix R (see main text)):

```
fit = lmFit(expr, design)
eBayesObj = eBayes(fit)
lodsmat = eBayesObj$lods
```

To accommodate the different experiments that have been combined, contrasts with respect to batch effects have been created and fitted. Genes showing differential expression (here with a fold change ≥ 2.5) with respect to these contrasts were removed from the subsequent analysis. Additionally, genes that do not react to any perturbation (here with a log-odds ratio < 0 in all cases) were removed.
It is important to check the final result $R = (R_{jk})$ for artifacts. Sometimes, R contains erratic, extraordinarily high entries that dominate the likelihood term. This is due to the fact that limma performs a variant of the t-test, in which the variance term is estimated. Although this is done in a robust manner, it may still sometimes underestimate the true variance, leading to overly significant results. We advise the user to manually threshold the entries in the R-matrix (e.g., to the 99% quantile of the entries in the R matrix), if outliers in the R-matrix occur. In our Mediator application, this was not necessary.

4.3. **Gene set enrichment analysis for transcription factor targets.** The gene set enrichment analysis was done according to [15], using the **R**/*Bioconductor* package mgsa (version 1.2.0) [16], with the following parameters: p=seq(0.02,0.2,by=0.004), alpha=seq(0.02,0.98,by=0.02), beta=seq(0.02,0.98,by=0.02), steps=(5*1e6), restarts=10. Restraining p to small values ensures a sparse solution. For each gene set a mgsa run was performed, taking into account only TFs being mapped to at least one gene of the study set. The total population has been set to all effect genes being part of the corresponding Nested Effects Model. Only TFs being enriched with a probability of $\geqq$ 50% were valued as significant and further analyzed.
Two examples of enriched TFs are explained in more detail in the main text (see Fig. 5). Similar figures for all Mediator subunit - transcription factor pairs are provided as a separate file.

4.4. **Results.**

Predicted Nested Effects Models. Predicted NEMs are shown in Fig. S4.1, Fig. S4.2 and Fig. S4.3.

Convergence of the Markov Chain. Traceplots for the real data are shown in Fig. S4.4 (all edges) and Fig. S4.5 (selected edges).

Attachment of effects. The development of the attachment of effects to signal nodes during the Empirical Bayes procedure is visualized in Fig. S4.6.

Comparison with cluster analysis. In Fig. S4.7, the clustering of Mediator subunits and genes based on fold changes and log-odds ratios is depicted. Both approaches lead to an almost identical (isomorphic) dendrogram, which also agrees well with the MC EMiNEM's signals graph (if edge directions are ignored). This means that the coarse grouping of Mediator subunits can already be read off the expression profiles. However, MC EMiNEM provides more detailed information on the hierarchical structure of the Mediator organization, as well as on the attachment of effects. Fig. 4.8 compares the networks derived by MC EMiNEM and by the hierarchical clustering.

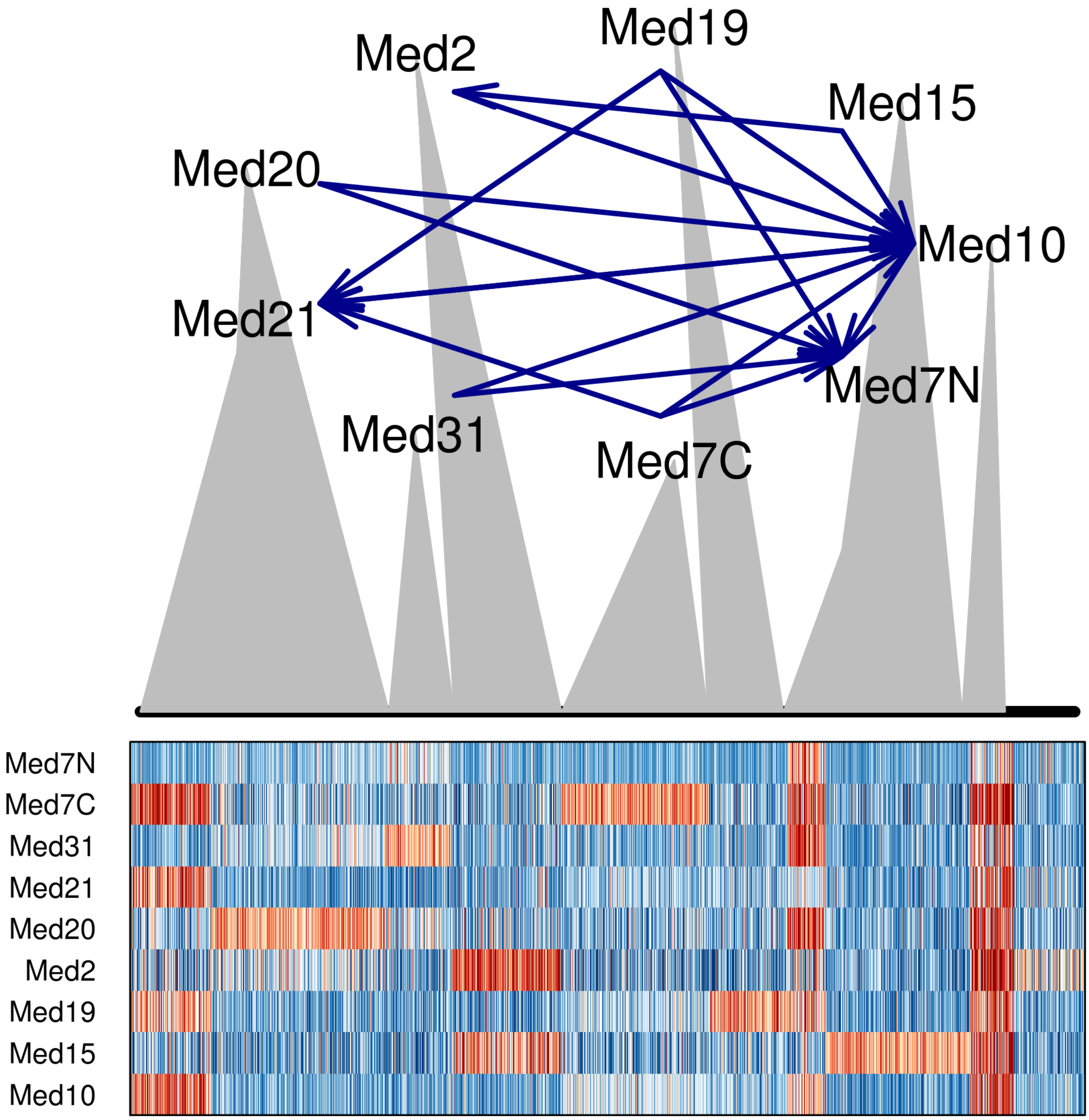

Figure 4.1. The Mediator-NEM treating all subunits as individual nodes, version 1 (result of nine runs out of 10). Above, the resulting signals graph is shown, below the underlying R matrix, clustered according to the final gene attachment (rows: perturbations, columns: effects on measured genes). Red color indicates a positive log-ratio value, blue color indicates a negative log-ratio value. The stronger the color of a field R_{kj}, $k \in \mathcal{E}$, $j \in \mathcal{S}$, the higher the probability that the measured data is due to the fact that there actually is an effect of signal j on gene k, or, that there is no effect, respectively. There exists an edge Med10 $\rightarrow$ Med21 as well as Med21 $\rightarrow$ Med10.The similarity between the two perturbations is also clearly visible in the perturbation profile. Thus, in the following, the two Mediator subunits are treated as one node in the NEM.

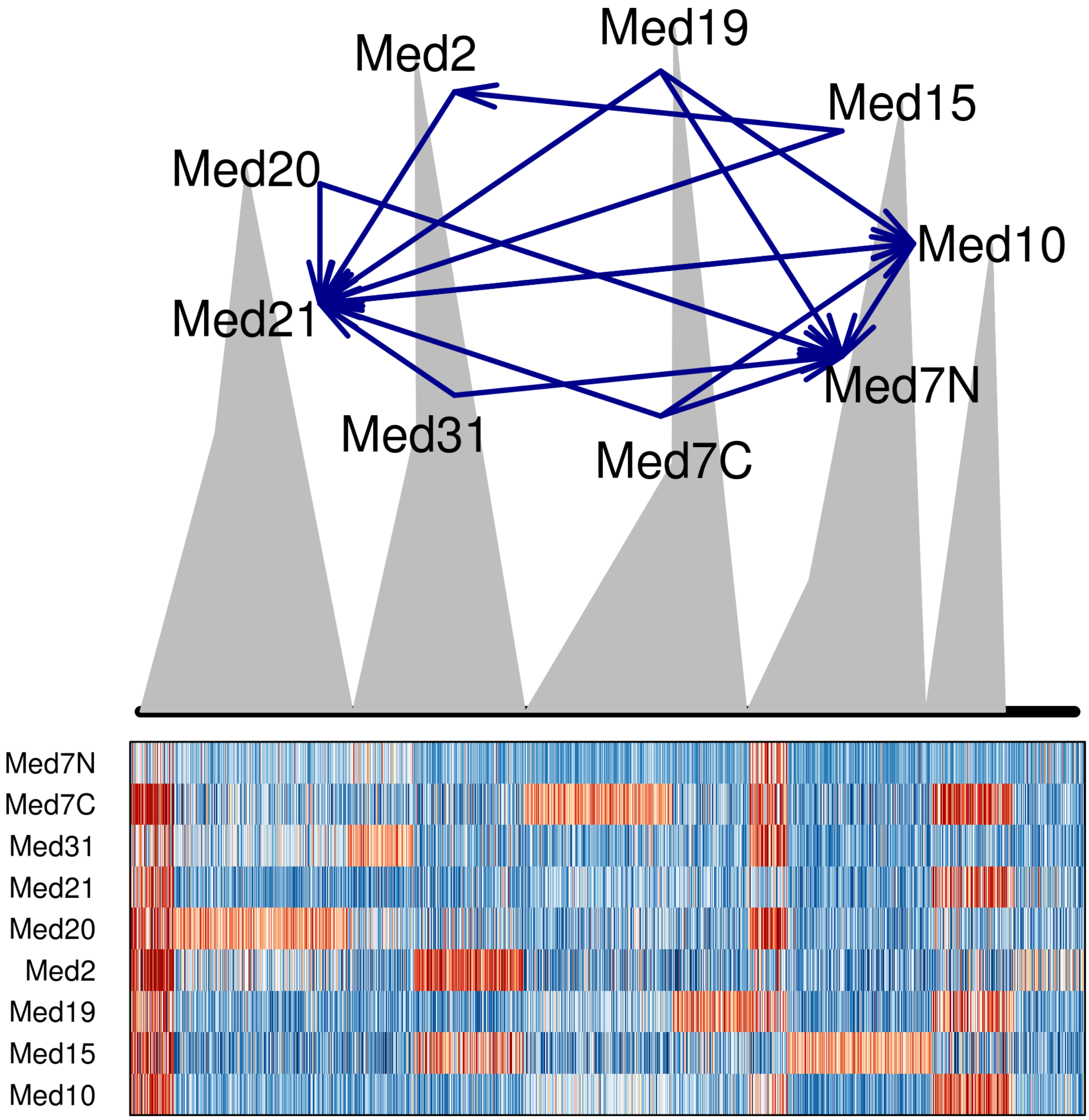

Figure 4.2. The Mediator-NEM treating all subunits as individual nodes, version 2 (result of one run out of 10). Above, the resulting signals graph is shown, below the underlying R matrix, clustered according to the final gene attachment (rows: perturbations, columns: effects on measured genes). Red color indicates a positive log-ratio value, blue color indicates a negative log-ratio value. The stronger the color of a field R_{kj}, $k \in \mathcal{E}$, $j \in \mathcal{S}$, the higher the probability that the measured data is due to the fact that there actually is an effect of signal j on gene k, or, that there is no effect, respectively. There exists an edge Med10 $\rightarrow$ Med21 as well as Med21 $\rightarrow$ Med10.The similarity between the two perturbations is also clearly visible in the perturbation profile. Thus, in the following, the two Mediator subunits are treated as one node in the NEM.

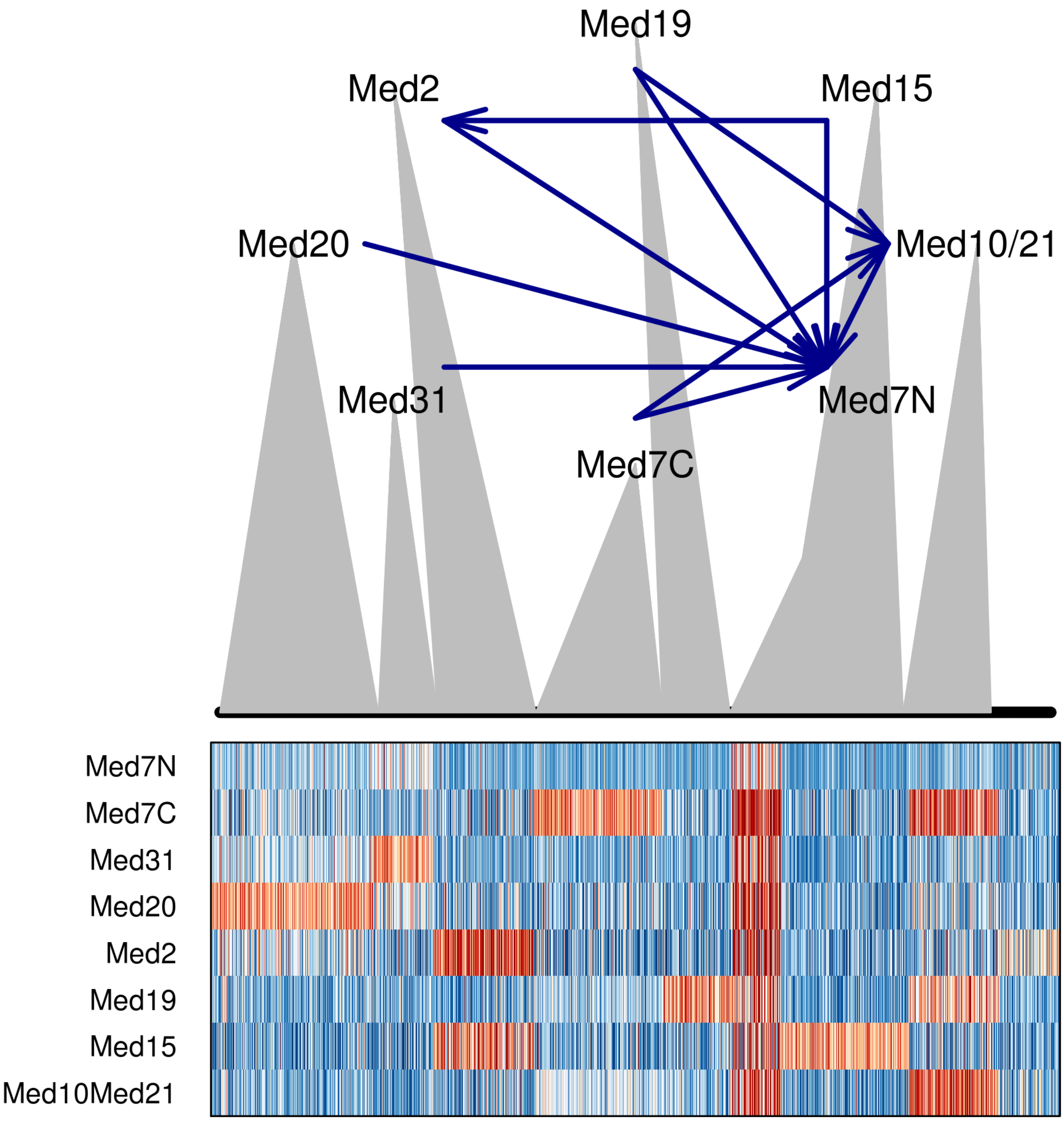

Figure 4.3. The final Mediator-NEM, where Med10 and Med21 are combined to one single node. Above, the resulting signals graph is shown, below the underlying R matrix, clustered according to the final gene attachment (rows: perturbations, columns: effects on measured genes). Red color indicates a positive log-ratio value, blue color indicates a negative log-ratio value. The stronger the color of a field R_{kj}, $k \in \mathcal{E}$, $j \in \mathcal{S}$, the higher the probability that the measured data is due to the fact that there actually is an effect of signal j on gene k, or, that there is no effect, respectively. A detailed discussion of the results can be found in the main text.

Study set	Transcription factor	in population	in study set	Estimate
Med2 - downregulated	TEC1	42	14	0.999
	YAP6	73	15	0.963
	GTS1	10	3	0.808
	SUM1	32	5	0.792
	YAP1	25	7	0.780
	SWI4	80	11	0.562
	ASH1	20	4	0.513
Med7C - downregulated	MBP1	77	22	0.991
Med7C - upregulated	RPN4	44	11	0.907
Med7N - downregulated	SWI5	51	7	0.910
	FKH2	65	11	0.901
	GLN3	63	8	0.664
	YOX1	3	1	0.510
Med10Med21 - downregulated	INO4	12	6	0.901
	STB5	14	3	0.557
Med10Med21 - upregulated	UME6	71	13	0.999
	HSF1	29	9	0.994
	HAP4	27	9	0.980
	SKN7	93	17	0.904
	SKO1	15	4	0.842
	HAP3	13	3	0.519

Table 2. Gene set enrichment analysis. This table provides the results of the gene set enrichment analysis conducted as outlined in section 4.3. First column: the studied gene set (i.e., the Mediator subunit and the direction of expression change); Second column: the number of genes in the whole population annotated to this TF; Third column: the number of genes in the study set; Fourth column: the estimate for this TF being enriched (cutoff for this study: 0.5).

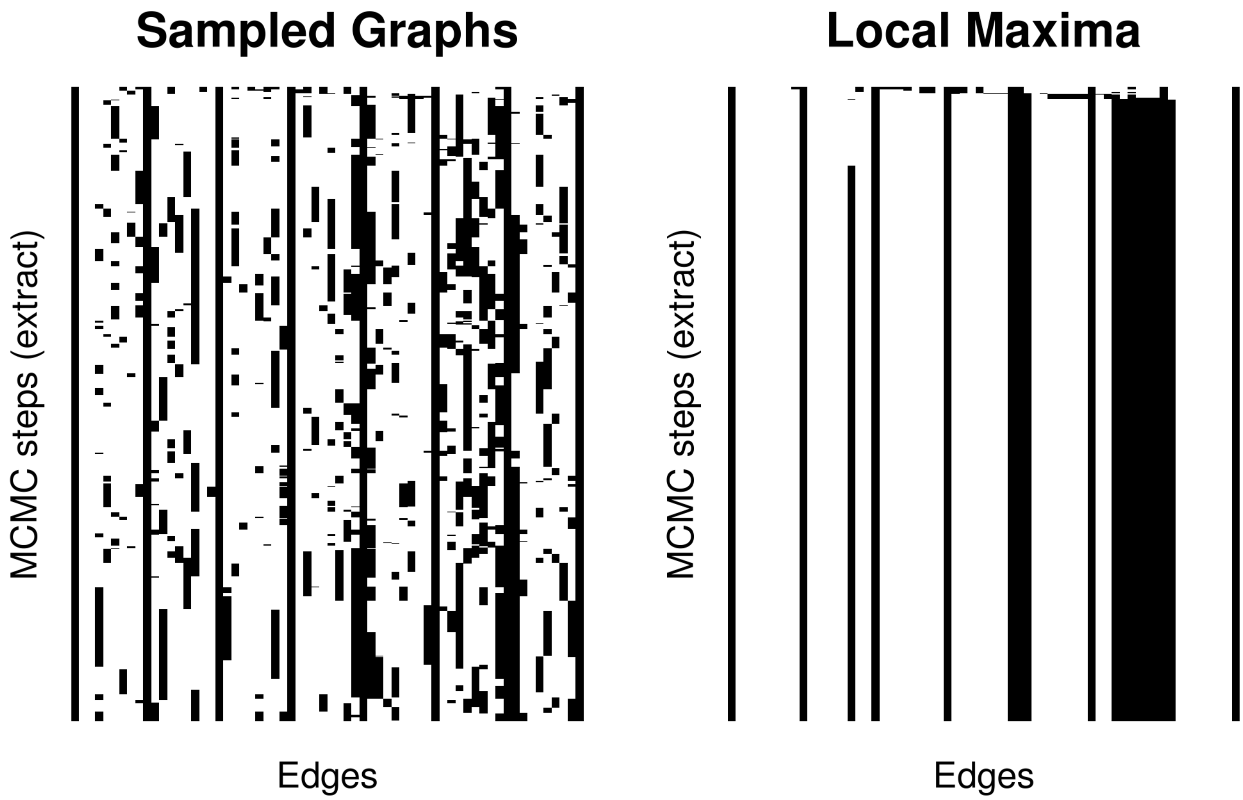

Figure 4.4. Traceplot of one MCMC run. Only the first 5000 MCMC steps are shown, since the chain converges very fast to one final signals graph (see also section S2.2). The left panel shows the traceplot for the sampled graphs $(\Theta_i)_{i=1,2,...}$, the right panel shows the traceplot for the corresponding local maxima $(\hat{\Theta}_i)_{i=1,2,...}$. The MCMC steps are depicted on the y-axis (from top to bottom), individual edges on the x-axis, thus, one line in the traceplot corresponds to the signals graph of the corresponding MCMC step. Black fields indicate the presence, white fields the absence of a given edge in a given MCMC step. Completely black columns represent self-loops, which are defined to be present in the mathematical formulation and included here for reasons of clarity. Since various signals graphs can yield the same local maxima, the sampled graphs vary strongly, while the local maxima vary slower and in a more restricted model space and converge faster. This behavior has been discussed extensively in the main text.

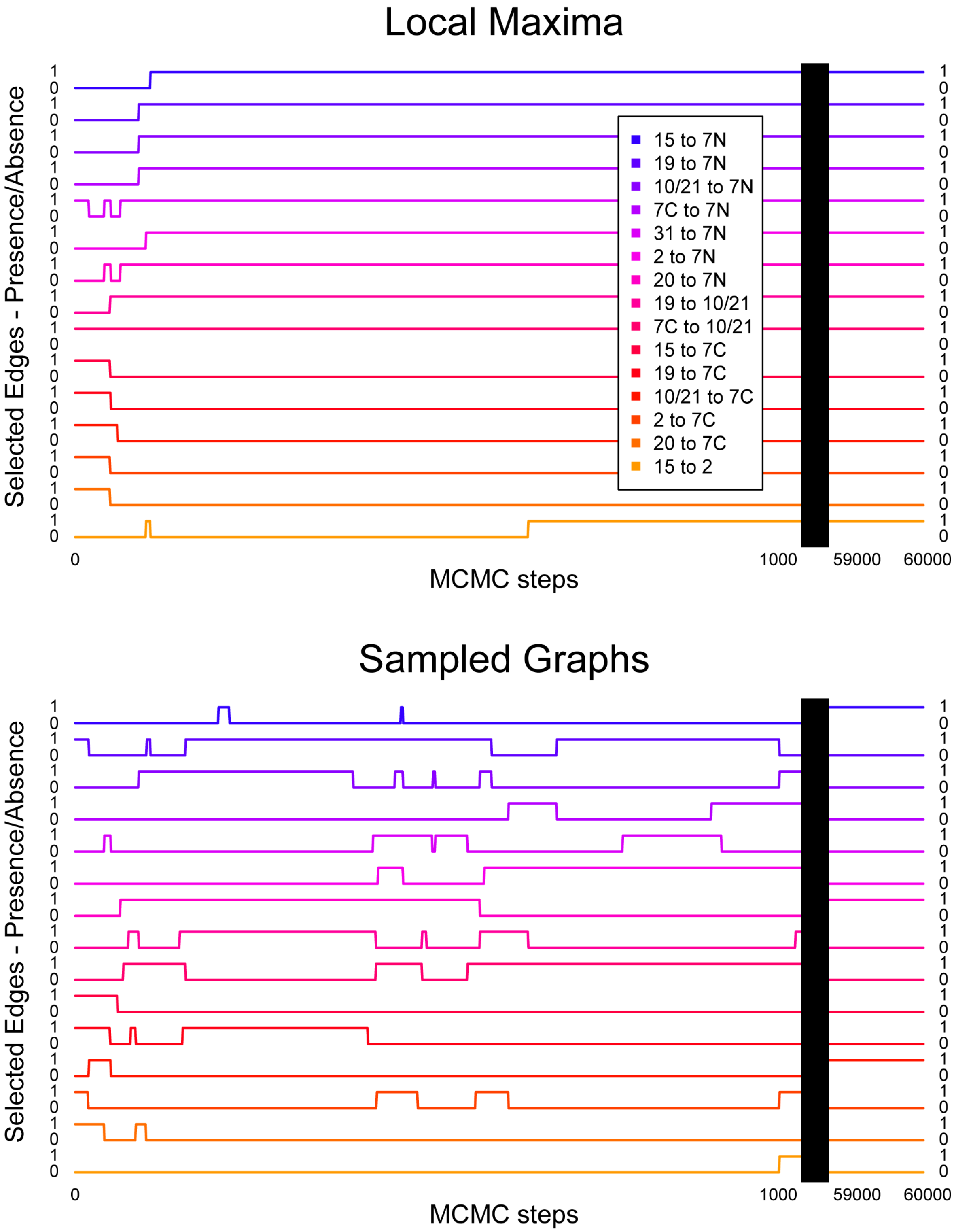

Figure 4.5. Traceplots of selected edges. The upper panel shows the traceplots of selected edges in the sequence of local maxima $(\hat{\Theta}_i)_{i=1,2,...}$, the lower panel shows the traceplot of these edges in the sequence of the underlying sampled signals graphs $(\Theta_i)_{i=1,2,...}$. On the x-axis, extracts of the MCMC steps at the beginning $(1-1000)$ and the end $(59000-60000)$ of the chain are depicted. Selected edges (edges, that appear in > 40 MCMC steps in the sequence of local maxima) are depicted in different colors. Stacked on the y-axis are values of 0 and 1 for each edge, corresponding to the absence and presence of the edge at a given MCMC-step. The traceplots here show the same behavior as has already been discussed in Fig. S4.4.

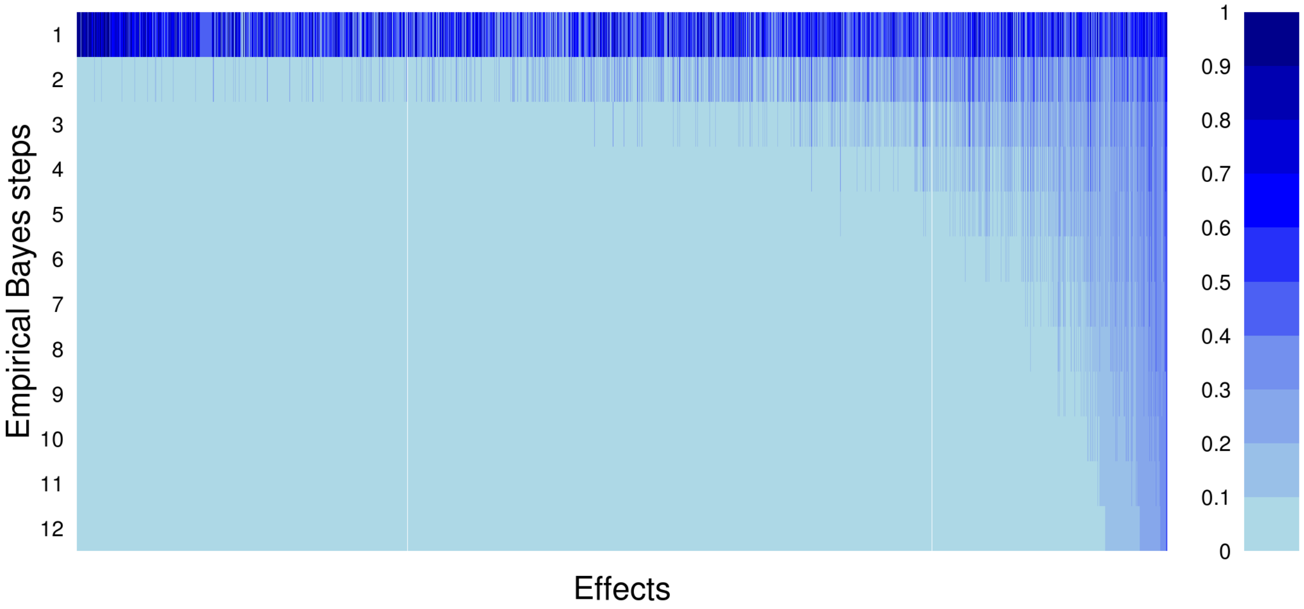

Figure 4.6. Development of attachment entropy. For each effect j in each Empirical Bayes step l, the Shannon Entropy is calculated as follows: $-\sum_{j \in \mathcal{S}} H^l_{jk} \cdot log_2 H^l_{jk}$. On the y-axis, the Empirical Bayes steps are depicted (from top to bottom), on the x-axis, the effects are listed. The colors indicate the entropy, relative to the maximal one (when, for a given effect, the attachment probability is the same for any signal node (or no signal node at all)). Obviously, the overall entropy is already much lower in the initial effects graph prior, compared to the simulation results. Furthermore, most effects showing a high entropy in the first stop, converge to a preferred attachment very fast, only few edges show no preferences.

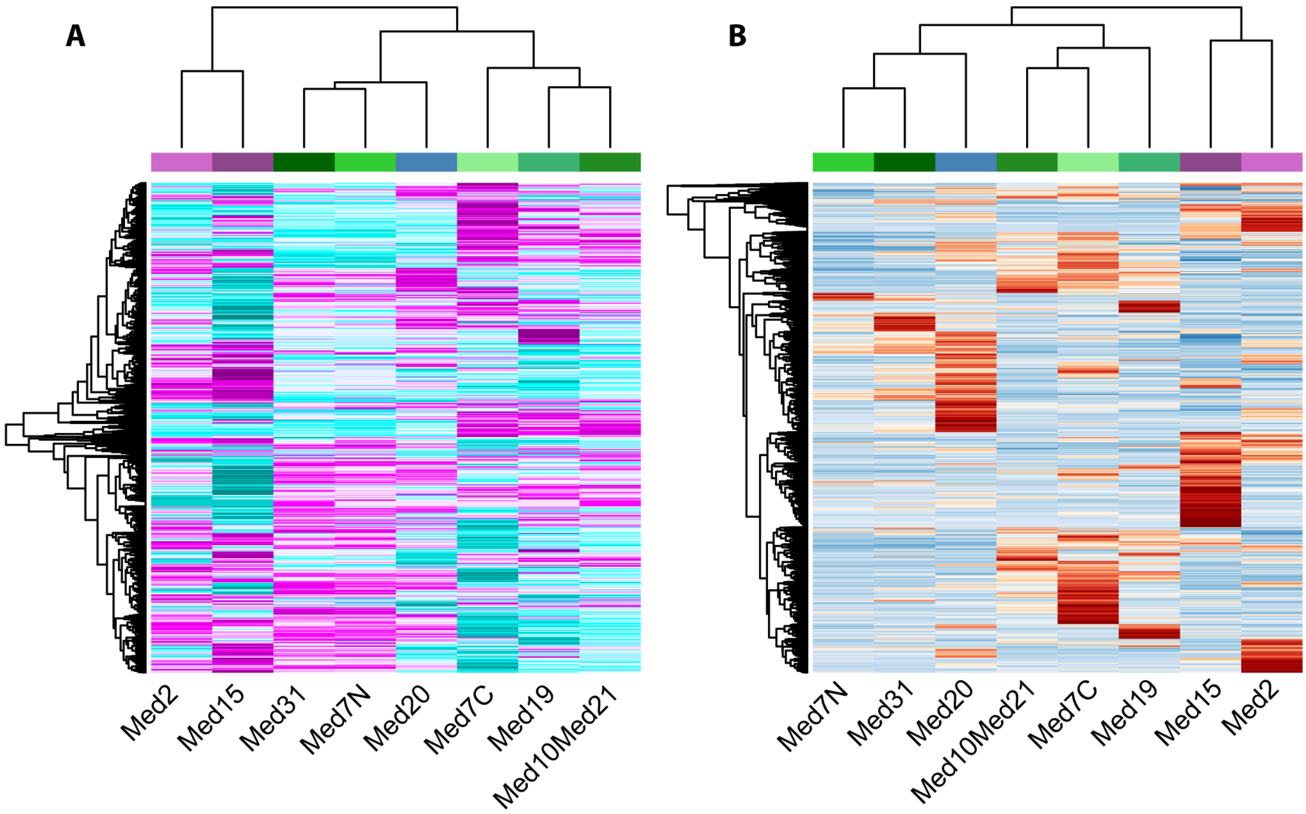

Figure 4.7. Clustering of Mediator subunits and genes based on (A) fold changes and (B) log-odds ratios. Mediator subunits are colored according to Fig. 3 and Fig. 4.

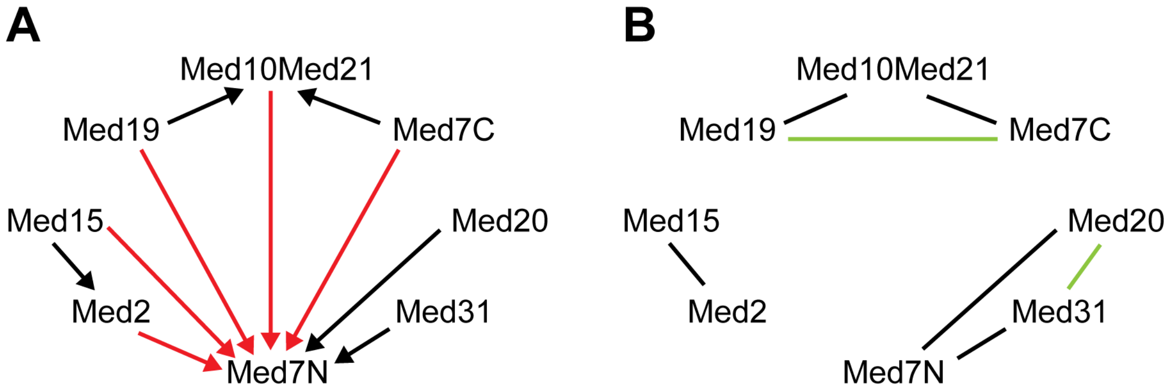

Figure 4.8. Mediator networks derived by MC EMiNEM (A) and hierarchical clustering (B). Please note that unlike MC EMiNEM the clustering approach only yields undirected edges. Edges (ignoring the direction) that appear in both networks are colored black, MC EMiNEM specific edges are colored red and clustering specific edges are colored green. In order to convert the hierarchical clustering of Fig. 4.7A into an interaction graph we need to define a cutoff level (i.e. maximum distance) below which two signal nodes are considered as interacting. We have chosen this cutoff such that the number of interactions (undirected edges, here: 7) is as close as possible to the number of directed edges in our NEM (10 edges, Fig. 3). The need to define a manually adjusted cutoff is one of the major drawbacks of a clustering approach, making the results elusive and arbitrarily. Furthermore, MC EMiNEM offers a more refined resolution, being able to overcome the Mediator modules.

```
# load nem package
library(nem)
# load Mediator data
data("NiederbergerMediator2012");
lodsmat = NiederbergerMediatorLods;
nr_signals = ncol(lodsmat)
nr_effects = nrow(lodsmat)
# randomly create an initial signals graph
theta_init = matrix(sample(c(0,1),nr_signals^2,replace=TRUE,prob=c(1-1/nr_signals,
        1/nr_signals),nrow=nr_signals,ncol=nr_signals); diag(theta_init)=1;
colnames(theta_init) = colnames(lodsmat);
rownames(theta_init) = colnames(lodsmat);
models = list();
models[[1]] = theta_init;
# calculate the data-driven prior
effects_prior = prior.EgeneAttach.EB(lodsmat)
# set the parameters
control = set.default.parameters(Sgenes=colnames(lodsmat), type="CONTmLLBayes",
      mcmc.nsamples=5000, mcmc.nburnin=15000, Pe=effects_prior,
      eminem.sdVal=ceiling(1.5*ncol(lodsmat)), eminem.changeHfreq=5000,
      Pm.frac_edges=1/ncol(lodsmat), lambda=0.5);
# start estimation process and visualize results
net = nem(lodsmat, inference="mc.eminem", models=models, control=control);
plot(net);
```

A short introduction to MC EMiNEM is provided above (see the nem package vignette for more details). It also lists all parameters that have to be set in order to run MC EMiNEM: *type* and *inference* define the method to be used (here: MC EMiNEM); *Sgenes* (the signal names) is defined by the input data; *Pe* (the effects graph prior) and *Pm.frac_ edges* (the sparsity prior) incorporate prior knowledge and are no parameters in the proper sense; *mcmc.nsamples* (the length of the stationary phase), *mcmc.nburnin* (the length of the burn-in phase), *eminem.sdVal* (the width of the proposal function) and *eminem.changeHfreq* (the Empirical Bayes parameter) only influence the length of the Markov chain and its mixing properties, i.e., as long as the chain is long enough, they do not affect the final distribution. Hence, the only parameter that has to be adjusted is the weight of the sparsity prior *lambda*. As already discussed in the Supplementary Section S2.2, the sparsity prior itself is important, but moderate variation of *lambda* did not change the results qualitatively.

References

1. Tresch A, Markowetz F (2008) Structure learning in Nested Effects Models. Stat Appl Genet Mol Biol 7: Article9.
2. Dempster AP, Laird NM, Rubin DB (1977) Maximum Likelihood from Incomplete Data via the EM Algorithm. Journal of the Royal Statistical Society Series B (Methodological) 39: pp. 1-38.
3. Minka TP (1998). Expectation-Maximization as lower bound maximization.
4. Hastings WK (1970) Monte Carlo sampling methods using Markov chains and their applications. Biometrika 57: 97-109.
5. Andrieu C, de Freitas N, Doucet A, Jordan MI (2003) An Introduction to MCMC for Machine Learning. Machine Learning 50: 5-43.
6. MacKay D (2003) Information theory, inference, and learning algorithms. Cambridge University Press.

7. Tresch A (2007) Nessy: NESted effects models for SYstems biology. URL `http://www.tresch.genzentrum.lmu.de/Nessy_Stuff`. R package version 1.0.
8. Markowetz F, Bloch J, Spang R (2005) Non-transcriptional pathway features reconstructed from secondary effects of RNA interference. Bioinformatics 21: 4026–4032.
9. Fröhlich H, Tresch A, Beissbarth T (2009) Nested effects models for learning signaling networks from perturbation data. Biom J 51: 304–323.
10. Koschubs T, Seizl M, Larivière L, Kurth F, Baumli S, et al. (2009) Identification, structure, and functional requirement of the Mediator submodule Med7N/31. EMBO J 28: 69–80.
11. Singh H, Erkine AM, Kremer SB, Duttweiler HM, Davis DA, et al. (2006) A functional module of yeast mediator that governs the dynamic range of heat-shock gene expression. Genetics 172: 2169–2184.
12. Team RDC (2011) R: A Language and Environment for Statistical Computing. R Foundation for Statistical Computing, Vienna, Austria. URL `http://www.R-project.org`. ISBN 3-900051-07-0.
13. Gautier L, Cope L, Bolstad BM, Irizarry RA (2004) affy–analysis of Affymetrix GeneChip data at the probe level. Bioinformatics 20: 307–315.
14. Smyth GK (2005) Limma: linear models for microarray data. In: Gentleman R, Carey V, Dudoit S, R Irizarry WH, editors, Bioinformatics and Computational Biology Solutions using R and Bioconductor, New York: Springer. pp. 397–420.
15. Bauer S, Gagneur J, Robinson PN (2010) GOing Bayesian: model-based gene set analysis of genome-scale data. Nucleic Acids Res 38: 3523–3532.
16. Bauer S, Robinson PN, Gagneur J (2011) Model-based gene set analysis for Bioconductor. Bioinformatics 27: 1882–1883.
